# Genetic dissection of seed oil and protein content and identification of networks associated with oil content in *Brassica napus*

**DOI:** 10.1038/srep46295

**Published:** 2017-04-10

**Authors:** Hongbo Chao, Hao Wang, Xiaodong Wang, Liangxing Guo, Jianwei Gu, Weiguo Zhao, Baojun Li, Dengyan Chen, Nadia Raboanatahiry, Maoteng Li

**Affiliations:** 1 Department of Biotechnology, College of Life Science and Technology, Huazhong University of Science and Technology, Wuhan, 430074, China; 2 Hybrid Rapeseed Research Center of Shaanxi Province, Shaanxi Rapeseed Branch of National Centre for Oil Crops Genetic Improvement, Yangling, 712100, China; 3 Hubei Collaborative Innovation Center for the Characteristic Resources Exploitation of Dabie Mountains, Huanggang Normal University, Huanggang, 438000, China; 4 Key Laboratory of Cotton and Rapeseed, Ministry of Agriculture, Institute of Industrial Crops, Jiangsu Academy of Agricultural Sciences, Nanjing, 210014, China

## Abstract

High-density linkage maps can improve the precision of QTL localization. A high-density SNP-based linkage map containing 3207 markers covering 3072.7 cM of the *Brassica napus* genome was constructed in the KenC-8 × N53-2 (KNDH) population. A total of 67 and 38 QTLs for seed oil and protein content were identified with an average confidence interval of 5.26 and 4.38 cM, which could explain up to 22.24% and 27.48% of the phenotypic variation, respectively. Thirty-eight associated genomic regions from BSA overlapped with and/or narrowed the SOC-QTLs, further confirming the QTL mapping results based on the high-density linkage map. Potential candidates related to acyl-lipid and seed storage underlying SOC and SPC, respectively, were identified and analyzed, among which six were checked and showed expression differences between the two parents during different embryonic developmental periods. A large primary carbohydrate pathway based on potential candidates underlying SOC- and SPC-QTLs, and interaction networks based on potential candidates underlying SOC-QTLs, was constructed to dissect the complex mechanism based on metabolic and gene regulatory features, respectively. Accurate QTL mapping and potential candidates identified based on high-density linkage map and BSA analyses provide new insights into the complex genetic mechanism of oil and protein accumulation in the seeds of rapeseed.

Rapeseed (*Brassica napus* L., AACC, 2n = 38) is one of the most important oil crops globally. It originated from a spontaneous hybridization between *B. rapa* (AA, 2n = 20) and *B. oleracea* (CC, 2n = 18)^1^. The large amount of unsaturated fatty acid (FA) in rapeseed oil has made it widely accepted as a vegetable oil for human consumption and as a bio-fuel for industry^2^. The low glucosinolate and high protein content of the seed also make it a valuable feedstuff for animals and a potential protein source for human nutrition^3^,^4^. Increasing the seed oil content (SOC) and revealing the complex regulatory mechanism of SOC and the seed protein content (SPC) have become the most important breeding criteria for rapeseed. However, SOC and SPC are two complex quantitative traits that are frequently influenced by the environment^5^, and the genetic and molecular bases of both traits remain ambiguous.

QTL mapping is an effective strategy to dissect the regulatory loci and genetic mechanism underlying important agronomical traits at the whole genome level. Many studies have focused on SOC-QTL identification in *Brassica napus (B. napus*). SOC-QTLs were identified in almost all 19 linkage groups based on bi-parental linkage mapping, and varied between 3 and 27 QTLs^6^,^7^,^8^,^9^,^10^,^11^,^12^,^13^. Additionally, a genome‑wide association study has revealed SOC variations in *B. napus*^14^. Compared with SOC, relatively few studies have addressed SPC-QTLs in *B. napus*^15^,^16^,^17^,^18^. In addition, a significant negative relationship between SOC and SPC has been reported, especially in soybean^19^,^20^ and Brassica^21^,^22^,^23^. QTLs with pleiotropic effects for SOC and SPC have also been observed in Brassica species^15^,^24^. Identifying the underlying candidate genes and recognizing the pleiotropic effect or correlation between these two traits would greatly increase the breeding efficiency.

Nevertheless, in most of these studies, the QTLs spanned large ranges of the genome due to low mapping resolutions, and generally there was a lack of marker sequence information, hindering the integration of these QTLs into the reference physical map and the identification of the underlying candidate genes. In addition, the genomic sequences of the parental cultivars were also unknown in most of the previous studies, which also complicated the identification and analysis of candidate genes. Recent innovations in genome sequencing technology and bioinformatics and single nucleotide polymorphism (SNP) markers, especially the 60 K SNP Infinium Array, have been developed and widely applied for QTL mapping and the genetic dissection of important agronomical traits in *B. napus*^25^,^26^,^27^,^28^,^29^,^30^,^31^. Simultaneously, the reference genome of *B. napus* has been released^32^, making it feasible to perform a comparative genome analysis between the genetic map and the physical map using SNPs with sequence information. Additionally, bulk segregant analysis (BSA) combined with next-genomic sequencing technologies has been used as a fast track approach to more rapidly identify candidate genomic regions. This approach involves the selection of 20–50 lines with extreme phenotypic values, with pooling at equivalent concentrations followed by sequencing of the pools for subsequent variance analysis^33^. BSA has been used successfully to map candidate genomic regions for early flowering in cucumber^34^, 100-seed weight in chickpea^35^, and fruit weight and locule number in tomato^36^.

QTL studies have led to the identification of numerous loci that are responsible for variation in SOC and SPC, but the corresponding genomic regions with their complex metabolism and regulatory mechanisms have not been studied with great accuracy. Therefore, candidate genes within QTL regions combined with the relevant metabolic pathway in *B. napus* have been not reported. There is extensive genome homology and microsynteny between *Brassica* species and *Arabidopsis thaliana (A. thaliana*) because of their close evolutionary relationship^37^. More attention has been focused on genes and regulatory factors involved in acyl-lipid metabolism, and more than 120 enzymatic reactions and at least 600 genes involved in acyl-lipid metabolism in *A. thaliana* have been well documented by Li-Beisson *et al*.^38^. *A. thaliana* acyl-lipid and storage protein-related orthologous genes mapped in a confidence interval (CI) of QTLs on the *B. napus* genome might be valuable to identify the candidate genes and dissect the complex metabolism and regulatory mechanism associated with SOC and SPC.

This present report consists of the following three parts: (1) the construction of a high-density genetic linkage map based upon SNP markers combined with non-SNP (SSR and STS) markers that have been used previously to generate a primary linkage map in the KenC-8 × N53-2 (KNDH) population^39^; (2) precise QTL mapping of SOC and SPC across multiple environments based on a high-density genetic linkage map and rapid detection of associated genomic regions (AGRs) using BSA; (3) identification of candidate genes within QTL regions and provision of primary insights into the complex metabolism and regulatory mechanism of SOC and SPC in *B. napus*.

## Results

### Construction of the high-density linkage map

**Figure 1:**
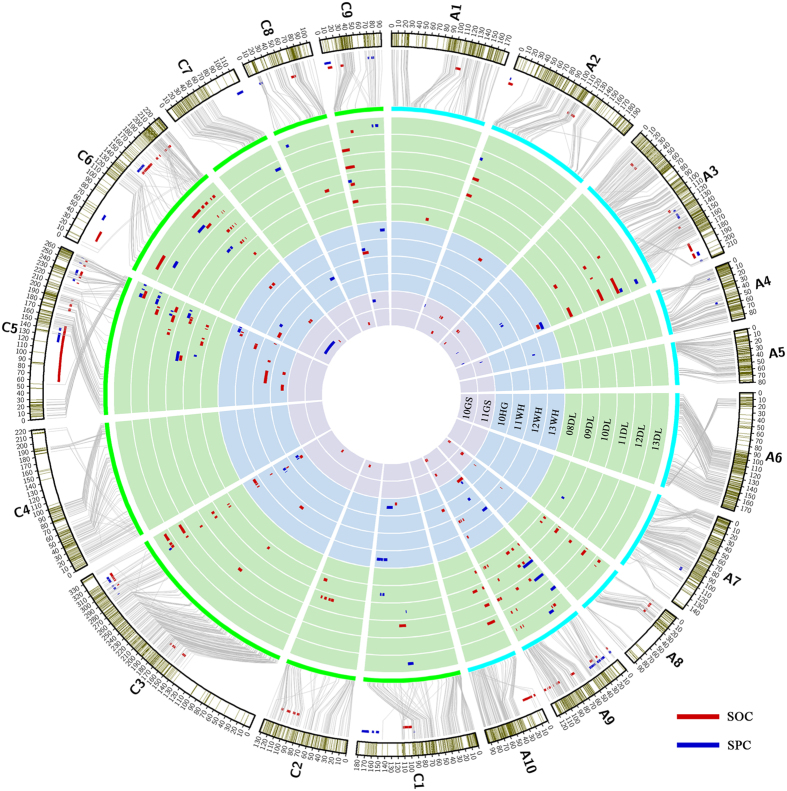
KN high-density genetic linkage map, distribution of markers, identified QTLs, consensus QTLs of SOC and SPC on each linkage group, and colinearity of the linkage map and *B. napus* reference genome. The blocks with green bands at the outermost circle represent the 19 genetic linkage groups and the marker distribution. The third circle (from the outermost one) represents the 19 chromosomes of *B. napus*. The lines connecting them represent their col-linearity relationship. The 12 inner circles with 3 colors represent 12 microenvironments in winter-type, spring-type and semi winter-type rapeseed growing areas, respectively. The short bars with a red and blue color within the 12 inner circles represent QTLs identified in different environments and linkage groups, and they are located between the outermost two circles representing consensus QTLs.

Of the 52157 SNP probes on the *Brassica* 60 K array, approximately 34.72% were polymorphic between the two parents and displayed segregation within the KNDH population. Finally, 18109 polymorphic SNPs were grouped into 3764 SNP-bins. All 3764 SNP-bins merged with 495 non-SNP markers (SSR and STS) were used for linkage map construction. A set of 3207 markers including 3106 SNP-bins and 101 non-SNPs were successfully assigned to 19 linkage groups (Table 1). The integrated map had a total length of 3072.7 cM with an average distance of 0.96 cM between adjacent markers (covering 1398.6 cM and 1674.1 cM for the A and C genomes, respectively). The number of loci in the 19 linkage groups ranged from 77 (A08) to 324 (A03), and the length ranged from 80.1 (A05) to 337.1 cM (C03), with a mean value of 161.72 cM (Table 1, and Fig. 1). The marker density of the 19 linkage groups varied; the highest marker density was on chromosome A04, with 156 markers distributed over a genetic map distance of 86.8 cM.

### Phenotypic variation and correlation of SOC and SPC in the parents and the DH population

**Figure 2:**
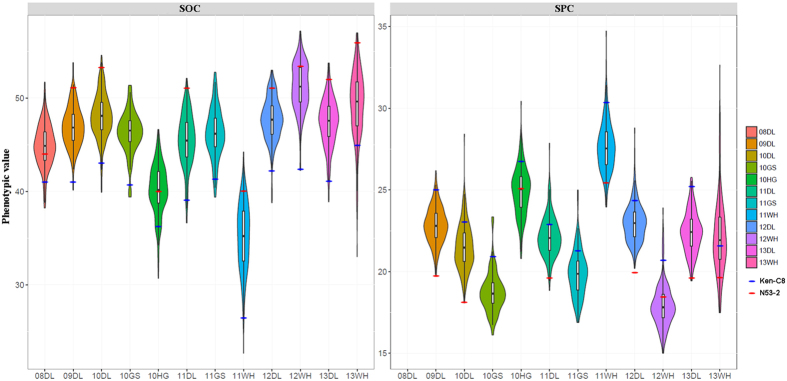
Frequency distribution of SOC and SPC in the KNDH population. In each plot, a marker denotes the median of the data, a box indicates the interquartile range, and spikes extend to the upper and lower adjacent values. The distribution density is overlaid.

Combined with the data that were previously reported by Wang *et al*.^39^, a total of 12 and 11 sets of SOC and SPC trial data were used to analyze the phenotypic variation (PV), respectively (Table S1). The SOC and SPC of the two parents (N53-2 and KenC-8) differed significantly in all investigated environments. The SOC of N53-2 was 10% higher than that of Ken-C8 in all microenvironments except 10HG. While the SPC of Ken-C8 was nearly 5% higher than that of N53-2 in all semi-winter environments, they also showed significant differences, though to a reduced extent for the winter environments. The KN population exhibited a normal or near-normal distribution and transgressive segregation with a small degree of both traits (Fig. 2). These results indicated that quantitative inheritance was suitable for QTL identification, with polygenic effects and favorable alleles mainly inherited from one of the two parents. Further analysis revealed that both parents and populations in different trials all showed significant differences (Table S1), suggesting that both traits were significantly influenced by the environment. In addition, SOC and SPC were significantly negatively correlated with average coefficients of −0.65; the highest correlation up to −0.77 was identified in the 11GS (Table S2), suggesting competition among sinks for assimilates.

### Identification, integration and co-localization analysis of QTLs for SOC and SPC

A total of 164 identified QTLs were observed for SOC in 12 trials, with an average CI of 7.43 cM. These QTLs were distributed on 12 chromosomes, excluding A04, A05, A06, A07, C01, C04 and C07, and a single QTL could explain up to 22.24% of the PV. The largest number of QTLs (136) was distributed on A03, A08, A09, C03, C05 and C06, accounting for 82.93% of the total number (Fig. 1 and Table S3). The 164 identified SOC-QTLs were then integrated into 67 consensus QTLs, and the average CI was reduced from 7.36 to 5.26 cM (Tables 2 and S3 and Fig. 1). Fifty-three consensus QTLs were distributed on A03, A08, A09, C03, C05 and C06, accounting for 79.10% of all consensus QTLs, and 35 of them (76.09%) showed environmental stability. There were 59, 30, and 16 QTLs detected in winter type, semi-winter type and spring type environments, respectively (Figure S1a). Only one QTL (*cqOC-A9-9*) could be detected in all three macroenvironments. Eleven, two and one QTL could be expressed stably at least over 2 years in winter, semi-winter and spring type environments, respectively. Six major QTLs (*cqOC-A2-3, cqOC-A9-9, cqOC-A9-10, cqOC-C5-3, cqOC-C5-4,* and *cqOC-C6-6*) were repeatedly detected in at least two trials and displayed a large effect (PV > 10%), among which *cqOC-A9-9* and *cqOC-C6-6* were detected stably in six microenvironments, while *cqOC-C6-6* explained the highest PV (22.24%). Although accounting for less than 10% of the PV value, *cqOC-C3-4* could be detected simultaneously in eight microenvironments and was considered a major QTL that merits attention.

A total of 68 identified QTLs for SPC were observed in 11 microenvironments, which were distributed on 12 chromosomes and accounted for 2.19–27.48% of PV with an average CI of 9.31 cM (Fig. 1 and Table S3). The same results were obtained for SPC-QTLs, most of which (64.71%) were distributed on A03, A09, C03 and C05, with the difference that none of the SPC-QTLs were detected on A08. The identified SPC-QTLs were then integrated into 38 consensus QTLs with a narrower average CI of 4.38 cM (Table S3 and Fig. 1). Twenty-three QTLs could be detected in at least two microenvironments, and *cqPC-C5-6* could be identified in four microenvironments, while five QTLs (*cqPC-A3-4, cqPC-A9-2, cqPC-C1-2, cqPC-C5-5* and *cqPC-C5-7*) were identified in three microenvironments. None of the consensus QTLs could be detected in all three macroenvironments, and six and one QTL could be expressed stably at least over 2 years in winter and semi-winter type environments, respectively (Fig. S1b). Four QTLs, *cqPC-A2* and *cqPC-C1-2*, which were repeatedly detected in at least two trials with a medial effect (20% > PV > 10%), and *cqPC-A3-2* and *cqPC-A3-5*, which were detected in one trial but had a large effect (PV > 20%), were also treated as major QTLs.

In addition, as observed from the additive effect (Table 2), the N53-2 alleles in the majority of SOC-QTLs (81.25%) increased SOC, but most SPC-QTLs (81.58%) decreased SPC, consistent with the higher SOC and lower SPC of N53-2 compared with Ken-C8 (Table S1).

The close genetic relationship between SOC and SPC could be confirmed by mapping the co-localized loci. All 11 co-localized QTLs affected both traits and showed opposite additive effects (Table 3), and the favorable alleles of the 10 co-localized QTLs that were derived from the high oil content parent N53-2 could increase SOC and decrease SPC, with only *uqC5-1* demonstrating the opposite. Close co-localization explained the high genetically negative correlation between SOC and SPC, and suggested a complexity of the competitive mechanism of these two traits.

Additionally, some QTL clusters associated with both traits distributed on chromosomes A09, C03 and C05 were also detected. For example, one of these clusters falling in interval from 287.50–322.64 cM on C03 influenced SOC and SPC with consistently negative effects (Figure S2). These results indicated that tight linkage was also an important factor in addition to pleiotropy for their competitive relationship. In addition, there were SOC-QTL clusters, but none of the SPC-QTLs were found on A08, which suggested that more allelic variations control SOC compared with SPC on chromosome A08 between two parents.

### BSA analysis based on extreme bulks and confirmation of QTL mapping

Twenty-four DH lines with high SOC (47.2–52.7%) and 24 with low SOC (38.0–42.4%) were selected to prepare two extreme bulks. As a significant negative correlation of SOC and SPC, high and low SOC bulks corresponded to low (19.8–23.9%) and high (22.5–27.2%) SPC, respectively.

Illumina high-throughput sequencing was used to generate 274.08 million and 274.85 million reads from the low and the high SOC bulks, with a coverage of 84.30% (35.85-fold) and 90.74% (39.00-fold) of the reference genome of *B. napus*, respectively. In contrast, 97.47% (17.16-fold) and 96.56% (19.82-fold) genome coverage was obtained for the two parents, KenC-8 (low SOC and high SPC) and N53-2 (high SOC and low SPC), confirming the accuracy of the subsequent analysis (Table S4). The graph of Δ (SNP-index) was generated (Figure S3) using the genome sequence of KenC-8 as a reference.

**Figure 3:**
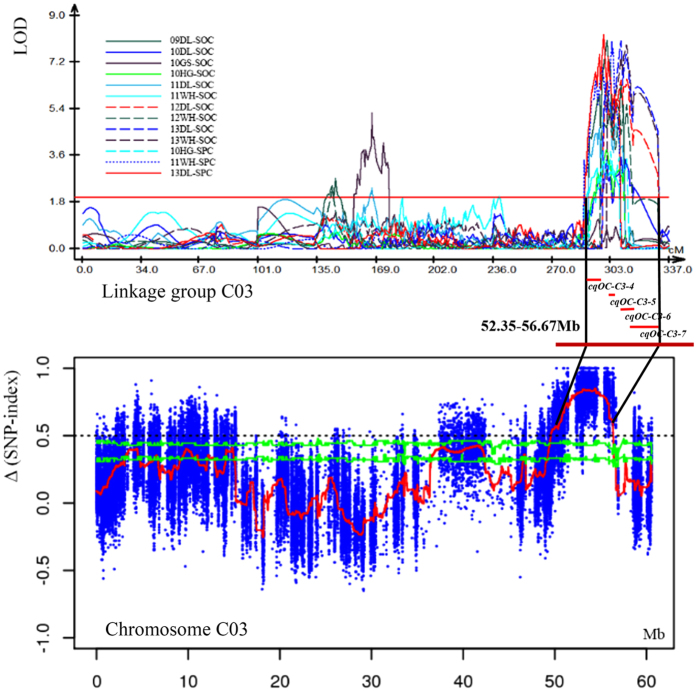
Overlapping region of AGR and SOC-QTL hotspots on chromosome C03. Original QTL identification in the different experiments shown by curves above the line of the linkage group (top). The Δ(SNP-index) plot with statistically significantly associated regions (two green significant threshold lines, P < 0.01 and P < 0.05) is drawn at the bottom. The *X*-axis represents the position of chromosome C03, and the *Y*-axis represents Δ(SNP-index). An overlapping region of 52.35–56.67 Mb is drawn in the middle, and the small red bars under the linkage group line within QTL hotspots represent consensus QTLs that overlap with AGR.

One hundred and thirty-three AGRs underlying SOC were identified on 17 chromosomes (apart from A01 and C07) (Table S5), and 38 AGRs overlapped and/or narrowed the SOC-QTLs from QTL mapping based on the present genetic linkage map (Table S6). The major QTL *cqOC-A9-9* was validated by a significant region *AGR_A9-8*, and the major QTL *cqOC-A9-10* was divided into three narrower AGRs (*AGR_A9-9, AGR_A9-10* and *AGR_A9-11*). It is noteworthy that some AGRs with a significantly high Δ (SNP-index) and QTL hotspots were found to co-localize on chromosome A03, A09, C03, and C05 (Table S6). For example, the associated genomic region (AGR_C3-16) was identified to overlap with four SOC-QTLs (*cqOC-C3-4, cqOC-C3-5, cqOC-C3-6* and *cqOC-C3-7*) that could be stably expressed in at least two microenvironments, among which *cqOC-C3-4* could be assessed stably in eight microenvironments (Fig. 3).

### Identification and analysis of potential candidates related to acyl-lipid metabolism and seed storage underlying SOC and SPC, respectively

Accurate QTLs for SOC were identified using a high-density linkage map and confirmed by BSA. Through comparative mapping between the linkage map and the physical maps of *B. napus* based on SNP sequence information, a satisfactory colinearity relationship between the genetic linkage map and the reference genome of *B. napus* was validated (Fig. 1), which could help us to accurately identify genomic regions and the corresponding candidate genes underlying these QTLs. For example, SOC-QTL *cqOC-A9-9* could be stably identified in six microenvironments, explaining up to 14.94% of the PV with 0.98 cM of CI, which was mapped within a 0.07 Mb region in the reference genome (Table S7). Finally, 59 genomic regions of SOC-QTLs (88.06%) and 29 genomic regions of SPC-QTLs (76.32%) could be determined accurately (Table S7).

A total of 448 genes involved in 21 of 22 acyl-lipid pathways were identified within 41 SOC-QTL regions (Figures S4 and S5, and Table S8). Additionally, a whole genome search was implemented to determine the distribution of orthologues related to acyl-lipid metabolism, and a total of 3058 orthologues of *A. thaliana* acyl-related genes were identified in the whole genome (Figures S4 and S5). Minor acyl-lipid related gene enrichment / prevalence in the CI of SOC-QTLs was found compared to the whole genome (Table S9). For example, six orthologues of *AtLACS9* that encoded long-chain Acyl-CoA synthetase (plastidial) were distributed on the chromosome of the *B. napus* genome, four of which displayed SNP/InDel variation in the SOC-QTL regions. Moreover, four of eight orthologues of *AtFAD6* that encoded oleate desaturase fell within SOC-QTL regions and exhibited SNP/InDel variation. According to the re-sequencing results of the two parents from BSA, 355 of 449 acyl-lipid related orthologues had SNP/InDel variation between the two parents (Table S10). These genes were involved in 21 of 22 acyl-lipid metabolism pathways. The 25, 13, 24, 20 and 9 genes among them were involved in FA synthesis in plastids, FA elongation, the synthesis of triacylglycerol (TAG), TAG degradation and beta-oxidation, respectively, accounting for 24.20% of the total potential candidates associated with lipid metabolism within SOC-QTL regions (Figure S6 and Table S10). Additionally, a large proportion of genes involved in lipid signaling and cuticular wax syntheses were mapped, accounting for 11.0% and 19.0% of the total acyl-lipid orthologous genes. This finding supported the complexity of the oil accumulation, which was also likely regulated by some genes related to lipid signaling, and the cuticular wax biosynthesis pathway probably also played a potential role in the accumulation of oil during embryonic development.

Two and eleven potential candidates related to oil body structural protein and seed storage protein were identified in 12 SPC-QTL regions (Table S11). *BnaA09g02110D*, annotated as an orthologue of oleosin 4, was located in an overlapping region of *cqPC-A9-2* and *cqOC-A9-2*. In addition, seven seed storage protein-related orthologues (*BnaA09g13220D, BnaA09g11520D, BnaC03g65080D, BnaC05g02160D, BnaC05g44560D, BnaC05g44510D* and *BnaC06g18820D*) co-localized within the SOC and SPC-QTL interval.

It is also noteworthy that some important potential candidates related to acyl-lipid metabolism were identified in overlapping regions of SOC-QTLs and AGRs of BSA (Table S10). *BnaA09g48250D*, a homologue of *AtCAC2* involved in the key steps of de novo FA synthesis, was identified in *AGR_A9-12* and the major QTL *cqOC-A9-10*, and it contained a nonsynonymous SNP in an exon. *BnaA08g12780D*, corresponding to *AtFAD6* encoding oleate desaturase, was located in *AGR_A8-4* and *cqOC-A8-3.* Twenty-two potential candidates related to acyl-lipid metabolism were identified within three major QTLs overlapping with AGRs: *cqOC-A9-9* (1), *cqOC-A9-10* (14) and *cqOC-C5-4*(7) (Table S10). Three genes (*BnaA09g48250D, BnaC05g21140D* and *BnaC05g24510D*) involved in plastidial FA synthesis, and two genes (*BnaA09g51530D* and *BnaC05g21070D*) involved in FA elongation were identified in the these regions.

**Figure 4:**
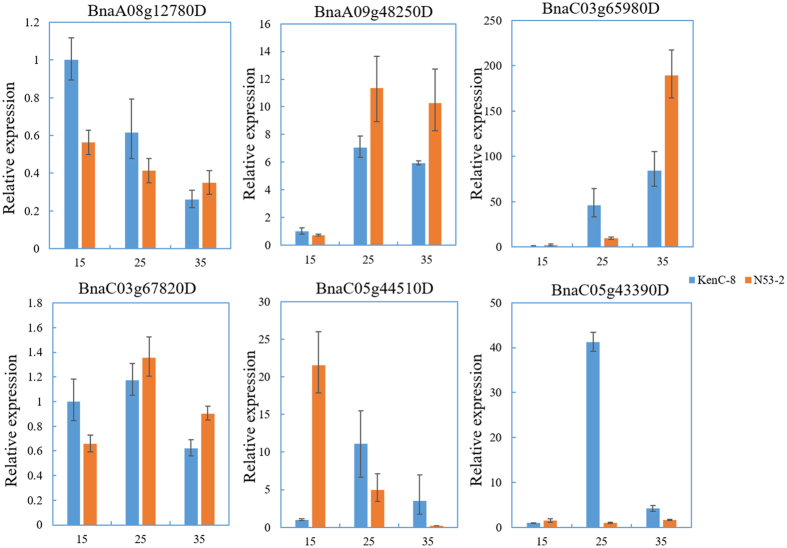
qRT-PCR results for six potential candidates. Blue bars represent KenC-8, and orange bars represent N53-2. The *X*-axis indicates 15, 25, and 35 days after flowering, whereas the *Y*-axis indicates relative expression levels.

Six potential candidates (*BnaA08g12780D, BnaA09g48250D, BnaC03g65980D, BnaC03g67820D, BnaC05g43390D* and *BnaC05g44510D*) that were reported to be involved in acyl-lipid metabolism in *B. napus* or *A. thaliana* were selected to measure the expression levels in the developing seed at 15, 25, and 35 days after flowering from both parents. All six genes showed significant expression difference between parents during different developmental periods (Fig. 4). The relative expression level of *BnaA09g48250D*, which was homologous to the *AtCAC2* gene encoding *BC* (subunit of *ACCase*), from the overlapping region *of AGR_A9-12* and the major QTL *cqOC-A9-10* increased significantly in both parents and were clearly higher in N53-2 during the middle and late embryonic development periods. *BnaA08g12780D* and *BnaC03g67820D,* which are homologous to *AtFAD6* (oleata desaturase), were determined from *cqOC-A8-3* and *cqOC-C3-7*, their relative expression level was reduced but higher in N53-2 during the late developmental period. *BnaC03g65980D* encoded *FAE1* and was involved in FA elongation, showing a rapid increase in expression throughout the process of embryonic development and a significantly higher level in N53-2 than in KenC-8. Of interest, the relative expression level of *BnaC05g44510D* located in *cqOC-C5-7* and involved in cuticular wax synthesis was clearly higher in N53-2 than in KenC-8 but decreased rapidly and was lower in N53-2 during the late embryonic development period. This phenomenon might be associated with the usage of more precursors for the synthesis of TAG in N53-2, especially during late embryonic development.

### Genetic dissection based on the metabolic pathway and interaction network using potential candidates

**Figure 5:**
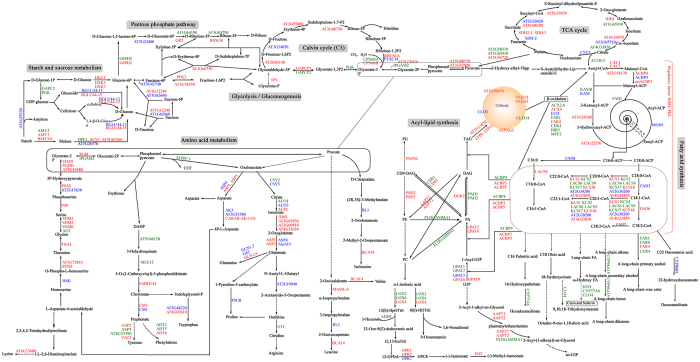
Metabolic pathway based on potential candidates underlying SOC- and SPC-QTLs. The metabolic pathway that was constructed based on potential candidates within QTL regions of SOC and SPC. Red letters indicate orthologues underlying the SOC-QTL regions, blue letters indicate orthologues underlying the SPC-QTL regions, and green letters indicate that these orthologues could be identified in the genomic regions of both SOC- and SPC-QTLs. The broken arrows indicate that there are many steps between the two compounds.

**Figure 6:**
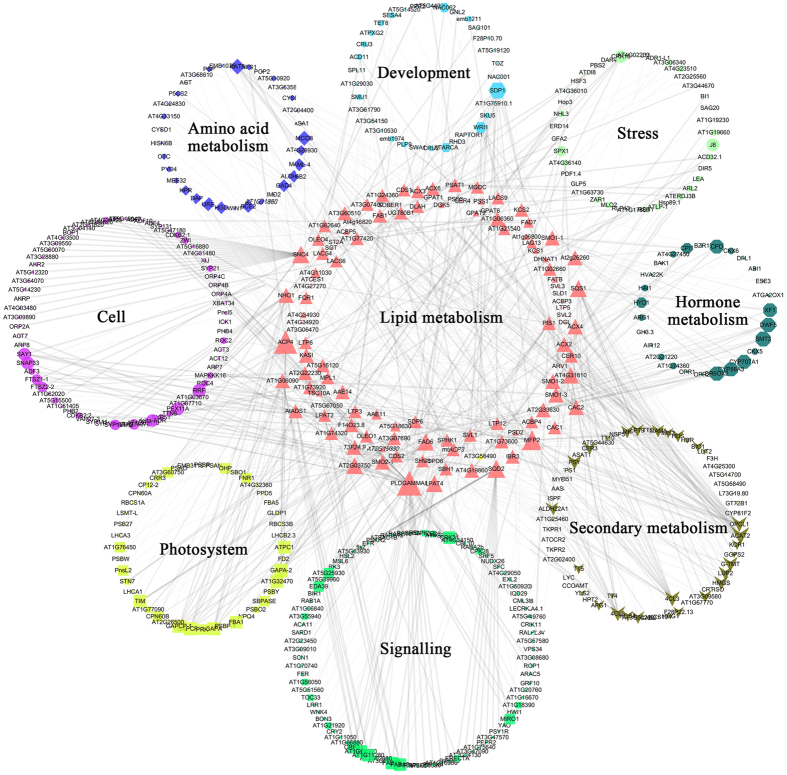
Interaction network associated with lipid metabolism of potential candidates underlying SOC-QTLs. Genes associated with special types of metabolisms and pathways are shown using special shapes with special colors.

To investigate the genetic basis of oil and protein accumulation in the seeds of *B. napus*, a magnificent metabolic pathway was constructed with pyruvate as the centric junction based on potential candidates underlying SOC and SPC-QTLs (Fig. 5). The pathway was involved in almost all primary carbohydrate metabolism, including starch and sucrose metabolism, the pentose phosphate pathway, glycolysis/gluconeogenesis, the Calvin cycle, the TCA cycle, FA synthesis, acyl-lipid metabolism, cutin, suberin and wax biosynthesis and amino acid metabolism. There were 167 and 98 orthologues identified in the SOC- and SPC-QTL regions, respectively, which were involved in this enormous pathway, 58 of which could be identified in both traits. *IPGAM2*, which encodes the 2,3-bisphosphoglycerate-independent phosphoglycerate mutase that catalyzes the formation of glycerate-2P^40^, was identified in overlapping CIs of *qOC-C5-11* for SOC and the QTL *qPC-C5-5* for SPC. During glycolysis/gluconeogenesis, some orthologues of key rate-limiting enzymes, such as *HKL, PFK, GAPCP* and *PGK*, which are involved in a series of catalyzing the conversion of glucose into pyruvate, were candidates in the co-localized CIs or major QTLs. The Calvin cycle carries out carbon fixation and is an important process for photosynthesis. The TCA cycle is of central importance to many biochemical pathways, and it provides the precursors of certain amino acids and the NADH used in numerous other biochemical reactions through the oxidation of acetyl-CoA derived from carbohydrates, oil and proteins. More than half of the orthologues involved in these two key metabolic pathways could be detected in SOC- and SPC-QTL regions. These results suggested that primary carbohydrate metabolism indeed plays a basic role of providing the initial material for protein synthesis and oil accumulation during embryonic development. To further dissect the mechanism of oil accumulation in seeds, the interaction of potential candidates from SOC-QTL regions was analyzed based on *A. thaliana* orthologues. The primary results revealed that the whole network incorporated 5817 nodes and 38887 edges (Figure S7). Annotation and clustering elicited an interaction network associated with lipid metabolism (Fig. 6). Some biologic processes, such as protein, secondary metabolism, photosystems, amino acid metabolism, cell size or cycle, and signaling pathways, participated in the regulation of lipid metabolism through some pivotal genes, such as CAC2, SNC4, ACX2 and *PLDGAMMA1*, which could be expressed during the embryo stage of the plant^41^.

## Discussion

Most studies investigating important agronomic traits in *B. napus* were conducted with lower-density genetic maps^6^,^7^,^8^,^9^,^10^,^11^ and some with high-density linkage maps^25^,^26^,^29^, but QTL analyses of SOC and SPC simultaneously based on high-density linkage maps have not been reported. In this study, we constructed a high-density integrated genetic linkage map with 3106 SNP and 101 non-SNP (SSR and STS) markers covering 3072.7 cM of the KNDH population. The release of the *B. napus* genome and the application of SNP markers with known sequence information facilitated alignment of the genetic linkage and physical maps of *B. napus*. Thus, a high-density SNP map was very helpful for the characterization and comparison of QTLs that control important agronomic traits, and the identification of candidate genes and genomic research. The present study allowed us to obtain a high-density KN linkage map that enabled us to localize QTLs more effectively and accurately. Wang *et al*.^39^ mapped 24 consensus QTLs associated with SOC integrated from 63 identified QTLs in eight microenvironments using the KNDH population from a previous study^39^. Here, 67 SOC-QTLs integrated from 164 identified QTLs were mapped, and ten new SOC-QTLs were contributed due to 4 new microenvironments (Table 2). The main cause of the increased QTL number detected in the present map was the higher map density rather than the addition of new environmental data (the number of consensus QTLs increased from 24 to 67, only 10 of which were contributed by new environmental data).

Molecular-assisted breeding is the primary objective for detecting genetic variation in important agronomic traits. This will help us to identify stable QTLs for molecular-assisted selection (MAS) to compare previously published QTLs for oil content in other segregating populations (with different genetic backgrounds) in our current study. This method has been reported in many studies^42^,^43^. To validate the consistency and availability of the QTLs detected using the present high-density map for MAS, we compared SOC-QTLs with the published QTL map for oil content in other segregating populations with different genetic backgrounds. Twenty-six SOC-QTLs were found to overlap with loci checked by other populations (Table S12). The major QTL, *cqOC-A9-9*, was also detected and overlapped with *qOC-A9-4-TN* in the TN population, which was used to identify the seed-oil content and erucic acid content QTLs^44^, and the QTLs for many other important agronomic traits^45^,^46^,^47^. Additionally, *qOC-A3-4*, which could be stably expressed in 8 microenvironments, could also be identified to the co-localized locus in the TN population. These QTLs are very stable and useful for further MAS analyses. It is interesting that some QTLs detected in other populations were identified and divided into multiple QTLs. For example, *qOC-A8-1-TN* and *qOC-A8-RNSL* from TN and RNSL, respectively, were overlapping, and both of them were found to cover 5 QTLs in the present study. This result also suggested that the present genetic linkage map had a better resolution than the maps used in previous reports.

The recently proposed NGS-based BSA approach is a cost-effective and rapid method of trait mapping supported by the identification of the target genomic region in rice^33^. A large number of AGRs were identified rapidly, and significant AGRs with a high Δ (SNP-index) were consistent with the SOC-QTL hotspots, confirming the accuracy of the QTL mapping based on the present map. In addition, we found that highly significant AGRs were subdivided into multiple QTLs (Table S6) rather than being subdivided or narrowed QTLs as previously reported^34^,^35^,^48^,^49^. These results suggested that the present high-density linkage map had sufficiently high resolution to dissect genetic variation in primary mapping populations.

The application of a high-density linkage map and satisfying linear relationship between the genetic and physical map in our study could be helpful to more accurately identify candidate genes underlying SOC and SPC. A large number of potential candidates involved in acyl-lipid pathways and seed storage were identified within SOC and SPC-QTL regions, respectively (Tables S10 and S11). Additionally, for the most important candidates of SOC, a whole genome search was conducted, followed by a search for enrichment/prevalence in the QTL confidence intervals compared to the whole genome. Minor acyl-lipid related gene enrichment/prevalence was found in the CIs of SOC-QTLs, which indicated that the important genes clustered around QTLs and were consistent with the frequently identified QTL hotspots in the present study. Six potential candidates related to acyl-lipid metabolism were selected as representatives to validate their expression levels between the two parents. The relative differences in the expression of five genes showed a relationship with the SOC phenotype of the two parents in developing seed. The results provided clues for exploring the casual genes underlying SOC. Single gene mutations may affect the expression of some genes in the common metabolic network. Consequently, further identification of the network is necessary, and simultaneously the rapid capture of causal genes requires the use of transcription and fine mapping methods^50^.

In the present study, the simultaneous identification of QTLs for SPC together with SOC using the high-density linkage map revealed that some QTLs affected both SOC and SPC in an opposite manner (Table 3). SOC and SPC, which have a negative correlation, are controlled by many genes with additive and epistatic effects^19^,^23^,^51^,^52^. Oil and protein are synthesized in the endoplasmic reticulum (ER) during the same period of seed development^53^, which suggests that these pathways interfere and/or compete with one another for ER resources. TAG synthesis competes with seed storage protein synthesis due to sucrose utilization, and the reduction of one product would result in higher levels of the other. Pyruvate, which can be converted into carbohydrates via gluconeogenesis, to FAs or energy through acetyl-CoA and amino acids, is a key intersection in the network of metabolic pathways and unites several key metabolic processes. Here, we constructed a magnificent primary carbohydrate pathway in the center of pyruvate using potential candidates underlying SOC- and SPC-QTLs (Fig. 5). *A. thaliana* orthologues involved in this pathway provide more information to identify the interference and/or competition mechanism from a metabolic perspective. In addition, some genes associated with cuticular waxes, cutin and suberin have also been observed, such as *CER4, CYP86, ACC1* and *MAH1*, and they probably affect seed storage triacylglycerides through competition with the common fatty acid pool^54^,^55^. Interestingly, most orthologous genes involved in long chain fatty acid synthesis, especially C20:X and C22:X, fell within SOC-QTL regions. These results were in agreement with the findings of Gu *et al*.^56^, in which long chain FAs had a significant and positive relationship with oil content.

Oil and protein biosynthesis during embryo development is a complex and hierarchical biological process that is regulated by some transcription factors and complex gene interaction networks^57^,^58^. Therefore, it is not inadequate to dissect the mechanism responsible for oil and protein accumulation in seeds only through primary carbohydrate metabolic pathways. In *A. thaliana*, metabolic pathways associated with the biosynthesis and degradation of acyl-lipids have been summarized, and most of the genes have been described^38^. The construction of an interaction network provided new insights into the interaction among related genes (Fig. 6). It has been reported that the yellow seeded *Brassica* genotypes contained greater oil, higher protein and lower fiber contents than the seeds of the black/brown seeded genotypes^59^. In plants, flavonoids are the major secondary metabolites involved in the *Brassica* seed coat pigmentation process^60^. The orthologues *TT4* and *TT5*, which were classified into secondary metabolism pathways, were involved in flavonoid biosynthetic processes and regulated lipid metabolic pathways by interacting with *CAC2* (Fig. 6). In addition, the result verified by the cross-talk between the lipids and potential flavonoid metabolism were driven by the finding that acetyl-CoA acts as a substrate for both pathways^61^. In addition, *4CL* family proteins associated with lignin biosynthesis are linked to lipid metabolism through many nodes, which could help to explain the association with negative additive effects of QTLs for seed oil and lignin content (unpublished data). These results not only enhance our understanding of the complex regulatory mechanisms of oil accumulation but also serve as a basis for follow-up to identify causal loci together with transcriptome and genome-wide association analyses^62^,^63^,^64^.

## Materials and Methods

### Plant material and trait measurement

The segregating double haploid (DH) population, named KN, was used in this experiment. The KNDH population was derived from a cross between the parental lines KenC-8 with low oil content and N53–2 with high oil content, which was first constructed by Wang *et al*.^39^. A total of 300 lines of the KNDH population were implemented in the genotype and phenotype analyses in this study.

The field experiments were conducted in 3 macroenvironments as described by Wang *et al*.^39^: Dali of Shaanxi Province (DL), Wuhan and Huanggang of Hubei Province (WH and HG) and Sunan of Gansu Province (GS), which were winter, semi winter and spring-type rapeseed planting areas. Year–location combinations were treated as microenvironments. The materials were planted in Dali, Wuhan, Sunan and Huanggang for, successively, six, three, two and one year, respectively. Four types of microenvironmental data were added in this experiment apart based on a report by Wang *et al*.^39^. In total, there were 12 microenvironments. The SOC data from 2012 and 2013 in DL and WH, excluding the report by Wang *et al*.^39^, were collected. The SPC data were collected in all microenvironments except 08DL. The trial locations of WH and HG were the experiment bases of Huazhong University of Science and Technology (Wuhan), and DL and GS were the experiment bases of the Hybrid Rapeseed Research Center of Shaanxi Province. No specific permissions were required for the field trials. The field experiment was the same as reported by Wang *et al*.^39^.

Open-pollinated seeds were collected for the SOC and SPC measurements. The SOC data in the first eight trials (08DL, 09DL, 10DL, 10HG, 10GS, 11DL, 11GS, 11WH) were measured by nuclear magnetic resonance (NMR) according to Burns *et al*. (2003) with modifications^6^. The SOC data for the other four trials and all SPC data were measured by near-infrared reflectance spectroscopy as described by Gan *et al*.^65^.

### SNP marker analysis

Whole-genomic DNA was extracted from young seedlings using the CTAB method^66^. A total of 300 DH lines with two parental lines were successfully genotyped using the Brassica 60 K Infinium HD Assay SNP arrays according to the manufacturer’s protocol provided by Illumina Inc. (http://www.illumina.com/). Imaging of the arrays after BeadChip washing and coating was performed using an Illumina HiSCAN scanner. Allele calling data for each probe was exported using Genome Studio genotyping software v2011 (Illumina Inc.). SNP data filtering was accomplished according to Zhang *et al*.^26^. Furthermore, SNPs with identical genotypes across the KNDH population were classified into a bin using Perl language based on a close linkage according to Zhang *et al*.^26^, and the first SNP of each bin was selected as its name.

### Linkage map construction, QTL analysis and integration

The construction of genetic linkage groups, QTL analysis and integration were performed as described by Wang *et al*.^39^. Non-SNP (SSR, STS, and SRAP) markers were used to construct the primary linkage map used by Wang *et al*.^39^ and also applied to construction of the map, together with the SNP-bin markers. Carthagene software was used to construct the high-density linkage map^67^. Windows QTL Cartographer 2.5 software was used to detect putative QTLs with a CIM^68^. For the CIM model, the scan walking speed was 2 cM, and the window size was 10 cM with five background cofactors. The LOD threshold (3.2–3.5) for the detection of significant QTLs was calculated by a 1,000-permutation test based on a 5% experiment-wise error rate. To avoid missing QTLs with small genetic effects, QTLs that appeared repeatedly in at least two microenvironments, below the LOD threshold (3.2–3.5) but above 2.0, were considered micro-real QTLs according to the definition of Long *et al*.^46^. All these QTLs, including significant and micro-real ones, were called identified QTLs^69^. The identified QTLs were named by combining the microenvironment and the chromosome number, e.g., *qOC-10DL13-1*. The identified QTLs detected in different trials with overlapping CIs were then integrated into consensus QTLs using the BioMercator V4.2 program^70^. If an identified QTL had no overlapping CIs with others, then it was also regarded as a consensus QTL. Consensus QTLs were designated by the initial letters “cq”, such as *cqOC-A1-1*. The consensus QTLs with overlapping CIs for SOC and SPC were integrated into unique QTLs, and unique QTLs were named by the initial letter “uq”, for example, *uq*-*A9-1*. The method used for QTL nomenclature and integration were as described by McCouch *et al*.^71^ and Wang *et al*.^72^.

### Comparative analysis of the genetic map and physical map, and the identification of genomic regions corresponding to CIs of QTLs and potential candidates underlying QTLs

The method used to identify the homologous locus of SNP probes in *B. napus* reference genomes was described according to Cai *et al*.^73^. The sequences of the 50-mer probes provided by Illumina Inc. were used as queries to search for homologous loci using the NCBI-Blastn local program against the *B. napus* “Darmor-bzh” reference genomes (http://www.genoscope.cns.fr/brassicanapus/data), as described by Altschul *et al*.^74^. The E-value threshold was set to 1e-10. All SNPs assigned to the linkage map were used to validate the alignment relationship of the genetic map and physical map. If an SNP was mapped to multiple loci in the *B. napus* reference genome, only the location that corresponded to the particular linkage group of the locus was selected^30^. Additionally, if a SNP had multiple homologous loci on the same chromosome, an accurate position for a particular locus was determined manually by referring to the physical positions of its upstream and downstream SNPs. Genome regions corresponding to the CIs of consensus QTLs, herein denoted QTL regions, were defined through both closely linked SNPs within QTL CIs and co-linearity between the linkage map and the reference genome. Genes that fell within QTL regions were regarded as candidates. According to the BSA re-sequencing, alleles that were within QTL regions and had SNP or InDel variations in introns, exons or within 1 kb up- and downstream between two parents were considered potential candidates, and otherwise were excluded.

The genomic regions of QTLs from other populations, DY (‘Darmor-bzh’ × ‘Yudal’, RNSL (‘Rapid’ × ‘NSL96/25’)^75^, Z5 (‘zy036’ × ‘51,070’)^76^, SG (‘Sollux’ × ‘Gaoyou’)^77^, TN (‘Tapidor’ × ‘Ningyou7’)^42^, SO (‘Sansibar’ × ‘Oase’)^78^, PT (‘Polo’ × ‘Topas’)^79^, and M201 × M202^80^, were obtained via electronic PCR (e-PCR)^81^, which was performed with the primer sequences of the molecular markers flanking the QTL confidence intervals using the genomic sequences of ‘Darmor-*bzh*’ as templates.

### BSA sequencing and AGR analysis

DNA was extracted from individuals in the KNDH population with extremely high and low SOC (24 DNA samples for each extreme trait) and bulked equally to generate high and low extreme bulks. Four libraries, including two parents (KenC-8 and N53-2), were constructed and then sequenced using the Illumina HiSeq2500 platform at the Novogene Bioinformatics Institute, and 125-bp paired-end reads were generated with an insert size of approximately 350 bp. Short reads obtained from both parents and two DNA bulks were aligned against the *B. napus* “Darmor-bzh” reference genomes to obtain the consensus sequence using BWA software^82^. Reads of high and low SOC bulks were separately aligned to the KenC-8 (low SOC parent) consensus sequence reads to call the SNPs. Variant calling was performed for all samples using the Unified Genotyper function in the GATK software^83^. SNPs were used in the Variant Filtration parameter in GATK. Homozygous SNPs between two parents were extracted, and the read depth information for homozygous SNPs in the above bulks was acquired to calculate the SNP Index^33^. The Δ (SNP-index) was calculated based on subtraction of the low SOC bulk from the high SOC bulk SNP-index. The average SNP-index of SNPs in a certain genomic interval was calculated using a sliding window analysis with a 1-Mb window size and 10-kb step size, and the SNP-index graphs and corresponding Δ (SNP-index) graphs were plotted. The genomic regions with a significant Δ (SNP-index) (P < 0.05) were identified as AGRs.

### Quantitative PCR (qPCR) analysis of 6 acyl-lipid-related potential candidates

To investigate the potential candidates, we used qRT-PCR for six potential candidates related to acyl-lipids from different associated loci. RNAs were extracted and quantified from developing *B. napus* seeds at 15, 25 and 35 days after flowering (DAF), following the user manual for the RNA prep Pure Plant Kit (TOYOBO, DP441). cDNA was synthesized from 2 mg of total RNA using a cDNA Synthesis Kit. Expression analysis was performed with the SYBR premix EX TaqTM kit (TaKaRa, Japan) using an ABI 7900HT Fast Real-Time PCR System (Applied Biosystems, Framingham, USA). For each reaction, three technical replicates were evaluated. The primers of the six target genes and reference gene Actin are listed in Table S13.

### The metabolic network and interaction analysis of potential candidates

The orthologues of potential candidates in QTL regions and their annotation were obtained by BLASTn based on the *A. thaliana* database (http://www.arabidopsis.org/). The same method was used for the whole genome search of acyl-lipid-related orthologues in *B. napus*. Potential candidates were clustered and analyzed using Mapman software^84^ according to their orthologues and annotation. The orthologues involved in acyl-lipid metabolism were acquired from the Acyl-Lipid Metabolism chapter from The Arabidopsis Book^38^ and the ARALIP website (http://aralip.plantbiology.msu.edu/). The interaction was analyzed and constructed using the online software String (http://string-db.org/), clustered again using Mapman software and finally visualized using Cytoscape_V3.2.1^85^. All the interaction and cluster and analyses were based on *A. thaliana* orthologues.

## Additional Information

**How to cite this article:** Chao, H. *et al*. Genetic dissection of seed oil and protein content and identification of networks associated with oil content in *Brassica napus. Sci. Rep.* **7**, 46295; doi: 10.1038/srep46295 (2017).

**Publisher's note:** Springer Nature remains neutral with regard to jurisdictional claims in published maps and institutional affiliations.

## Supplementary Material

Supplementary Figures and Tables

## Figures and Tables

**Table 1:** Characteristics of the KNDH linkage map based on SNP-bins and non-SNP markers.

Linkage group	SNP-bins (No.)	Non-SNP (No.)	Marker (No.)	Length (cM)	SNP-bins (%)	Average distance between adjacent loci (cM)
A01	168	10	178	177.1	94.38	0.99
A02	174	12	186	199	93.55	1.07
A03	321	3	324	218.4	99.07	0.67
A04	156	0	156	86.8	100	0.56
A05	134	0	134	80.1	100	0.60
A06	211	13	224	178.6	94.2	0.80
A07	202	0	202	149.1	100	0.74
A08	77	0	77	92	100	1.19
A09	175	0	175	123.5	100	0.70
A10	130	7	137	94	94.89	0.69
C01	172	3	175	182.2	98.28	1.04
C02	134	0	134	132.4	100	0.99
C03	278	13	291	337.1	95.53	1.16
C04	156	4	160	220.6	97.5	1.38
C05	172	7	179	263.2	96.09	1.47
C06	172	12	184	226.3	93.48	1.23
C07	94	6	100	117	94	1.17
C08	95	5	100	103.9	95	1.04
C09	85	6	91	91.4	93.41	1.00
A genome	1748	45	1793	1398.6	97.49	0.78
C genome	1358	56	1414	1674.1	96.04	1.18
Total (A + C)	3106	101	3207	3072.7	96.85	0.96

**Table 2:** Consensus QTLs for SOC and SPC identified by a high-density linkage map of the KNDH population.

Trait	Consensus QTL	Chromosome	Peak	Range	LOD	Additive	PV (%)	Environment
**SOC**	***cqOC-A1***	A01	109.41	104.00–112.10	4.32047	0.7173	9.59	*08DL*
	***cqOC-A2–1***	A02	2.30	0–7.08	2.06–2.34	−0.41– −0.43	1.97–2.22	*10DL/11DL*
	***cqOC-A2-2***	A02	107.71	105.80–109.20	7.69916	−1.2	14.18	*11GS*
	***cqOC-A2–3***	A02	116.26	113.42–119.10	2.30–7.35	−0.66– −1.16	3.43–13.72	*11GS/10GS/13WH*
	***cqOC-A3-1***	A03	26.01	23.58–28.43	2.13–3.37	−0.52– −0.68	4.36–6.00	*10GS/11GS*
	***cqOC-A3-2***	A03	32.51	31.9–35.8	3.38086	−0.68	6.02	*11GS*
	***cqOC-A3-3***	A03	122.37	120.45–124.29	2.28–3.50	0.35–0.50	2.12–3.13	*09DL/11DL/12DL*
	***cqOC-A3-4***	A03	151.81	150.70–153.90	3.65862	0.5785	3.44	*11DL*
	***cqOC-A3-5***	A03	182.85	182.10–183.59	2.14–2.45	0.37–0.49	1.94–2.41	*11DL/12DL*
	***cqOC-A3-6***	A03	191.71	183.20–195.70	3.71721	0.6261	4.12	*11DL*
	***cqOC-A3-7***	A03	204.14	200.26–208.03	2.14–3.59	0.38–0.73	2.08–3.76	*11DL/12DL/13WH/12DL*
	***cqOC-A8-1***	A08	1.47	1.08–1.85	2.40–3.48	0.40–0.50	2.16–3.25	*12DL/10DL*
	***cqOC-A8-2***	A08	8.61	7.34–9.88	2.331–5.32	0.38–0.62	2.45–5.32	*10GS/09DL/13DL/10DL/12DL*
	***cqOC-A8-3***	A08	12.32	10.85–13.78	2.60–3.72	0.58–0.72	3.50–6.82	*11GS/11DL/10GS*
	***cqOC-A8-4***	A08	18.01	17.61–18.41	3.45–5.12	0.47–0.85	3.09–9.36	*12DL/10DL/11GS/13DL*
	***cqOC-A8-5***	A08	20.01	19.53–20.49	2.84–4.83	0.37–0.67	2.62–5.89	*10HG/09DL*
	***cqOC-A8-6***	A08	21.91	20.9–23.2	3.19–5.34	0.59–0.86	3.90–9.76	*12WH/11GS*
	***cqOC-A9-1***	A09	1.17	0.19–2.14	2.04–2.69	0.36–0.47	2.14–2.52	*10DL/09DL/10HG*
	***cqOC-A9–2***	A09	23.27	21.81–24.73	2.19–4.38	0.42–0.57	2.54–4.18	*09DL/10DL/10HG/13DL*
	***cqOC-A9-3***	A09	31.21	30.4–32.2	4.2098	0.532	3.88	*13DL*
	***cqOC-A9-4***	A09	34.26	32.36–36.17	2.45–5.35	0.41–0.72	2.30–5.40	*11DL/10DL/09DL*
	***cqOC-A9-5***	A09	67.61	67.1–73.1	3.22245	−0.56	3.04	*11DL*
	***cqOC-A9-6***	A09	95.11	94.9–96.7	4.50709	0.5957	4.82	*12DL*
	***cqOC-A9-7***	A09	98.41	97.23–99.58	2.96–4.80	0.49–0.68	3.06–5.14	*10DL/12WH*
	***cqOC-A9-8***	A09	100.71	99.9–101.2	9.18561	0.8578	10.05	*13DL*
	***cqOC-A9-9***	A09	106.28	105.79–106.77	2.76–14.19	0.64–1.05	4.88–14.94	*09DL/11GS/13DL/12DL/12WH/10GS*
	***cqOC-A9-10***	A09	112.7	111.62–113.79	2.87–11.48	0.69–0.81	3.32–12.41	*09DL/11DL/12WH/13WH*
	***cqOC-A9-11***	A09	116.38	116.16–116.59	7.33–7.66	0.64–0.79	7.76–8.46	*13DL/09DL*
	***cqOC-A9-12***	A09	122.01	119.88–124.13	2.60–7.21	0.66–0.89	7.25–8.05	*10GS/11DL*
	***cqOC-A10-1***	A10	1.11	0–2.59	3.64–3.79	−0.61– −0.64	3.39–4.56	*10DL/09DL*
	***cqOC-A10-2***	A10	8.71	5.8–16.7	2.80147	−0.68	2.73	*11DL*
	***cqOC-A10-3***	A10	18.03	14.35–21.71	2.12–3.13	−0.56– −1.02	2.00–4.43	*11WH/10DL/12DL*
	***cqOC-C2-1***	C02	84.14	82.1–86.18	2.60–3.66	0.47–0.82	2.44–8.68	*10DL/10GS*
	***cqOC-C2–2***	C02	91.10	89.17–93.04	2.95–3.13	0.49–0.76	2.76–7.48	*10DL/10GS*
	***cqOC-C2-3***	C02	100.71	97.6–103.1	3.69768	0.5657	3.90	*10DL*
	***cqOC-C2-4***	C02	111.08	109.45–112.72	3.72–2.10	0.33–0.53	1.95–3.50	*10DL/09DL*
	***cqOC-C3-1***	C03	144.94	142.23–147.64	2.26–2.74	0.37–0.4417	2.56–2.20	*12WH/09DL*
	***cqOC-C3–2***	C03	166.11	163.56–168.65	2.35–5.26	0.48–0.90	2.24–12.72	*10GS/11DL*
	***cqOC-C3-3***	C03	172.61	171.6–173.9	4.08611	0.8218	10.25	*10GS*
	***cqOC-C3–4***	C03	300.48	299.84–301.12	2.69–8.04	0.45–1.052	2.52–8.12	*11WH/10HG/12DL/09DL/10DL/11DL/13DL/13WH*
	***cqOC-C3-5***	C03	309.01	308.17–309.86	3.08–8.01	0.56–0.78	3.84–8.03	*10HG/09DL/13DL*
	***cqOC-C3-6***	C03	312.33	311.35–313.31	3.44–7.86	0.51–1.20	3.21–9.32	*10DL/13WH/12DL/12WH*
	***cqOC-C3-7***	C03	318.41	314.17–322.64	4.59–6.23	0.60–1.09	4.92–7.86	*12DL/13DL/13WH*
	***cqOC-C5-1***	C05	71.99	58.2–85.8	5.66153	0.9311	10.86	*10HG*
	***cqOC-C5-2***	C05	135.39	123.4–136	3.82137	−0.5302	3.57	*10DL*
	***cqOC-C5-3***	C05	183.19	181.38–184.99	2.35–10.20	0.45–1.00	2.86–10.71	*09DL/11DL/11WH*
	***cqOC-C5-4***	C05	191.08	190.1–192.06	2.17–10.64	0.37–1.02	2.09–11.11	*11DL/09DL/12WH*
	***cqOC-C5-5***	C05	220.41	219.85–220.98	3.83–7.55	0.47–0.77	3.90–7.64	*09DL/10DL/11DL/12DL/12WH*
	***cqOC-C5-6***	C05	230.24	229.22–231.26	2.42–7.33	0.54–0.76	2.3–7.43	*12WH/09DL/10DL/12DL*
	***cqOC-C5-7***	C05	234.02	232.79–235.24	2.42–7.33	0.50–0.75	2.36–7.43	*10DL/11DL*
	***cqOC-C5-8***	C05	237.39	234.66–240.11	3.46–4.34	0.53–0.89	3.87–5.64	*13DL/13WH*
	***cqOC-C5-9***	C05	250.78	249.56–252.01	4.02–4.37	0.53–0.85	3.91–5.03	*13DL/13WH*
	***cqOC-C5-10***	C05	258.99	257.20–259.40	4.20546	0.9034	5.68	*11WH*
	***cqOC-C6-1***	C06	23.98	15.25–32.72	2.66–3.26	0.47–0.85	2.80–5.03	*11WH/13DL*
	***cqOC-C6-2***	C06	149.34	147.65–151.02	2.29–4.44	0.50–0.83	2.79–4.32	*10HG/13DL/13WH*
	***cqOC-C6-3***	C06	159.20	152.6–162.4	2.03–3.72	0.73–1.48	3.01–3.67	*13DL/12DL/13WH*
	***cqOC-C6-4***	C06	160.61	155.2–169.8	3.61–4.08	0.60–0.68	3.73–4.53	*10HG/13DL*
	***cqOC-C6-5***	C06	181.81	180.18–183.45	2.98–4.35	0.52–0.75	2.75–4.58	*13DL/10DL/11DL*
	***cqOC-C6-6***	C06	188.71	187.58–188.96	2.39–13.53	0.37–4.85	3.78–22.24	*12DL/10GS/09DL/10DL/11DL/13DL*
	***cqOC-C6-7***	C06	195.61	195.2–197	5.41	0.80	5.65	*11DL*
	***cqOC-C6-8***	C06	199.11	198.88–199.33	5.972–6.45	−2.052–2.12	6.17–6.24	*10DL/12DL*
	***cqOC-C6-9***	C06	201.31	200.4–202.3	3.98846	0.5657	3.64	*13DL*
	***cqOC-C6-10***	C06	203.8	203.56–204.05	3.62–4.98	−1.99–2.06	3.42–4.88	*11DL/12DL*
	***cqOC-C6-11***	C06	207.51	206.5–211.6	3.46766	0.5242	3.18	*13DL*
	***cqOC-C8***	C08	66.15	62.66–69.65	2.03–3.75	0.40–0.79	1.92–9.55	*10GS/10DL*
	***cqOC-C9-1***	C09	7.39	4.08–10.7	2.01–6.41	0.45–0.68	1.98–6.49	*12WH/10DL/11DL/12DL/09DL*
	***cqOC-C9-2***	C09	27.11	24.6–29.8	3.80835	0.5388	3.70	*13DL*
**SPC**	***cqPC-A2***	A02	0.01	0–1.13	2.91–4.83	0.25–0.47	3.42–10.09	11GS/12DL
	***cqPC-A3-1***	A03	127.01	124.80–127.70	3.46	−0.34	4.23	11WH
	***cqPC-A3-2***	A03	131.81	131.30–132.30	8.7	−2.12	27.48	10GS
	***cqPC-A3-3***	A03	135.51	133.00–139.90	4.64	−0.4	5.68	11WH
	***cqPC-A3-4***	A03	203.51	200.58–206.43	2.11–7.24	−0.22–0.58	2.65–7.75	12DL/13DL/13WH
	***cqPC-A3-5***	A03	216.61	214.60–216.90	6.64	1.95	20.53	10GS
	***cqPC-A4-1***	A04	14.59	13.00–15.50	3.74	−0.44	7.65	11GS
	***cqPC-A4-2***	A04	55.29	52.50–56.40	4.7	−0.53	6.19	12WH
	***cqPC-A7***	A07	97.71	95.80–100.90	4	−1.01	4.89	09DL
	***cqPC-A9-1***	A09	1.17	0–5.36	2.10–4.42	−0.27–0.32	2.53–5.23	10DL/12DL
	***cqPC-A9-2***	A09	16.4	13.47–19.32	2.93–4.15	−0.31–0.53	3.82–5.39	12WH/10HG/11DL
	***cqPC-A9-3***	A09	23.89	20.59–27.18	2.14–4.20	−0.26–0.53	2.57–5.44	10DL/11DL
	***cqPC-A9-4***	A09	32.01	31.20–33.40	4.42	−0.38	5.34	11WH
	***cqPC-A9-5***	A09	37.31	33.4–42.21	3.04–4.42	−0.22–0.38	3.01–5.34	11WH/13DL
	***cqPC-C1-1***	C01	93.79	93.05–94.53	2.20–4.58	−0.19–0.39	2.18–5.86	13DL/10DL
	***cqPC-C1-2***	C01	152.19	150.05–154.33	4.02–8.22	0.38–1.66	5.20–10.36	10HG/09DL/13WH
	***cqPC-C1-3***	C01	159.36	158.0 0−160.72	7.80–4.88	0.96–1.59	6.26–9.86	09DL/13WH
	***cqPC-C1-4***	C01	168.01	166.00–176.90	3.67	0.85	4.78	13WH
	***cqPC-C3-1***	C03	287.81	287.50–288.40	3.44	−0.35	4.48	11WH
	***cqPC-C3-2***	C03	298.77	297.38–300.15	7.08–8.27	−0.39–0.49	8.74–8.92	11WH/13DL
	***cqPC-C3-3***	C03	303.72	302.84–304.59	3.767–8.02	−0.35–0.53	4.82–9.82	10HG/11WH
	***cqPC-C3-4***	C03	312.51	310.1–319.9	3.47	−0.34	4.47	10HG
	***cqPC-C5-1***	C05	133.89	121.3–136	4.42	1.37	5.61	10DL
	***cqPC-C5-2***	C05	141.96	139.89–144.03	4.22–5.33	−0.25–0.32	5.42–6.64	10DL/09DL
	***cqPC-C5-3***	C05	225.49	221.80–225.90	5	−0.38	6.55	11DL
	***cqPC-C5-4***	C05	231.59	231.20–233.90	3.63	−0.41	4.78	11DL
	***cqPC-C5-5***	C05	237.39	234.67–240.1	2.48–4.50	−0.29–0.6	5.86–3.34	10DL/12DL/13DL
	***cqPC-C5-6***	C05	252.21	251.54–252.89	2.20–7.91	−0.24–0.88	2.91–8.51	10DL/12DL/13WH/13DL
	***cqPC-C5-7***	C05	260.81	260.26–261.37	2.68–5.72	0.38–1.7758	3.41–6.25	12DL/13DL/13WH
	***cqPC-C6-1***	C06	54.45	50.31–58.58	2.08–3.17	−0.27–0.29	2.39–4.03	12DL/11WH
	***cqPC-C6-2***	C06	149.41	145.01–153.8	2.53–4.39	−0.24–0.42	2.87–5.59	10DL/12DL
	***cqPC-C6-3***	C06	156.6	153.85–159.35	2.28–3.24	−0.34–0.37	4.31–5.84	10GS/10DL
	***cqPC-C7***	C07	109.84	105.01–114.66	2.21–4.77	−0.28–0.32	2.78–5.68	12DL/11WH
	***cqPC-C8***	C08	15.39	11.3–16.7	4.41	0.28	4.62	13DL
	***cqPC-C9-1***	C09	2	0.0–6.7	2.23	−0.28–0.35	2.86–4.45	12WH/10DL
	***cqPC-C9-2***	C09	8.12	6.47–9.78	2.055–2.98	−0.32–0.34	3.88–4.18	12WH/11GS
	***cqPC-C9-3***	C09	70.54	69.35–71.72	2.31–3.09	−0.23–0.603	2.92–3.22	13DL/13WH
	***cqPC-C9-4***	C09	77.31	74.86–79.75	2.41–2.60	−0.2–0.64	2.51–3.28	13DL/13WH

**Table 3:** Unique QTLs controlling both SOC and SPC.

Unique QTL	Peak	Range	Consensus QTL	Peak	Range	LOD	PV (%)	Additive
***uqA3***	203.73	201.4–206.07	***cqOC-A3-7***	204.14	200.26–208.03	2.14–3.59	2.08–3.76	0.38–0.73
			***cqPC-A3-4***	203.51	200.58–206.43	2.11–7.24	2.65–7.75	−0.22–0.58
***uqA9-1***	1.16	0.25–2.08	***cqOC-A9-1***	1.17	0.19–2.14	2.04–2.69	2.14–2.52	0.36–0.47
			***cqPC-A9-1***	1.17	0–5.36	2.10–4.42	2.53–5.23	−0.27–0.32
***uqA9-2***	23.37	22.03–24.7	***cqOC-A9-2***	23.27	21.81–24.73	2.19–4.38	2.54–4.18	0.42–0.57
			***cqPC-A9-3***	23.89	20.59–27.18	2.14–4.20	2.57–5.44	−0.26–0.53
***uqA9-3***	31.53	30.83–32.22	***cqOC-A9-3***	31.21	30.4–32.2	4.21	3.88	0.53
			***cqPC-A9-4***	32.01	31.20–33.40	4.42	5.34	−0.38
***uqA9-4***	34.74	32.99–36.48	***cqOC-A9-4***	34.26	32.36–36.17	2.45–5.35	2.30–5.40	0.41–0.72
			***cqPC-A9-5***	37.31	33.4–42.21	3.04–4.42	3.01–5.34	−0.22–0.38
***uqC3***	312.33	311.37–313.29	***cqOC-C3-6***	312.33	311.35–313.31	3.44–7.86	3.21–9.32	0.51–1.20
			***cqPC-C3-4***	312.51	310.1–319.9	3.47	4.47	−0.34
***uqC5-1***	134.75	129.97–139.53	***cqOC-C5-3***	135.39	123.4–136	3.82	3.57	−0.53
			***cqPC-C5-1***	133.89	121.3–136	4.42	5.61	1.37
***uqC5-2***	237.38	235.46–239.31	***cqOC-C5-11***	237.39	234.66–240.11	3.46–4.34	3.87–5.64	0.53–0.89
			***cqPC-C5-5***	237.39	234.67–240.1	2.48–4.50	3.34–5.86	−0.29–0.6
***uqC6-1***	149.34	147.77–150.92	***cqOC-C6-2***	149.34	147.65–151.02	2.29–4.44	2.79–4.32	0.50–0.83
			***cqPC-C6-2***	149.41	145.01–153.8	2.53–4.39	2.87–5.59	−0.24–0.42
***uqC6-2***	157.55	155.27–159.83	***cqOC-C6-3***	159.2	152.6–162.4	2.03–3.72	3.01–3.67	0.73–1.48
			***cqPC-C6-3***	156.6	153.85–159.35	2.28–3.24	4.31–5.84	−0.34–0.37
***uqC9***	7.97	6.49–9.45	***cqOC-C9-1***	7.39	4.08–10.7	2.01–6.41	1.98–6.49	0.45–0.68
			***cqPC-C9-2***	8.12	6.47–9.78	2.055–2.98	3.88–4.18	−0.32–0.34
