# Supplementary material for: Genetic dissection of seed oil and protein content and identification of networks associated with oil content in Brassica napus
Source: Sci Rep. 2017 Apr 10;7:46295. doi: 10.1038/srep46295 (PMC5385559; doi:10.1038/srep46295)
Supplement: Supplementary Figures and Tables [file srep46295-s1.doc]

**Genetic dissection of seed oil and protein content and identification of networks associated with oil content in *Brassica napus***

Hongbo Chao^1,3,+^, Hao Wang^2,+^, Xiaodong Wang^1,4^, Liangxing Guo^1^, Jianwei Gu^1^, Weiguo Zhao^1,3^, Baojun Li^3^, Dengyan Chen^1^, Nadia Raboanatahiry^1^, Maoteng Li^1,*^

*^1^Department of Biotechnology, College of Life Science and Technology, Huazhong University of Science and Technology, Wuhan, 430074, China*

*^2^Hybrid Rapeseed Research Center of Shaanxi Province, Shaanxi Rapeseed Branch of National Centre for Oil Crops Genetic Improvement, Yangling, 712100, China*

*^3^Hubei Collaborative Innovation Center for the Characteristic Resources Exploitation of Dabie Mountains, Huanggang Normal University, Huanggang, 438000, China*

*^4^Key Laboratory of Cotton and Rapeseed, Ministry of Agriculture, Institute of Industrial Crops, Jiangsu Academy of Agricultural Sciences, Nanjing, 210014, China*

^+^These authors contributed equally to this work

***Correspondence:** limaoteng426@mail.hust.edu.cn


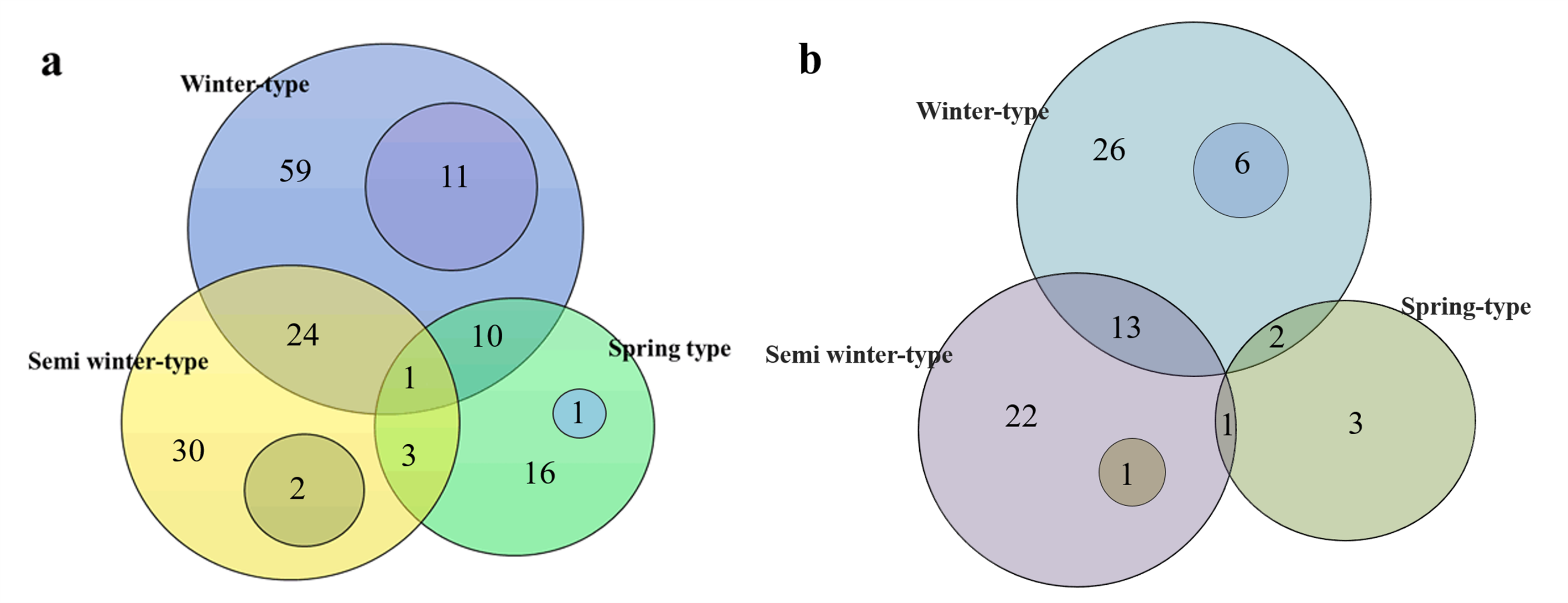


**Figure S1. Distribution of QTLs in different macroenvironments.** The small disc inside each macroenvironment disc indicates that these QTLs could be detected over multiple years for this macroenvironment. (a) Distribution of SOC-QTLs in three macroenvironments. (b) Distribution of SPC-QTLs in three macroenvironments.

**
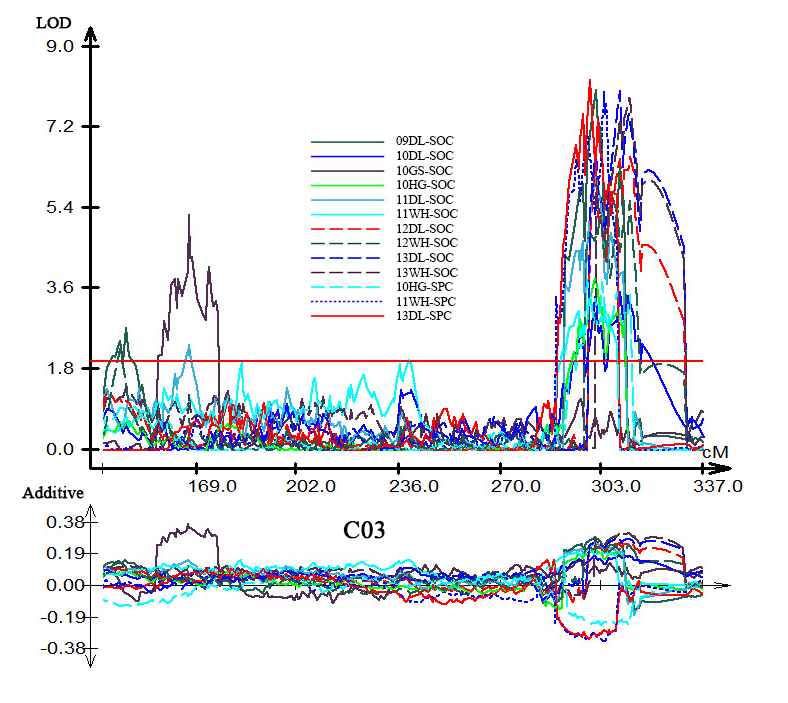
**

**Figure S2.** **QTL hotspots in linkage group C03 affected both SOC and SPC with opposite additive effects.** Original QTL identification in different experiments, as demonstrated by curves above the line of the linkage group (X-axis), and their additive effects are shown by curves with the same color below the line of the linkage group.


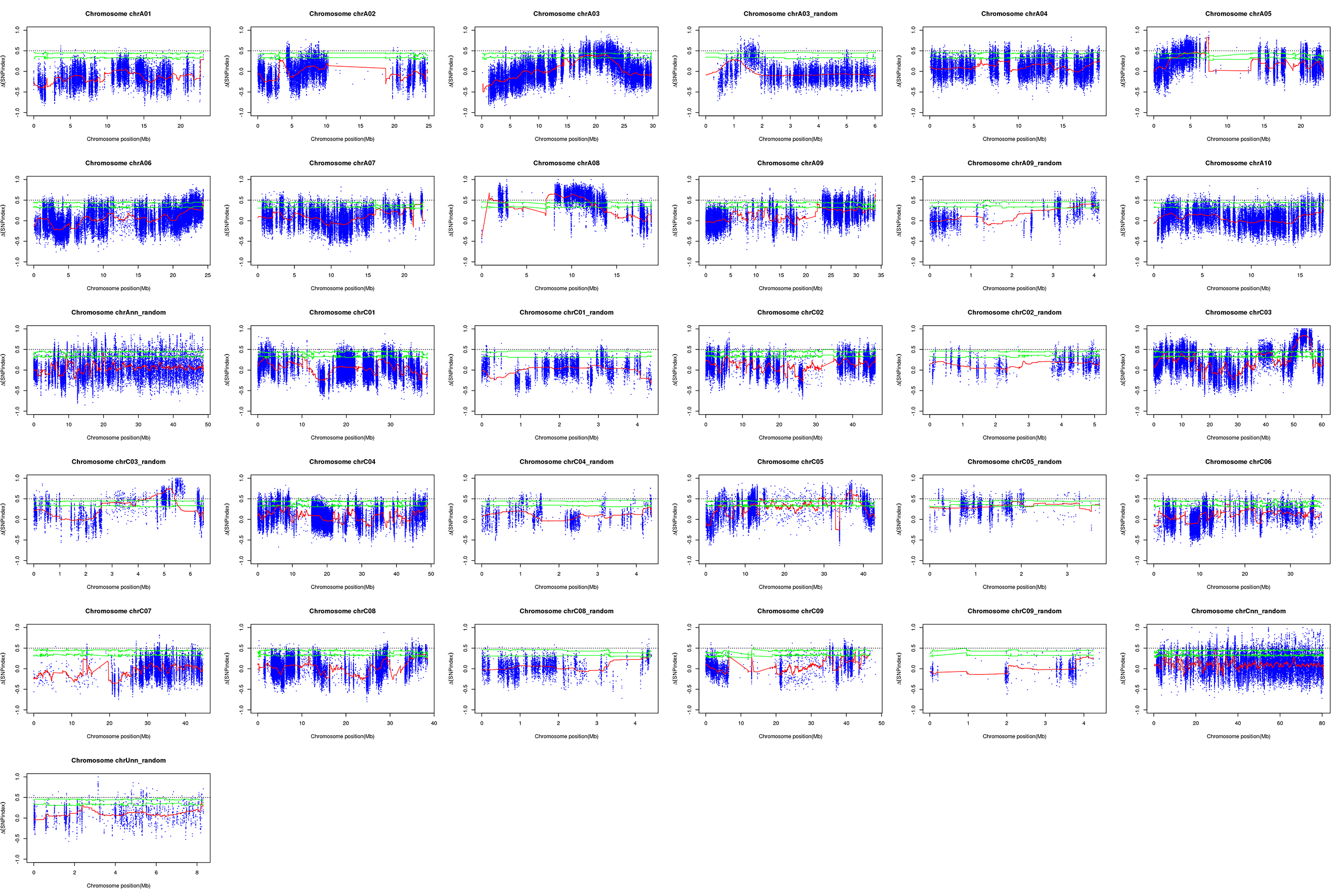


**Figure S3. Δ(SNP-index) plot for all chromosomes (including chromosome_random).** The Δ(SNP-index) plot obtained by subtraction of low SOC from the high SOC SNP-index for KN DH lines obtained from a cross between KenC-8 and N53-2. Red lines indicate the sliding window average of the 2-Mb interval with a 10-kb increment for the Δ(SNP-index). Statistical confidence intervals under the null hypothesis of no QTLs are shown (two green lines: up: P < 0.05; down: P < 0.01).


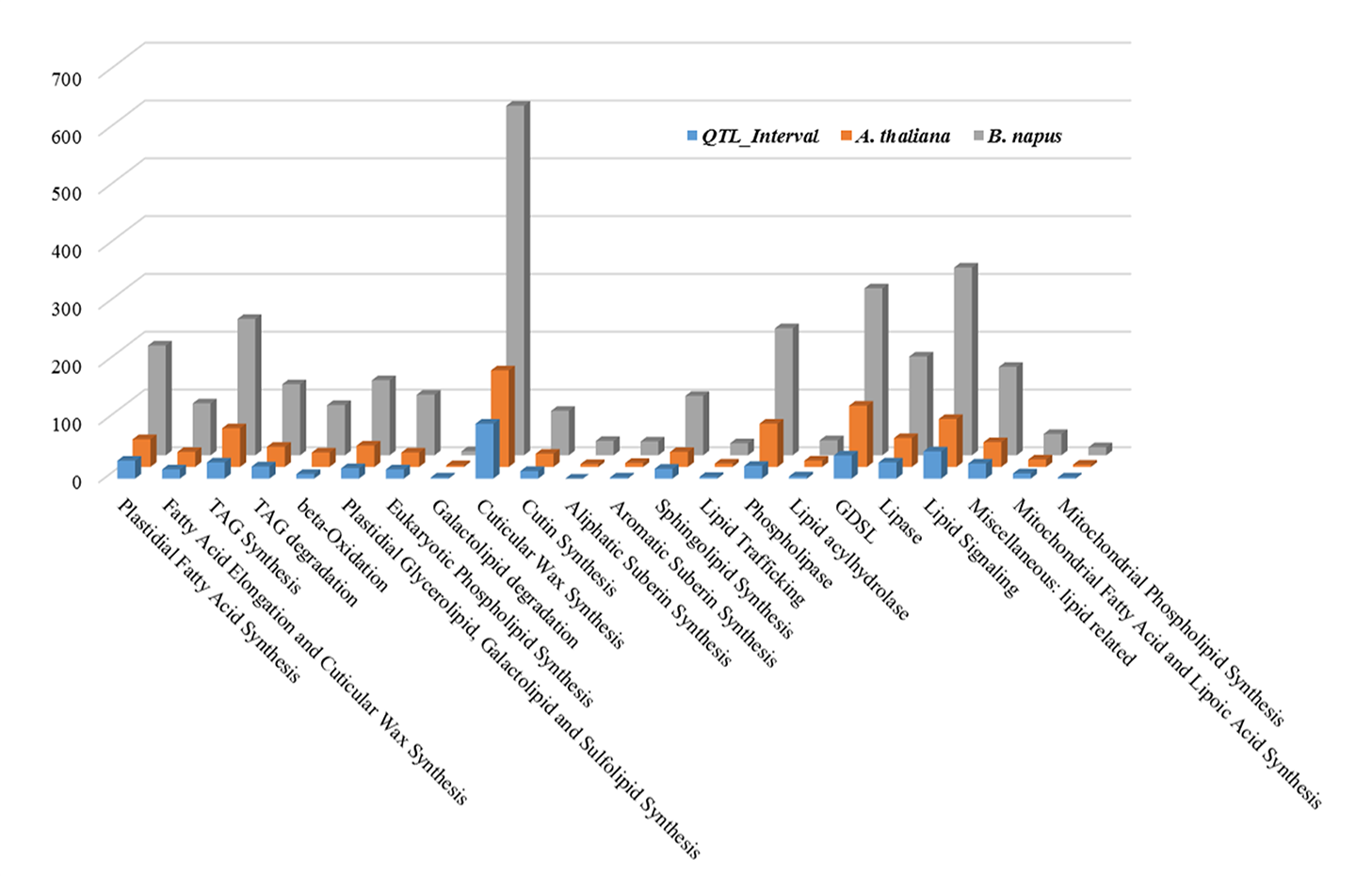


**Figure S4. The number of genes involved in 22 pathways related to acyl-lipid metabolism in the QTL confidence interval, the whole *B. napus* and the *A. thaliana* genome.**

**
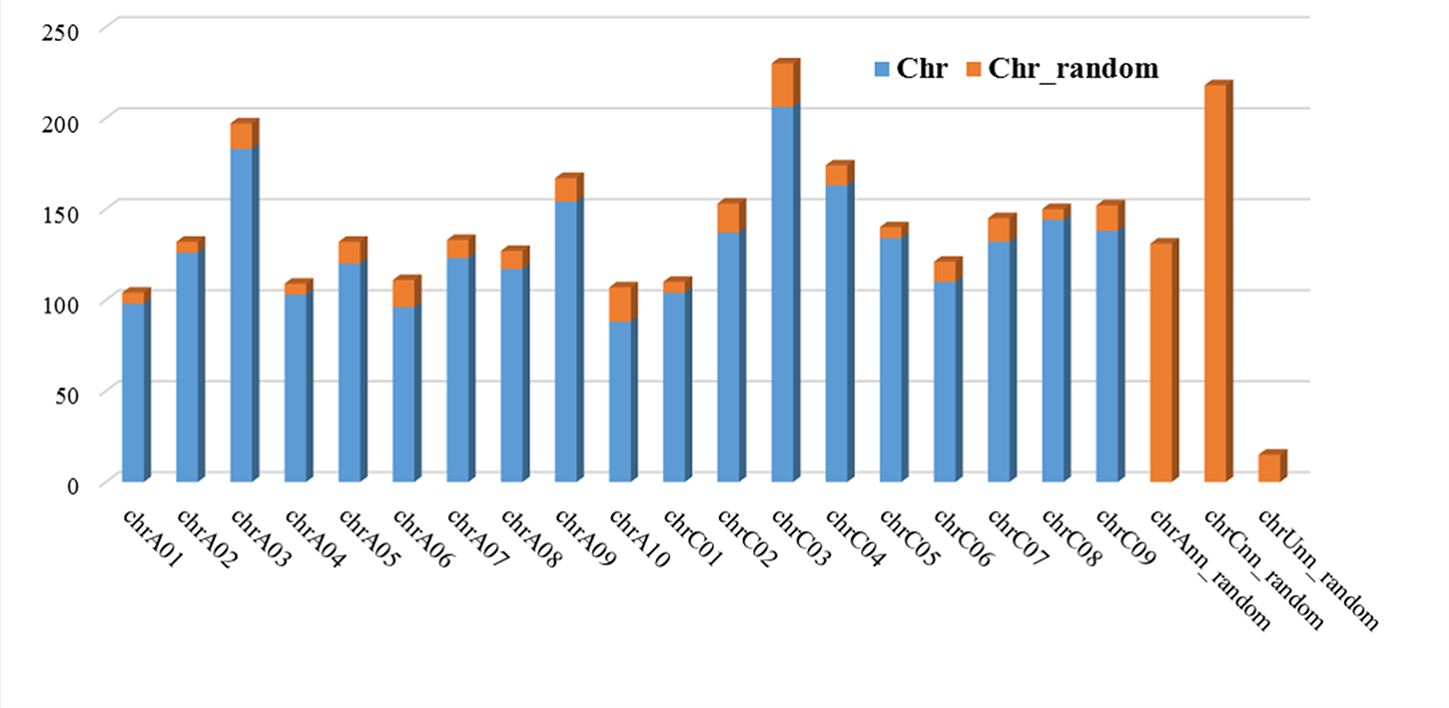
**

**Figure S5 The whole genomic distribution of genes annotated to be involved in acyl-lipid metabolism in *B. napus*.** The vertical axis represents the number of genes related to acyl-lipid metabolism on each chromosome or chromosome_random.


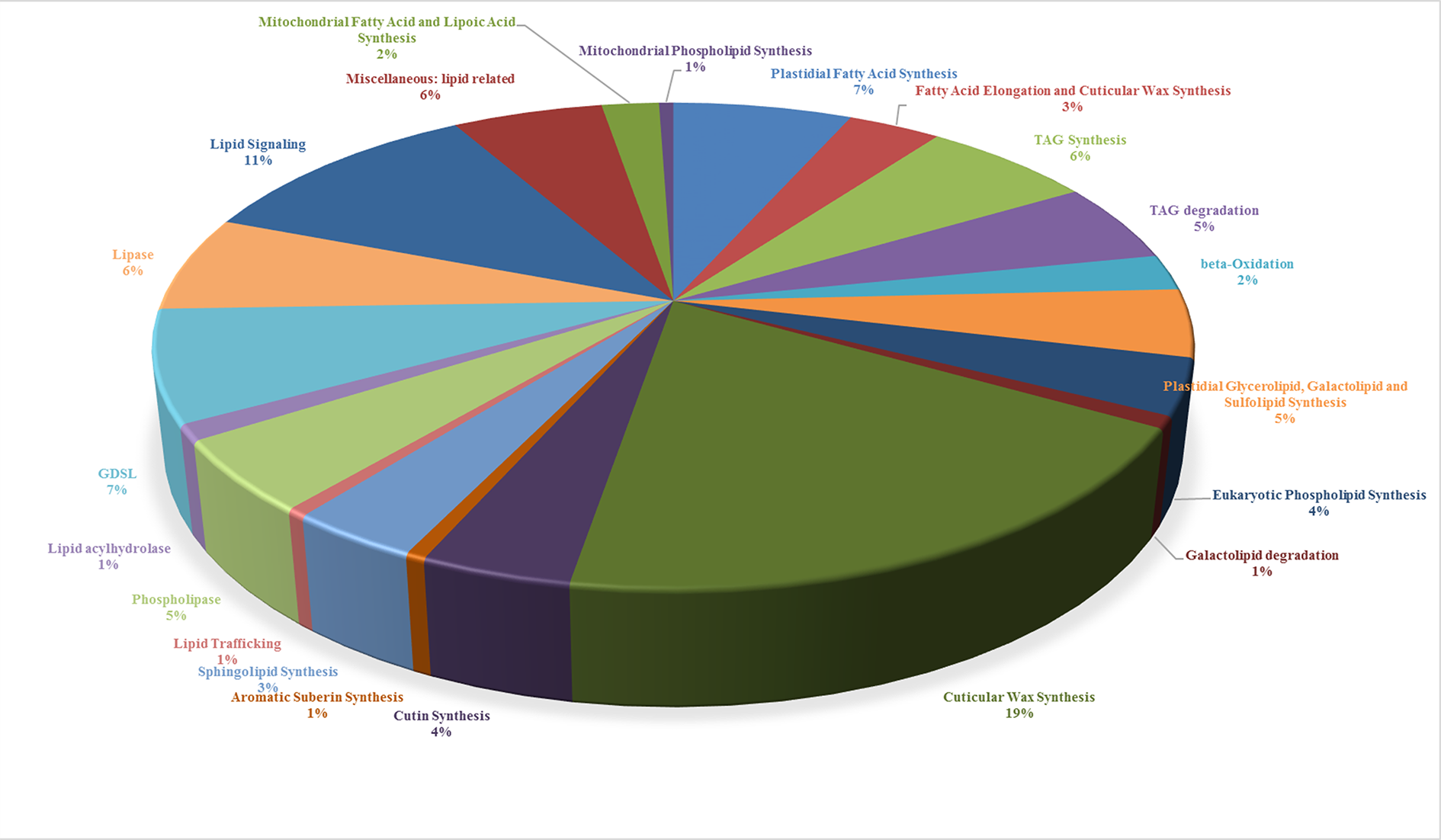


**Figure S6. Functional category and distribution ratio of potential candidate genes related to acyl-lipid metabolism underlying SOC-QTLs.**

**
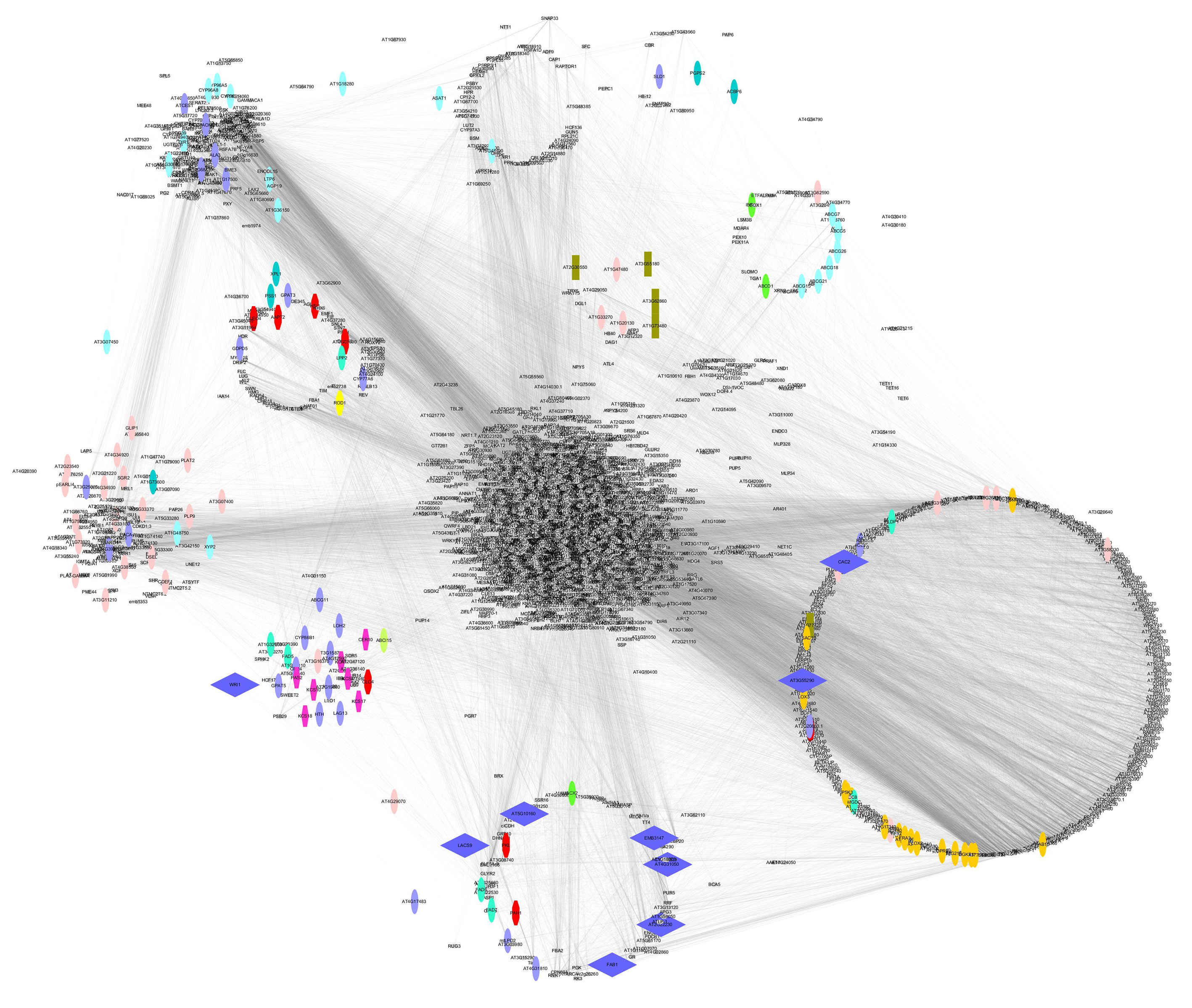
**

**Figure S7. The whole interaction network of all genes underlying the SOC-QTL regions.** Various shapes with different colors represent genes that are directly related to lipid metabolism. Genes that are not involved directly in lipid metabolism are represented by gray ovals.

| **Table S1. Phenotypic variation of SOC and SPC for the two parents and the KNDH population in the four investigated environments.** | | | | | | | | | | | | | |  |
| --- | --- | --- | --- | --- | --- | --- | --- | --- | --- | --- | --- | --- | --- | --- |
| **Traits** | **Materials** |  | **Environments** | | | | | | | | | | | |
|  |  |  | **08DL** | **09DL** | **10DL** | **11DL** | **12DL** | **13DL** | **10GS** | **11GS** | **10HG** | **11WH** | **12WH** | **13WH** |
| **SOC** | **N53-2** |  | 44.67 | 51.51 | 54.77 | 51.43 | 53.37 | 52.6 | * | * | 39.96 | 40.54 | 53.65 | 55.92 |
|  | **Ken-C8** |  | 41.57 | 41.57 | 43.07 | 38.96 | 41.9 | 41.53 | 40.53 | 40.93 | 36.15 | 26.16 | 42.34 | 45.03 |
|  | **DH** | Mean | 44.88±2.30 | 46.91±2.41 | 48.07±2.43 | 45.50±2.84 | 47.74±2.29 | 47.39±2.59 | 46±2.45 | 46.22±2.69 | 40.25±2.78 | 35.17±3.60 | 51.19±2.54 | 49.26±3.72 |
|  |  | max | 51.7 | 53.78 | 54.57 | 52.11 | 52.98 | 53.74 | 51.36 | 52.76 | 47.7 | 44.22 | 57.17 | 56.97 |
|  |  | min | 38.22 | 40.13 | 39.9 | 36.62 | 38.78 | 38.88 | 39.39 | 39.38 | 30.68 | 22.67 | 42.23 | 33 |
| **SPC** | **N53-2** |  |  | 19.63 | 17.27 | 19.5 | 19.61 | 19.47 | * | * | 25.08 | 25.37 | 18.2 | 19.47 |
|  | **Ken-C8** |  |  | 24.44 | 22.93 | 22.75 | 24.27 | 25.26 | 21.09 | 21.45 | 26.85 | 30.39 | 20.82 | 21.81 |
|  | **DH** | mean |  | 22.84±1.11 | 21.52±1.41 | 22.16±1.22 | 22.99±1.73 | 22.43±1.25 | 18.75±1.26 | 19.86±1.41 | 24.93±1.49 | 27.66±1.54 | 17.93±1.33 | 22.14±2.35 |
|  |  | max |  | 26.18 | 28.42 | 27.86 | 28.8 | 25.77 | 23.35 | 25 | 30.43 | 34.73 | 23.9 | 32.65 |
|  |  | min |  | 19.81 | 18.08 | 18.84 | 20.2 | 19.43 | 16.12 | 16.89 | 20.8 | 23.96 | 15 | 17.48 |
| ^*^Mean missing | |  |  |  |  |  |  |  |  |  |  |  |  |  |

| **Table S2. Correlation analysis of SOC and SPC of the KNDH populations in 11 environments.** | | |
| --- | --- | --- |
| Env. | Individual (NO.) | Pearson Index |
| 09DL | 219 | -0.595** |
| 10DL | 217 | -0.684** |
| 10GS | 76 | -0.524** |
| 10HG | 208 | -0.749** |
| 11DL | 219 | -0.632** |
| 11GS | 124 | -0.772** |
| 11WH | 205 | -0.529** |
| 12DL | 219 | -0.723** |
| 12WH | 212 | -0.537** |
| 13DL | 219 | -0.676** |
| 13WH | 219 | -0.763** |
| ** Significant at P = 0.001. | |  |

|  | **Table S3. Identified QTLs and consensus QTLs detected by high-density integrated map for seed oil and protein contents in the KN population.** | | | | | | | | | | | | | | | | | | | | | |  |
| --- | --- | --- | --- | --- | --- | --- | --- | --- | --- | --- | --- | --- | --- | --- | --- | --- | --- | --- | --- | --- | --- | --- | --- |
|  |  | **Consensus QTL** | | | | | | | | | | | | | **Identified QTL** | | | | | | | | |
|  |  | **QTL** | | **Chr** | **Peak position** | | **Confidence interval** | | **LOD value** | **Additive effect** | | **PV (R^2^,%)** | **Environment** | | **QTL** | | **Peak position** | **Confidence interval** | **LOD value** | | **Additive effect** | | **PV (R^2^,%)** |
|  | **SOC** | ***cqOC-A1*** | | A01 | 109.41 | | 104.00-112.10 | | 4.32 | 0.72 | | 9.59 | *08DL* | | ***qOC-08DL1*** | | 109.41 | 104-112.1 | 4.32 | | 0.72 | | 9.59 |
|  |  | ***cqOC-A2-1*** | | A02 | 2.30 | | 0 - 7.08 | | 2.06-2.34 | -0.41- -0.43 | | 1.97-2.22 | *10DL/11DL* | | ***qOC-10DL2*** | | 0.01 | 0-12.8 | 2.34 | | -0.41 | | 2.22 |
|  |  |  |  |  |  |  |  |  |  |  |  |  |  |  | ***qOC-11DL2*** | | 5.21 | 0-14.4 | 2.06 | | -0.43 | | 1.97 |
|  |  | ***cqOC-A2-2*** | | A02 | 107.71 | | 105.80-109.20 | | 7.70 | -1.2 | | 14.18 | *11GS* | | ***qOC-11GS2-1*** | | 107.71 | 105.8-109.2 | 7.70 | | -1.20 | | 14.18 |
|  |  | ***cqOC-A2-3*** | | A02 | 116.26 | | 113.42 - 119.10 | | 2.30-7.35 | -0.66- -1.16 | | 3.43-13.72 | *11GS/10GS/13WH* | | ***qOC-11GS2-2*** | | 115.81 | 114.8-122.9 | 7.35 | | -1.16 | | 13.73 |
|  |  |  |  |  |  |  |  |  |  |  |  |  |  |  | ***qOC-10GS2*** | | 116.61 | 113-123.9 | 2.30 | | -0.66 | | 4.69 |
|  |  |  |  |  |  |  |  |  |  |  |  |  |  |  | ***qOC-13WH2*** | | 116.81 | 112.8-124.4 | 3.02 | | -0.70 | | 3.43 |
|  |  | ***cqOC-A3-1*** | | A03 | 26.01 | | 23.58 - 28.43 | | 2.13-3.37 | -0.52- -0.68 | | 4.36-6.00 | *10GS/11GS* | | ***qOC-10GS3*** | | 26.01 | 22.1-28 | 2.13 | | -0.52 | | 4.36 |
|  |  |  |  |  |  |  |  |  |  |  |  |  |  |  | ***qOC-11GS3-1*** | | 26.01 | 19.5-28 | 3.37 | | -0.68 | | 6.00 |
|  |  | ***cqOC-A3-2*** | | A03 | 32.51 | | 31.9-35.8 | | 3.38 | -0.68 | | 6.02 | *11GS* | | ***qOC-11GS3-2*** | | 32.51 | 31.9-35.8 | 3.38 | | -0.68 | | 6.02 |
|  |  | ***cqOC-A3-3*** | | A03 | 122.37 | | 120.45 - 124.29 | | 2.28-3.50 | 0.35-0.50 | | 2.12-3.13 | *09DL/11DL/12DL* | | ***qOC-09DL3-1*** | | 119.31 | 116.8-127.7 | 2.28 | | 0.35 | | 2.12 |
|  |  |  |  |  |  |  |  |  |  |  |  |  |  |  | ***qOC-11DL3-1*** | | 122.81 | 119.1-126.6 | 2.69 | | 0.51 | | 2.55 |
|  |  |  |  |  |  |  |  |  |  |  |  |  |  |  | ***qOC-12DL3-1*** | | 122.81 | 118.3-123.2 | 3.50 | | 0.49 | | 3.13 |
|  |  | ***cqOC-A3-4*** | | A03 | 151.81 | | 150.70-153.90 | | 3.66 | 0.58 | | 3.44 | *11DL* | | ***qOC-11DL3-2*** | | 151.81 | 150.7-153.9 | 3.66 | | 0.58 | | 3.44 |
|  |  | ***cqOC-A3-5*** | | A03 | 182.85 | | 182.10 - 183.59 | | 2.14-2.45 | 0.37-0.49 | | 1.94-2.41 | *11DL/12DL* | | ***qOC-11DL3-3*** | | 182.71 | 181.7-183.2 | 2.45 | | 0.49 | | 2.41 |
|  |  |  |  |  |  |  |  |  |  |  |  |  |  |  | ***qOC-12DL3-2*** | | 185.71 | 173.8-195.7 | 2.14 | | 0.37 | | 1.94 |
|  |  | ***cqOC-A3-6*** | | A03 | 191.71 | | 183.20-195.70 | | 3.72 | 0.63 | | 4.12 | *11DL* | | ***qOC-11DL3-4*** | | 191.71 | 183.2-195.7 | 3.72 | | 0.63 | | 4.12 |
|  |  | ***cqOC-A3-7*** | | A03 | 204.14 | | 200.26 - 208.03 | | 2.14-3.59 | 0.38-0.73 | | 2.08-3.76 | *11DL/12DL/13WH/12DL* | | ***qOC-11DL3-5*** | | 199.71 | 195.7-209.6 | 3.59 | | 0.60 | | 3.76 |
|  |  |  |  |  |  |  |  |  |  |  |  |  |  |  | ***qOC-09DL3-2*** | | 203.51 | 189.1-215.3 | 2.95 | | 0.38 | | 2.72 |
|  |  |  |  |  |  |  |  |  |  |  |  |  |  |  | ***qOC-13WH3*** | | 205.51 | 195.1-209.6 | 3.00 | | 0.73 | | 3.74 |
|  |  |  |  |  |  |  |  |  |  |  |  |  |  |  | ***qOC-12DL3-3*** | | 207.51 | 195.7-209.6 | 2.14 | | 0.39 | | 2.08 |
|  |  | ***cqOC-A8-1*** | | A08 | 1.47 | | 1.08 - 1.85 | | 2.40-3.48 | 0.40-0.50 | | 2.16-3.25 | *12DL/10DL* | | ***qOC-12DL8-3*** | | 1.39 | 1.2-2.2 | 2.40 | | 0.40 | | 2.16 |
|  |  |  |  |  |  |  |  |  |  |  |  |  |  |  | ***qOC-10DL8-3*** | | 1.59 | 1.40-2.60 | 3.48 | | 0.50 | | 3.25 |
|  |  | ***cqOC-A8-2*** | | A08 | 8.61 | | 7.34 - 9.88 | | 2.331-5.32 | 0.38-0.62 | | 2.45-5.32 | *10GS/09DL/13DL/10DL/12DL* | | ***qOC-10GS8-2*** | | 7.19 | 2.60-8.40 | 2.33 | | 0.57 | | 5.32 |
|  |  |  |  |  |  |  |  |  |  |  |  |  |  |  | ***qOC-09DL8-2*** | | 8.19 | 4-9.4 | 2.86 | | 0.38 | | 2.64 |
|  |  |  |  |  |  |  |  |  |  |  |  |  |  |  | ***qOC-13DL8-2*** | | 8.19 | 4-9.4 | 5.32 | | 0.62 | | 5.24 |
|  |  |  |  |  |  |  |  |  |  |  |  |  |  |  | ***qOC-10DL8-2*** | | 9.89 | 7.2-12.1 | 4.16 | | 0.56 | | 4.02 |
|  |  |  |  |  |  |  |  |  |  |  |  |  |  |  | ***qOC-12DL8-2*** | | 9.89 | 7.2-15.5 | 2.55 | | 0.42 | | 2.45 |
|  |  | ***cqOC-A8-3*** | | A08 | 12.32 | | 10.85 - 13.78 | | 2.60-3.72 | 0.58-0.72 | | 3.50-6.82 | *11GS/11DL/10GS* | | ***qOC-11GS8-3*** | | 11.79 | 7.7-15.5 | 3.60 | | 0.72 | | 6.82 |
|  |  |  |  |  |  |  |  |  |  |  |  |  |  |  | ***qOC-11DL8*** | | 12.19 | 9.4-15.5 | 3.72 | | 0.58 | | 3.50 |
|  |  |  |  |  |  |  |  |  |  |  |  |  |  |  | ***qOC-10GS8-1*** | | 12.49 | 11.8-15.5 | 2.60 | | 0.59 | | 5.92 |
|  |  | ***cqOC-A8-4*** | | A08 | 18.01 | | 17.61 - 18.41 | | 3.45-5.12 | 0.47-0.85 | | 3.09-9.36 | *12DL/10DL/11GS/13DL* | | ***qOC-12DL8-1*** | | 16.99 | 15.6-19.1 | 3.45 | | 0.47 | | 3.09 |
|  |  |  |  |  |  |  |  |  |  |  |  |  |  |  | ***qOC-10DL8-1*** | | 17.49 | 12.5-19.1 | 3.58 | | 0.51 | | 3.33 |
|  |  |  |  |  |  |  |  |  |  |  |  |  |  |  | ***qOC-11GS8-2*** | | 17.49 | 17-19.1 | 5.13 | | 0.85 | | 9.36 |
|  |  |  |  |  |  |  |  |  |  |  |  |  |  |  | ***qOC-13DL8-1*** | | 18.19 | 18-18.9 | 3.99 | | 0.53 | | 3.96 |
|  |  | ***cqOC-A8-5*** | | A08 | 20.01 | | 19.53 - 20.49 | | 2.84-4.83 | 0.37-0.67 | | 2.62-5.89 | *10HG/09DL* | | ***qOC-10HG8*** | | 19.99 | 19.1-20.1 | 4.83 | | 0.67 | | 5.89 |
|  |  |  |  |  |  |  |  |  |  |  |  |  |  |  | ***qOC-09DL8-1*** | | 20.39 | 19.7-23.5 | 2.84 | | 0.37 | | 2.62 |
|  |  | ***cqOC-A8-6*** | | A08 | 21.91 | | 20.9-23.2 | | 3.19-5.34 | 0.59-0.86 | | 3.90-9.76 | *12WH/11GS* | | ***qOC-12WH8*** | | 21.29 | 20.9-22 | 3.19 | | 0.59 | | 3.90 |
|  |  |  |  |  |  |  |  |  |  |  |  |  |  |  | ***qOC-11GS8-1*** | | 23.49 | 20.4-24.2 | 5.34 | | 0.86 | | 9.76 |
|  |  | ***cqOC-A9-1*** | | A09 | 1.17 | | 0.19 - 2.14 | | 2.04-2.69 | 0.36-0.47 | | 2.14-2.52 | *10DL/09DL/10HG* | | ***qOC-10DL9-1*** | | 0.71 | 0-2.8 | 2.69 | | 0.47 | | 2.52 |
|  |  |  |  |  |  |  |  |  |  |  |  |  |  |  | ***qOC-09DL9-1*** | | 1.61 | 0-2.8 | 2.30 | | 0.36 | | 2.14 |
|  |  |  |  |  |  |  |  |  |  |  |  |  |  |  | ***qOC-10HG9-1*** | | 1.61 | 0-12.3 | 2.04 | | 0.45 | | 2.37 |
|  |  | ***cqOC-A9-2*** | | A09 | 23.27 | | 21.81 - 24.73 | | 2.19-4.38 | 0.42-0.57 | | 2.54-4.18 | *09DL/10DL/10HG/13DL* | | ***qOC-09DL9-2*** | | 23.21 | 19.6-24.8 | 3.32 | | 0.42 | | 3.35 |
|  |  |  |  |  |  |  |  |  |  |  |  |  |  |  | ***qOC-10DL9-2*** | | 23.21 | 19.6-24.4 | 4.36 | | 0.57 | | 4.18 |
|  |  |  |  |  |  |  |  |  |  |  |  |  |  |  | ***qOC-10HG9-2*** | | 23.41 | 12.3-30.8 | 2.19 | | 0.45 | | 2.55 |
|  |  |  |  |  |  |  |  |  |  |  |  |  |  |  | ***qOC-13DL9-1*** | | 23.41 | 22.1-27.5 | 4.38 | | 0.54 | | 4.04 |
|  |  | ***cqOC-A9-3*** | | A09 | 31.21 | | 30.4-32.2 | | 4.21 | 0.53 | | 3.88 | *13DL* | | ***qOC-13DL9-2*** | | 31.21 | 30.4-32.2 | 4.21 | | 0.53 | | 3.88 |
|  |  | ***cqOC-A9-4*** | | A09 | 34.26 | | 32.36 - 36.17 | | 2.45-5.35 | 0.41-0.72 | | 2.30-5.40 | *11DL/10DL/09DL* | | ***qOC-11DL9-1*** | | 33.11 | 32-37.3 | 5.35 | | 0.72 | | 5.40 |
|  |  |  |  |  |  |  |  |  |  |  |  |  |  |  | ***qOC-10DL9-3*** | | 33.91 | 32-38.7 | 2.45 | | 0.42 | | 2.30 |
|  |  |  |  |  |  |  |  |  |  |  |  |  |  |  | ***qOC-09DL9-3*** | | 38.71 | 33.9-43.4 | 3.45 | | 0.41 | | 3.17 |
|  |  | ***cqOC-A9-5*** | | A09 | 67.61 | | 67.1-73.1 | | 3.22 | -0.56 | | 3.04 | *11DL* | | ***qOC-11DL9-2*** | | 67.61 | 67.1-73.1 | 3.22 | | -0.56 | | 3.04 |
|  |  | ***cqOC-A9-6*** | | A09 | 95.11 | | 94.9-96.7 | | 4.51 | 0.60 | | 4.82 | *12DL* | | ***qOC-12DL9-1*** | | 95.11 | 94.9-96.7 | 4.51 | | 0.60 | | 4.82 |
|  |  | ***cqOC-A9-7*** | | A09 | 98.41 | | 97.23 - 99.58 | | 2.96-4.80 | 0.49-0.68 | | 3.06-5.14 | *10DL/12WH* | | ***qOC-10DL9-4*** | | 97.61 | 95.4-99 | 2.96 | | 0.49 | | 3.06 |
|  |  |  |  |  |  |  |  |  |  |  |  |  |  |  | ***qOC-12WH9-1*** | | 99.01 | 96.8-99.9 | 4.80 | | 0.68 | | 5.14 |
|  |  | ***cqOC-A9-8*** | | A09 | 100.71 | | 99.9-101.2 | | 9.19 | 0.86 | | 10.05 | *13DL* | | ***qOC-13DL9-3*** | | 100.71 | 99.9-101.2 | 9.19 | | 0.86 | | 10.05 |
|  |  | ***cqOC-A9-9*** | | A09 | 106.28 | | 105.79 - 106.77 | | 2.76-14.19 | 0.64-1.05 | | 4.88-14.94 | *09DL/11GS/13DL/12DL/12WH/10GS* | | ***qOC-09DL9-4*** | | 104.81 | 102.9-105 | 10.75 | | 0.78 | | 11.08 |
|  |  |  |  |  |  |  |  |  |  |  |  |  |  |  | ***qOC-11GS9*** | | 105.61 | 102.1-111 | 2.76 | | 0.64 | | 4.88 |
|  |  |  |  |  |  |  |  |  |  |  |  |  |  |  | ***qOC-13DL9-4*** | | 106.31 | 105.3-106.8 | 14.19 | | 1.05 | | 14.94 |
|  |  |  |  |  |  |  |  |  |  |  |  |  |  |  | ***qOC-12DL9-2*** | | 107.01 | 106.5-109.3 | 13.00 | | 1.00 | | 13.42 |
|  |  |  |  |  |  |  |  |  |  |  |  |  |  |  | ***qOC-12WH9-2*** | | 107.01 | 106.5-108.7 | 8.20 | | 0.89 | | 8.57 |
|  |  |  |  |  |  |  |  |  |  |  |  |  |  |  | ***qOC-10GS9-1*** | | 108.91 | 106.3-111.7 | 4.57 | | 0.85 | | 10.94 |
|  |  | ***cqOC-A9-10*** | | A09 | 112.70 | | 111.62 - 113.79 | | 2.87-11.48 | 0.69-0.81 | | 3.32-12.41 | *09DL/11DL/12WH/13WH* | | ***qOC-09DL9-5*** | | 111.61 | 109.6-113.8 | 11.48 | | 0.81 | | 12.41 |
|  |  |  |  |  |  |  |  |  |  |  |  |  |  |  | ***qOC-11DL9-3*** | | 113.11 | 112.4-116.2 | 7.65 | | 0.89 | | 7.86 |
|  |  |  |  |  |  |  |  |  |  |  |  |  |  |  | ***qOC-12WH9-3*** | | 113.11 | 112.2-117.6 | 6.44 | | 0.79 | | 6.80 |
|  |  |  |  |  |  |  |  |  |  |  |  |  |  |  | ***qOC-13WH9*** | | 113.11 | 112.5-116.9 | 2.87 | | 0.69 | | 3.32 |
|  |  | ***cqOC-A9-11*** | | A09 | 116.38 | | 116.16 - 116.59 | | 7.33-7.66 | 0.64-0.79 | | 7.76-8.46 | *13DL/09DL* | | ***qOC-13DL9-5*** | | 116.31 | 116-116.5 | 7.66 | | 0.79 | | 8.46 |
|  |  |  |  |  |  |  |  |  |  |  |  |  |  |  | ***qOC-09DL9-6*** | | 116.61 | 116-116.9 | 7.33 | | 0.64 | | 7.76 |
|  |  | ***cqOC-A9-12*** | | A09 | 122.01 | | 119.88 - 124.13 | | 2.60-7.21 | 0.66-0.89 | | 7.25-8.05 | *10GS/11DL* | | ***qOC-10GS9-2*** | | 122.01 | 116-122.9 | 2.60 | | 0.66 | | 7.25 |
|  |  |  |  |  |  |  |  |  |  |  |  |  |  |  | ***qOC-11DL9-4*** | | 122.01 | 117.7-123.1 | 7.21 | | 0.89 | | 8.05 |
|  |  | ***cqOC-A10-1*** | | A010 | 1.11 | | 0 - 2.59 | | 3.64-3.79 | -0.61- -0.64 | | 3.39-4.56 | *10DL/09DL* | | ***qOC-10DL10-1*** | | 0.01 | 0-4.2 | 3.64 | | -0.64 | | 3.39 |
|  |  |  |  |  |  |  |  |  |  |  |  |  |  |  | ***qOC-09DL10*** | | 2.21 | 0-4.2 | 3.79 | | -0.61 | | 4.56 |
|  |  | ***cqOC-A10-2*** | | A010 | 8.71 | | 5.8-16.7 | | 2.80 | -0.68 | | 2.73 | *11DL* | | ***qOC-11DL10*** | | 8.71 | 5.8-16.7 | 2.80 | | -0.68 | | 2.73 |
|  |  | ***cqOC-A10-3*** | | A010 | 18.03 | | 14.35 - 21.71 | | 2.12-3.13 | -0.56- -1.02 | | 2.00-4.43 | *11WH/10DL/12DL* | | ***qOC-11WH10*** | | 14.71 | 8.7-20.4 | 2.41 | | -1.02 | | 4.43 |
|  |  |  |  |  |  |  |  |  |  |  |  |  |  |  | ***qOC-10DL10-2*** | | 20.11 | 18.7-30.1 | 2.12 | | -0.49 | | 2.00 |
|  |  |  |  |  |  |  |  |  |  |  |  |  |  |  | ***qOC-12DL10*** | | 20.41 | 10-26.9 | 3.13 | | -0.56 | | 2.81 |
|  |  | ***cqOC-C2-1*** | | C02 | 84.14 | | 82.1 - 86.18 | | 2.60-3.66 | 0.47-0.82 | | 2.44-8.68 | *10DL/10GS* | | ***qOC-10DL12-1*** | | 82.01 | 79-85.4 | 2.60 | | 0.47 | | 2.44 |
|  |  |  |  |  |  |  |  |  |  |  |  |  |  |  | ***qOC-10GS12-1*** | | 85.61 | 84.6-89.9 | 3.66 | | 0.82 | | 8.68 |
|  |  | ***cqOC-C2-2*** | | C02 | 91.10 | | 89.17 - 93.04 | | 2.95-3.13 | 0.49-0.76 | | 2.76-7.48 | *10DL/10GS* | | ***qOC-10DL12-2*** | | 89.91 | 87.3-92.1 | 2.95 | | 0.49 | | 2.77 |
|  |  |  |  |  |  |  |  |  |  |  |  |  |  |  | ***qOC-10GS12-2*** | | 93.31 | 89.9-96.4 | 3.13 | | 0.76 | | 7.48 |
|  |  | ***cqOC-C2-3*** | | C02 | 100.71 | | 97.6-103.1 | | 3.70 | 0.57 | | 3.90 | *10DL* | | ***qOC-10DL12-3*** | | 100.71 | 97.6-103.1 | 3.70 | | 0.57 | | 3.90 |
|  |  | ***cqOC-C2-4*** | | C02 | 111.08 | | 109.45 - 112.72 | | 2.10-3.72 | 0.33-0.53 | | 1.95-3.50 | *10DL/09DL* | | ***qOC-10DL12-4*** | | 110.81 | 108.6-112.1 | 3.72 | | 0.53 | | 3.50 |
|  |  |  |  |  |  |  |  |  |  |  |  |  |  |  | ***qOC-09DL12*** | | 113.01 | 111.1-120.3 | 2.10 | | 0.33 | | 1.95 |
|  |  | ***cqOC-C3-1*** | | C03 | 144.94 | | 142.23 - 147.64 | | 2.26-2.74 | 0.37-0.4417 | | 2.56-2.20 | *12WH/09DL* | | ***qOC-12WH13-1*** | | 143.11 | 137.4-152.5 | 2.27 | | 0.44 | | 2.20 |
|  |  |  |  |  |  |  |  |  |  |  |  |  |  |  | ***qOC-09DL13-1*** | | 145.21 | 144.1-149.9 | 2.74 | | 0.37 | | 2.56 |
|  |  | ***cqOC-C3-2*** | | C03 | 166.11 | | 163.56 - 168.65 | | 2.35-5.26 | 0.48-0.90 | | 2.24-12.72 | *10GS/11DL* | | ***qOC-10GS13-1*** | | 166.11 | 163.6-169.6 | 5.26 | | 0.90 | | 12.72 |
|  |  |  |  |  |  |  |  |  |  |  |  |  |  |  | ***qOC-11DL13-1*** | | 166.11 | 162-171.6 | 2.35 | | 0.48 | | 2.24 |
|  |  | ***cqOC-C3-3*** | | C03 | 172.61 | | 171.6-173.9 | | 4.09 | 0.82 | | 10.25 | *10GS* | | ***qOC-10GS13-2*** | | 172.61 | 171.6-173.9 | 4.09 | | 0.82 | | 10.25 |
|  |  | ***cqOC-C3-4*** | | C03 | 300.48 | | 299.84 - 301.12 | | 2.69-8.04 | 0.45-1.052 | | 2.52-8.12 | *11WH/10HG/12DL/09DL/10DL/11DL/13DL/13WH* | | ***qOC-11WH13-1*** | | 298.21 | 297.2-300.9 | 3.79 | | 0.84 | | 4.85 |
|  |  |  |  |  |  |  |  |  |  |  |  |  |  |  | ***qOC-10HG13-1*** | | 300.91 | 297-304.2 | 3.82 | | 0.62 | | 4.63 |
|  |  |  |  |  |  |  |  |  |  |  |  |  |  |  | ***qOC-12DL13-1*** | | 300.91 | 298.9-302.2 | 6.42 | | 0.68 | | 6.20 |
|  |  |  |  |  |  |  |  |  |  |  |  |  |  |  | ***qOC-09DL13-2*** | | 301.11 | 300.4-303.4 | 8.04 | | 0.67 | | 8.12 |
|  |  |  |  |  |  |  |  |  |  |  |  |  |  |  | ***qOC-10DL13-2*** | | 301.11 | 297.4-302.2 | 3.47 | | 0.51 | | 3.24 |
|  |  |  |  |  |  |  |  |  |  |  |  |  |  |  | ***qOC-11DL13-2*** | | 301.11 | 300-303.9 | 6.17 | | 0.80 | | 6.54 |
|  |  |  |  |  |  |  |  |  |  |  |  |  |  |  | ***qOC-13DL13-1*** | | 301.11 | 298.9-303.9 | 6.64 | | 0.72 | | 6.74 |
|  |  |  |  |  |  |  |  |  |  |  |  |  |  |  | ***qOC-13WH13-1*** | | 301.11 | 300.9-303.9 | 6.06 | | 1.05 | | 7.26 |
|  |  | ***cqOC-C3-5*** | | C03 | 309.01 | | 308.17 - 309.86 | | 3.08-8.01 | 0.56-0.78 | | 3.84-8.03 | *10HG/09DL/13DL* | | ***qOC-10HG13-2*** | | 308.01 | 304.2-310.1 | 3.08 | | 0.56 | | 3.84 |
|  |  |  |  |  |  |  |  |  |  |  |  |  |  |  | ***qOC-09DL13-3*** | | 309.11 | 308.2-310.1 | 6.42 | | 0.59 | | 6.53 |
|  |  |  |  |  |  |  |  |  |  |  |  |  |  |  | ***qOC-13DL13-2*** | | 309.11 | 305.3-310.1 | 8.01 | | 0.78 | | 8.03 |
|  |  | ***cqOC-C3-6*** | | C03 | 312.33 | | 311.35 - 313.31 | | 3.44-7.86 | 0.51-1.20 | | 3.21-9.32 | *10DL/13WH/12DL/12WH* | | ***qOC-10DL13-3*** | | 311.61 | 310.1-316.1 | 3.44 | | 0.51 | | 3.21 |
|  |  |  |  |  |  |  |  |  |  |  |  |  |  |  | ***qOC-13WH13-2*** | | 312.21 | 310.1-313.9 | 7.86 | | 1.20 | | 9.32 |
|  |  |  |  |  |  |  |  |  |  |  |  |  |  |  | ***qOC-12DL13-2*** | | 312.51 | 310.1-315.3 | 6.56 | | 0.70 | | 6.62 |
|  |  |  |  |  |  |  |  |  |  |  |  |  |  |  | ***qOC-12WH13-2*** | | 312.51 | 311.6-314.4 | 5.58 | | 0.74 | | 5.74 |
|  |  | ***cqOC-C3-7*** | | C03 | 318.41 | | 314.17 - 322.64 | | 4.59-6.23 | 0.60-1.09 | | 4.92-7.86 | *12DL/13DL/13WH* | | ***qOC-12DL13-3*** | | 318.41 | 316.1-330.5 | 4.59 | | 0.60 | | 4.92 |
|  |  |  |  |  |  |  |  |  |  |  |  |  |  |  | ***qOC-13DL13-3*** | | 318.41 | 315.6-330.4 | 6.26 | | 0.72 | | 6.89 |
|  |  |  |  |  |  |  |  |  |  |  |  |  |  |  | ***qOC-13WH13-3*** | | 318.41 | 315.6-330.4 | 6.04 | | 1.09 | | 7.86 |
|  |  | ***cqOC-C5-1*** | | C05 | 71.99 | | 58.2-85.8 | | 5.66 | 0.93 | | 10.86 | *10HG* | | ***qOC-10HG15-2*** | | 71.99 | 58.2-85.8 | 5.66 | | 0.93 | | 10.86 |
|  |  | ***cqOC-C5-2*** | | C05 | 135.39 | | 123.4-136 | | 3.82 | -0.53 | | 3.57 | *10DL* | | ***qOC-10DL15-4*** | | 135.39 | 123.4-136 | 3.82 | | -0.53 | | 3.57 |
|  |  | ***cqOC-C5-3*** | | C05 | 183.19 | | 181.38 - 184.99 | | 2.35-10.20 | 0.45-1.00 | | 2.86-10.71 | *09DL/11DL/11WH* | | ***qOC-09DL15-4*** | | 183.19 | 177.6-183.7 | 3.53 | | 0.45 | | 3.43 |
|  |  |  |  |  |  |  |  |  |  |  |  |  |  |  | ***qOC-11DL15-4*** | | 183.19 | 178.2-183.7 | 10.20 | | 1.00 | | 10.71 |
|  |  |  |  |  |  |  |  |  |  |  |  |  |  |  | ***qOC-11WH15-2*** | | 183.19 | 176.5-184.3 | 2.35 | | 0.64 | | 2.86 |
|  |  | ***cqOC-C5-4*** | | C05 | 191.08 | | 190.1 - 192.06 | | 2.17-10.64 | 0.37-1.02 | | 2.09-11.11 | *11DL/09DL/12WH* | | ***qOC-11DL15-3*** | | 190.19 | 188.8-191.2 | 10.64 | | 1.02 | | 11.11 |
|  |  |  |  |  |  |  |  |  |  |  |  |  |  |  | ***qOC-09DL15-3*** | | 192.89 | 190.8-194.2 | 2.40 | | 0.37 | | 2.36 |
|  |  |  |  |  |  |  |  |  |  |  |  |  |  |  | ***qOC-12WH15-3*** | | 215.39 | 210.7-219.4 | 5.04 | | 0.74 | | 6.13 |
|  |  | ***cqOC-C5-5*** | | C05 | 220.41 | | 219.85 - 220.98 | | 3.83-7.55 | 0.47-0.77 | | 3.90-7.64 | *09DL/10DL/11DL/12DL/12WH* | | ***qOC-09DL15-2*** | | 220.39 | 219.4-220.6 | 7.42 | | 0.65 | | 7.43 |
|  |  |  |  |  |  |  |  |  |  |  |  |  |  |  | ***qOC-10DL15-3*** | | 220.59 | 218.5-224.6 | 7.74 | | 0.77 | | 7.96 |
|  |  |  |  |  |  |  |  |  |  |  |  |  |  |  | ***qOC-11DL15-2*** | | 220.59 | 208.8-224.8 | 2.17 | | 0.47 | | 2.09 |
|  |  |  |  |  |  |  |  |  |  |  |  |  |  |  | ***qOC-12DL15-2*** | | 220.59 | 213-224.6 | 6.71 | | 0.67 | | 6.56 |
|  |  |  |  |  |  |  |  |  |  |  |  |  |  |  | ***qOC-12WH15-2*** | | 222.59 | 219.4-224.6 | 4.69 | | 0.70 | | 5.51 |
|  |  | ***cqOC-C5-6*** | | C05 | 230.24 | | 229.22 - 231.26 | | 2.42-7.33 | 0.54-0.76 | | 2.3-7.43 | *12WH/09DL/10DL/12DL* | | ***qOC-12WH15-1*** | | 229.09 | 225.9-230.8 | 3.83 | | 0.59 | | 3.90 |
|  |  |  |  |  |  |  |  |  |  |  |  |  |  |  | ***qOC-09DL15-1*** | | 230.49 | 225.9-231.2 | 4.77 | | 0.54 | | 4.87 |
|  |  |  |  |  |  |  |  |  |  |  |  |  |  |  | ***qOC-10DL15-2*** | | 230.49 | 225.9-231.2 | 7.55 | | 0.76 | | 7.65 |
|  |  |  |  |  |  |  |  |  |  |  |  |  |  |  | ***qOC-12DL15-1*** | | 230.49 | 228.4-231.2 | 6.01 | | 0.64 | | 5.84 |
|  |  | ***cqOC-C5-7*** | | C05 | 234.02 | | 232.79 - 235.24 | | 2.42-7.33 | 0.50-0.75 | | 2.36-7.43 | *10DL/11DL* | | ***qOC-10DL15-1*** | | 233.99 | 232.9-235.4 | 7.33 | | 0.75 | | 7.43 |
|  |  |  |  |  |  |  |  |  |  |  |  |  |  |  | ***qOC-11DL15-1*** | | 234.79 | 232.9-245.3 | 2.42 | | 0.50 | | 2.36 |
|  |  | ***cqOC-C5-8*** | | C05 | 237.39 | | 234.66 - 240.11 | | 3.46-4.34 | 0.53-0.89 | | 3.87-5.64 | *13DL/13WH* | | ***qOC-13DL15-2*** | | 237.39 | 236.2-245.6 | 3.46 | | 0.53 | | 3.87 |
|  |  |  |  |  |  |  |  |  |  |  |  |  |  |  | ***qOC-13WH15-2*** | | 237.39 | 236.5-243.2 | 4.34 | | 0.89 | | 5.64 |
|  |  | ***cqOC-C5-9*** | | C05 | 250.78 | | 249.56 - 252.01 | | 4.02-4.37 | 0.53-0.85 | | 3.91-5.03 | *13DL/13WH* | | ***qOC-13DL15-1*** | | 250.79 | 246.5-251.6 | 4.02 | | 0.53 | | 3.91 |
|  |  |  |  |  |  |  |  |  |  |  |  |  |  |  | ***qOC-13WH15-1*** | | 250.79 | 248.8-251.6 | 4.37 | | 0.85 | | 5.03 |
|  |  | ***cqOC-C5-10*** | | C05 | 258.99 | | 257.20-259.40 | | 4.21 | 0.90 | | 5.68 | *11WH* | | ***qOC-11WH15-1*** | | 258.99 | 257.2-259.4 | 4.21 | | 0.90 | | 5.68 |
|  |  | ***cqOC-C6-1*** | | C06 | 23.98 | | 15.25 - 32.72 | | 2.66-3.26 | 0.47-0.85 | | 2.80-5.03 | *11WH/13DL* | | ***qOC-11WH16*** | | 18.91 | 10.6-31.6 | 3.27 | | 0.85 | | 5.03 |
|  |  |  |  |  |  |  |  |  |  |  |  |  |  |  | ***qOC-13DL16-1*** | | 35.41 | 21.6-53.1 | 2.66 | | 0.47 | | 2.80 |
|  |  | ***cqOC-C6-2*** | | C06 | 149.34 | | 147.65 - 151.02 | | 2.29-4.44 | 0.50-0.83 | | 2.79-4.32 | *10HG/13DL/13WH* | | ***qOC-10HG16-1*** | | 149.01 | 143.9-152.1 | 2.29 | | 0.51 | | 2.79 |
|  |  |  |  |  |  |  |  |  |  |  |  |  |  |  | ***qOC-13DL16-2*** | | 149.41 | 148.7-152.8 | 4.44 | | 0.58 | | 4.06 |
|  |  |  |  |  |  |  |  |  |  |  |  |  |  |  | ***qOC-13WH16-1*** | | 149.41 | 143.5-152.1 | 3.75 | | 0.83 | | 4.32 |
|  |  | ***cqOC-C6-3*** | | C06 | 159.20 | | 152.6 - 162.4 | | 2.03-3.72 | 0.73-1.48 | | 3.01-3.67 | *13DL/12DL/13WH* | | ***qOC-13DL16-3*** | | 154.61 | 152.8-157.4 | 3.72 | | 0.53 | | 3.41 |
|  |  |  |  |  |  |  |  |  |  |  |  |  |  |  | ***qOC-12DL16-1*** | | 159.41 | 152.2-164.9 | 2.03 | | 1.48 | | 3.67 |
|  |  |  |  |  |  |  |  |  |  |  |  |  |  |  | ***qOC-13WH16-2*** | | 159.41 | 157.4-166.6 | 2.40 | | 0.73 | | 3.01 |
|  |  | ***cqOC-C6-4*** | | C06 | 160.61 | | 155.2-169.8 | | 3.61-4.08 | 0.60-0.68 | | 3.73-4.53 | *10HG/13DL* | | ***qOC-10HG16-2*** | | 160.61 | 154.6-162.9 | 3.61 | | 0.68 | | 4.35 |
|  |  |  |  |  |  |  |  |  |  |  |  |  |  |  | ***qOC-13DL16-4*** | | 160.61 | 157.4-170.3 | 4.08 | | 0.60 | | 3.73 |
|  |  | ***cqOC-C6-5*** | | C06 | 181.81 | | 180.18 - 183.45 | | 2.98-4.35 | 0.52-0.75 | | 2.75-4.58 | *13DL/10DL/11DL* | | ***qOC-13DL16-5*** | | 179.41 | 175.9-181.3 | 2.99 | | 0.52 | | 2.75 |
|  |  |  |  |  |  |  |  |  |  |  |  |  |  |  | ***qOC-10DL16-1*** | | 183.21 | 180.7-185.8 | 3.42 | | 0.63 | | 3.99 |
|  |  |  |  |  |  |  |  |  |  |  |  |  |  |  | ***qOC-11DL16-1*** | | 183.21 | 177.5-184.4 | 4.35 | | 0.75 | | 4.58 |
|  |  | ***cqOC-C6-6*** | | C06 | 188.71 | | 187.58 - 188.96 | | 2.39-13.53 | 0.37-4.85 | | 3.78-22.24 | *12DL/10GS/09DL/10DL/11DL/13DL* | | ***qOC-12DL16-2*** | | 187.81 | 185.8-187.9 | 13.53 | | 4.85 | | 22.24 |
|  |  |  |  |  |  |  |  |  |  |  |  |  |  |  | ***qOC-10GS16*** | | 187.91 | 186-191.7 | 4.33 | | 0.90 | | 10.38 |
|  |  |  |  |  |  |  |  |  |  |  |  |  |  |  | ***qOC-09DL16*** | | 188.71 | 185.8-191.7 | 2.39 | | 0.37 | | 2.21 |
|  |  |  |  |  |  |  |  |  |  |  |  |  |  |  | ***qOC-10DL16-2*** | | 188.71 | 188.3-191.3 | 6.71 | | 0.84 | | 7.64 |
|  |  |  |  |  |  |  |  |  |  |  |  |  |  |  | ***qOC-11DL16-2*** | | 188.71 | 188.2-191.4 | 7.55 | | 0.94 | | 7.78 |
|  |  |  |  |  |  |  |  |  |  |  |  |  |  |  | ***qOC-13DL16-6*** | | 188.71 | 185.8-191.7 | 4.12 | | 0.57 | | 3.78 |
|  |  | ***cqOC-C6-7*** | | C6 | 195.61 | | 195.2-197 | | 5.41 | 0.80 | | 5.65 | 11DL | | ***qOC-11DL16-3*** | | 195.61 | 195.2-197 | 5.41 | | 0.80 | | 5.65 |
|  |  | ***cqOC-C6-8*** | | C06 | 199.11 | | 198.88 - 199.33 | | 5.972-6.45 | -2.052 - -2.12 | | 6.17-6.24 | *10DL/12DL* | | ***qOC-10DL16-3*** | | 199.11 | 198.7-199.3 | 5.97 | | -2.12 | | 6.17 |
|  |  |  |  |  |  |  |  |  |  |  |  |  |  |  | ***qOC-12DL16-3*** | | 199.11 | 198.8-199.5 | 6.45 | | -2.05 | | 6.24 |
|  |  | ***cqOC-C6-9*** | | C06 | 201.31 | | 200.4-202.3 | | 3.99 | 0.57 | | 3.64 | *13DL* | | ***qOC-13DL16-7*** | | 201.31 | 200.4-202.3 | 3.99 | | 0.57 | | 3.64 |
|  |  | ***cqOC-C6-10*** | | C06 | 203.80 | | 203.56 - 204.05 | | 3.62-4.98 | -1.99 - -2.06 | | 3.42-4.88 | *11DL/12DL* | | ***qOC-11DL16-4*** | | 203.81 | 203.5-204.4 | 3.62 | | -1.99 | | 3.42 |
|  |  |  |  |  |  |  |  |  |  |  |  |  |  |  | ***qOC-12DL16-4*** | | 203.81 | 203.6-204.2 | 4.98 | | -2.06 | | 4.88 |
|  |  | ***cqOC-C6-11*** | | C06 | 207.51 | | 206.5-211.6 | | 3.47 | 0.52 | | 3.18 | *13DL* | | ***qOC-13DL16-8*** | | 207.51 | 206.5-211.6 | 3.47 | | 0.52 | | 3.18 |
|  |  | ***cqOC-C8*** | | C08 | 66.15 | | 62.66 - 69.65 | | 2.03-3.75 | 0.40-0.79 | | 1.92-9.55 | *10GS/10DL* | | ***qOC-10GS18*** | | 65.29 | 58.4-67.7 | 3.75 | | 0.79 | | 9.55 |
|  |  |  |  |  |  |  |  |  |  |  |  |  |  |  | ***qOC-10DL18*** | | 67.29 | 56.9-67.5 | 2.03 | | 0.40 | | 1.92 |
|  |  | ***cqOC-C9-1*** | | C09 | 7.39 | | 4.08 - 10.7 | | 2.01-6.41 | 0.45-0.68 | | 1.98-6.49 | *12WH/10DL/11DL/12DL/09DL* | | ***qOC-12WH19*** | | 5.61 | 0-21.2 | 2.01 | | 0.45 | | 2.12 |
|  |  |  |  |  |  |  |  |  |  |  |  |  |  |  | ***qOC-10DL19*** | | 6.71 | 4.3-14.4 | 5.35 | | 0.68 | | 5.36 |
|  |  |  |  |  |  |  |  |  |  |  |  |  |  |  | ***qOC-11DL19*** | | 6.71 | 0-21.2 | 2.08 | | 0.46 | | 1.98 |
|  |  |  |  |  |  |  |  |  |  |  |  |  |  |  | ***qOC-12DL19*** | | 6.71 | 0-16.4 | 3.34 | | 0.49 | | 3.00 |
|  |  |  |  |  |  |  |  |  |  |  |  |  |  |  | ***qOC-09DL19*** | | 10.41 | 1.5-15.8 | 6.41 | | 0.61 | | 6.49 |
|  |  | ***cqOC-C9-2*** | | C09 | 27.11 | | 24.6-29.8 | | 3.81 | 0.54 | | 3.70 | *13DL* | | ***qOC-13DL19*** | | 27.11 | 24.6-29.8 | 3.81 | | 0.54 | | 3.70 |
|  | **SPC** | ***cqPC-A2*** | | A02 | 0.01 | | 0 - 1.13 | | 2.91-4.83 | 0.25-0.47 | | 3.42-10.09 | 11GS/12DL | | ***qPC-11GS2*** | | 0.01 | 0-2.5 | 4.83 | | 0.47 | | 10.09 |
|  |  |  |  |  |  |  |  |  |  |  |  |  |  |  | ***qPC-12DL2*** | | 0.01 | 0-5.2 | 2.91 | | 0.25 | | 3.42 |
|  |  | ***cqPC-A3-1*** | | A03 | 127.01 | | 124.80-127.70 | | 3.46 | -0.34 | | 4.23 | 11WH | | ***qPC-11WH3-1*** | | 127.01 | 124.8-127.7 | 3.46 | | -0.35 | | 4.23 |
|  |  | ***cqPC-A3-2*** | | A03 | 131.81 | | 131.30-132.30 | | 8.7 | -2.12 | | 27.48 | 10GS | | ***qPC-10GS3-1*** | | 131.81 | 131.3-132.3 | 8.70 | | -2.13 | | 27.48 |
|  |  | ***cqPC-A3-3*** | | A03 | 135.51 | | 133.00-139.90 | | 4.64 | -0.4 | | 5.68 | 11WH | | ***qPC-11WH3-2*** | | 135.51 | 133-139.9 | 4.64 | | -0.40 | | 5.68 |
|  |  | ***cqPC-A3-4*** | | A03 | 203.51 | | 200.58 - 206.43 | | 2.11-7.24 | -0.22--0.58 | | 2.65-7.75 | 12DL/13DL/13WH | | ***qPC-12DL3*** | | 203.51 | 201.7-209.6 | 2.38 | | -0.22 | | 2.79 |
|  |  |  |  |  |  |  |  |  |  |  |  |  |  |  | ***qPC-13DL3*** | | 203.51 | 197.7-207.1 | 7.24 | | -0.36 | | 7.75 |
|  |  |  |  |  |  |  |  |  |  |  |  |  |  |  | ***qPC-13WH3*** | | 203.51 | 192-214.9 | 2.11 | | -0.58 | | 2.65 |
|  |  | ***cqPC-A3-5*** | | A03 | 216.61 | | 214.60-216.90 | | 6.64 | 1.95 | | 20.53 | 10GS | | ***qPC-10GS3-2*** | | 216.61 | 214.6-216.9 | 6.64 | | 1.95 | | 20.53 |
|  |  | ***cqPC-A4-1*** | | A04 | 14.59 | | 13.00-15.50 | | 3.74 | -0.44 | | 7.65 | 11GS | | ***qPC-11GS4*** | | 14.59 | 13-15.5 | 3.74 | | -0.44 | | 7.65 |
|  |  | ***cqPC-A4-2*** | | A04 | 55.29 | | 52.50-56.40 | | 4.7 | -0.53 | | 6.19 | 12WH | | ***qPC-12WH4*** | | 55.29 | 52.5-56.4 | 4.70 | | -0.53 | | 6.19 |
|  |  | ***cqPC-A7*** | | A07 | 97.71 | | 95.80-100.90 | | 4 | -1.01 | | 4.89 | 09DL | | ***qPC-09DL7*** | | 97.71 | 95.8-100.9 | 4.01 | | -1.01 | | 4.89 |
|  |  | ***cqPC-A9-1*** | | A09 | 1.17 | | 0 - 5.36 | | 2.10-4.42 | -0.27--0.32 | | 2.53-5.23 | 10DL/12DL | | ***qPC-10DL9-1*** | | 0.71 | 0-12.1 | 2.10 | | -0.27 | | 2.53 |
|  |  |  |  |  |  |  |  |  |  |  |  |  |  |  | ***qPC-12DL9*** | | 1.61 | 0-11.6 | 4.42 | | -0.32 | | 5.23 |
|  |  | ***cqPC-A9-2*** | | A09 | 16.4 | | 13.47 - 19.32 | | 2.93-4.15 | -0.31--0.53 | | 3.82-5.39 | 12WH/10HG/11DL | | ***qPC-12WH9*** | | 16.31 | 2.5-21.2 | 2.93 | | -0.31 | | 3.82 |
|  |  |  |  |  |  |  |  |  |  |  |  |  |  |  | ***qPC-10HG9-1*** | | 16.41 | 13-19.6 | 3.05 | | -0.31 | | 3.90 |
|  |  |  |  |  |  |  |  |  |  |  |  |  |  |  | ***qPC-11DL9-1*** | | 16.41 | 2.5-19.6 | 4.15 | | -0.53 | | 5.39 |
|  |  | ***cqPC-A9-3*** | | A09 | 23.89 | | 20.59 - 27.18 | | 2.14-4.19895 | -0.26--0.53 | | 2.57-5.44 | 10DL/11DL | | ***qPC-10DL9-2*** | | 21.21 | 12.1-28.5 | 2.14 | | -0.26 | | 2.57 |
|  |  |  |  |  |  |  |  |  |  |  |  |  |  |  | ***qPC-11DL9-2*** | | 24.41 | 19.6-26.8 | 4.20 | | -0.53 | | 5.44 |
|  |  | ***cqPC-A9-4*** | | A09 | 32.01 | | 31.20-33.40 | | 4.42 | -0.38 | | 5.34 | 11WH | | ***qPC-11WH9-1*** | | 32.01 | 31.2-33.4 | 4.42 | | -0.38 | | 5.35 |
|  |  | ***cqPC-A9-5*** | | *A09* | *37.31* | | *33.4 - 42.21* | | *3.04-4.42* | *-0.22--0.38* | | *3.01-5.34* | *11WH/13DL* | | ***qPC-11WH9-2*** | | 37.31 | 33.4-40.8 | 4.42 | | -0.38 | | 5.35 |
|  |  |  |  |  |  |  |  |  |  |  |  |  |  |  | ***qPC-13DL9*** | | 37.31 | 33.4-42.9 | 3.04 | | -0.23 | | 3.01 |
|  |  | ***cqPC-C1-1*** | | C01 | 93.79 | | 93.05 - 94.53 | | 2.20-4.58 | -0.19--0.39 | | 2.18-5.86 | 13DL/10DL | | ***qPC-13DL11*** | | 93.11 | 86.3-97.1 | 2.20 | | -0.19 | | 2.19 |
|  |  |  |  |  |  |  |  |  |  |  |  |  |  |  | ***qPC-10DL11*** | | 93.81 | 93.1-94.6 | 4.58 | | -0.39 | | 5.86 |
|  |  | ***cqPC-C1-2*** | | C01 | 152.19 | | 150.05 - 154.33 | | 4.02-8.22 | 0.38-1.66 | | 5.20-10.36 | 10HG/09DL/13WH | | ***qPC-10HG11*** | | 140.01 | 124.9-151.8 | 4.14 | | 0.38 | | 5.80 |
|  |  |  |  |  |  |  |  |  |  |  |  |  |  |  | ***qPC-09DL11-1*** | | 152.51 | 149-153.7 | 8.22 | | 1.66 | | 10.36 |
|  |  |  |  |  |  |  |  |  |  |  |  |  |  |  | ***qPC-13WH11-1*** | | 152.51 | 143.4-154.7 | 4.02 | | 0.90 | | 5.20 |
|  |  | ***cqPC-C1-3*** | | C01 | 159.36 | | 158.0 0- 160.72 | | 7.80-4.88 | 0.96-1.59 | | 6.26-9.86 | 09DL/13WH | | ***qPC-09DL11-2*** | | 159.21 | 157.1-160.1 | 7.80 | | 1.59 | | 9.87 |
|  |  |  |  |  |  |  |  |  |  |  |  |  |  |  | ***qPC-13WH11-2*** | | 160.11 | 157.8-164.3 | 4.88 | | 0.96 | | 6.26 |
|  |  | ***cqPC-C1-4*** | | C01 | 168.01 | | 166.00-176.90 | | 3.67 | 0.85 | | 4.78 | 13WH | | ***qPC-13WH11-3*** | | 168.01 | 166-176.9 | 3.67 | | 0.85 | | 4.78 |
|  |  | ***cqPC-C3-1*** | | C03 | 287.81 | | 287.50-288.40 | | 3.44 | -0.35 | | 4.48 | 11WH | | ***qPC-11WH13-1*** | | 287.81 | 287.5-288.4 | 3.44 | | -0.35 | | 4.48 |
|  |  | ***cqPC-C3-2*** | | C03 | 298.77 | | 297.38 - 300.15 | | 7.08-8.27 | -0.39--0.49 | | 8.74-8.92 | 11WH/13DL | | ***qPC-11WH13-2*** | | 298.21 | 295.8-300.9 | 7.08 | | -0.49 | | 8.74 |
|  |  |  |  |  |  |  |  |  |  |  |  |  |  |  | ***qPC-13DL13*** | | 299.01 | 297.6-300.9 | 8.27 | | -0.39 | | 8.92 |
|  |  | ***cqPC-C3-3*** | | C03 | 303.72 | | 302.84 - 304.59 | | 3.767-8.02 | -0.35--0.53 | | 4.82-9.82 | 10HG/11WH | | ***qPC-10HG13-1*** | | 303.11 | 302.2-305.8 | 3.77 | | -0.35 | | 4.82 |
|  |  |  |  |  |  |  |  |  |  |  |  |  |  |  | ***qPC-11WH13-3*** | | 303.91 | 302.2-304.2 | 8.02 | | -0.53 | | 9.82 |
|  |  | ***cqPC-C3-4*** | | C03 | 312.51 | | 310.1-319.9 | | 3.47 | -0.34 | | 4.47 | 10HG | | ***qPC-10HG13-2*** | | 312.51 | 310.1-319.9 | 3.47 | | -0.34 | | 4.47 |
|  |  | ***cqPC-C5-1*** | | C05 | 133.89 | | 121.3-136 | | 4.42 | 1.37 | | 5.61 | 10DL | | ***qPC-10DL15-4*** | | 133.89 | 121.3-136 | 4.42 | | 1.37 | | 5.61 |
|  |  | ***cqPC-C5-2*** | | C05 | 141.96 | | 139.89 - 144.03 | | 4.22-5.33 | -0.25--0.32 | | 5.42-6.64 | 10DL/09DL | | ***qPC-10DL15-3*** | | 141.19 | 136-145.1 | 4.22 | | -0.32 | | 5.42 |
|  |  |  |  |  |  |  |  |  |  |  |  |  |  |  | ***qPC-09DL15*** | | 143.09 | 140.1-145 | 5.33 | | -0.25 | | 6.64 |
|  |  | ***cqPC-C5-3*** | | C05 | 225.49 | | 221.80-225.90 | | 5 | -0.38 | | 6.55 | 11DL | | ***qPC-11DL15-2*** | | 225.49 | 221.8-225.9 | 5.00 | | -0.38 | | 6.55 |
|  |  | ***cqPC-C5-4*** | | C05 | 231.59 | | 231.20-233.90 | | 3.63 | -0.41 | | 4.78 | 11DL | | ***qPC-11DL15-1*** | | 231.59 | 231.2-233.9 | 3.63 | | -0.41 | | 4.78 |
|  |  | ***cqPC-C5-5*** | | C05 | 237.39 | | 234.67 - 240.1 | | 2.48-4.50 | -0.29--0.6 | | 5.86-3.34 | 10DL/12DL/13DL | | ***qPC-10DL15-2*** | | 237.39 | 230.9-242.8 | 3.92 | | -0.29 | | 5.86 |
|  |  |  |  |  |  |  |  |  |  |  |  |  |  |  | ***qPC-12DL15-3*** | | 237.39 | 234.6-245.4 | 2.48 | | -0.60 | | 3.34 |
|  |  |  |  |  |  |  |  |  |  |  |  |  |  |  | ***qPC-13DL15-3*** | | 237.39 | 236-243.4 | 4.50 | | -0.38 | | 5.56 |
|  |  | ***cqPC-C5-6*** | | C05 | 252.21 | | 251.54 - 252.89 | | 2.20-7.91 | -0.24--0.88 | | 2.91-8.51 | 10DL/12DL/13WH/13DL | | ***qPC-10DL15-1*** | | 250.79 | 248.7-251.6 | 5.23 | | -0.24 | | 6.67 |
|  |  |  |  |  |  |  |  |  |  |  |  |  |  |  | ***qPC-12DL15-2*** | | 250.79 | 248-253.7 | 3.88 | | -0.30 | | 4.58 |
|  |  |  |  |  |  |  |  |  |  |  |  |  |  |  | ***qPC-13WH15-2*** | | 250.79 | 245.6-257.7 | 2.20 | | -0.72 | | 2.91 |
|  |  |  |  |  |  |  |  |  |  |  |  |  |  |  | ***qPC-13DL15-2*** | | 252.79 | 251.6-253.2 | 7.91 | | -0.88 | | 8.51 |
|  |  | ***cqPC-C5-7*** | | C05 | 260.81 | | 260.26 - 261.37 | | 2.68-5.72 | 0.38-1.7758 | | 3.41-6.25 | 12DL/13DL/13WH | | ***qPC-12DL15-1*** | | 260.59 | 259.6-261 | 3.01 | | 1.78 | | 3.57 |
|  |  |  |  |  |  |  |  |  |  |  |  |  |  |  | ***qPC-13DL15-1*** | | 261.09 | 260.1-263.2 | 5.72 | | 0.38 | | 6.25 |
|  |  |  |  |  |  |  |  |  |  |  |  |  |  |  | ***qPC-13WH15-1*** | | 261.29 | 260.9-263.2 | 2.68 | | 0.38 | | 3.41 |
|  |  | ***cqPC-C6-1*** | | C06 | 54.45 | | 50.31 - 58.58 | | 2.08-3.17 | -0.27--0.29 | | 2.39-4.03 | 12DL/11WH | | ***qPC-12DL16-1*** | | 47.91 | 41.9-56.7 | 3.17 | | -0.29 | | 4.03 |
|  |  |  |  |  |  |  |  |  |  |  |  |  |  |  | ***qPC-11WH16*** | | 56.71 | 53.1-64.2 | 2.08 | | -0.27 | | 2.39 |
|  |  | ***cqPC-C6-2*** | | C06 | 149.41 | | 145.01 - 153.8 | | 2.53-4.39 | -0.24--0.42 | | 2.87-5.59 | 10DL/12DL | | ***qPC-10DL16-1*** | | 149.41 | 142-151.9 | 4.39 | | -0.42 | | 5.59 |
|  |  |  |  |  |  |  |  |  |  |  |  |  |  |  | ***qPC-12DL16-3*** | | 149.41 | 133.6-152.8 | 2.53 | | -0.24 | | 2.87 |
|  |  | ***cqPC-C6-3*** | | C06 | 156.60 | | 153.85 - 159.35 | | 2.28-3.24 | -0.34--0.37 | | 4.31-5.84 | 10GS/10DL | | ***qPC-10GS16*** | | 154.61 | 55.9-160.6 | 2.28 | | -0.34 | | 5.85 |
|  |  |  |  |  |  |  |  |  |  |  |  |  |  |  | ***qPC-10DL16-2*** | | 156.61 | 154.6-160.1 | 3.24 | | -0.37 | | 4.31 |
|  |  | ***cqPC-C7*** | | C07 | 109.84 | | 105.01 - 114.66 | | 2.21-4.77 | -0.28--0.32 | | 2.78-5.68 | 12DL/11WH | | ***qPC-12DL17*** | | 108.61 | 103-114.6 | 4.77 | | -0.32 | | 5.68 |
|  |  |  |  |  |  |  |  |  |  |  |  |  |  |  | ***qPC-11WH17*** | | 112.61 | 97.2-114.6 | 2.21 | | -0.28 | | 2.78 |
|  |  | ***cqPC-C8*** | | C08 | 15.39 | | 11.3-16.7 | | 4.41 | 0.28 | | 4.62 | 13DL | | ***qPC-13DL18*** | | 15.39 | 11.3-16.7 | 4.41 | | 0.28 | | 4.62 |
|  |  | ***cqPC-C9-1*** | | C09 | 2.00 | | 0.0 - 6.7 | | 2.23-3.28 | -0.28--0.35 | | 2.86-4.45 | 12WH/10DL | | ***qPC-12WH19-1*** | | 2.01 | 0-6.7 | 3.28 | | -0.35 | | 4.45 |
|  |  |  |  |  |  |  |  |  |  |  |  |  |  |  | ***qPC-10DL19*** | | 5.61 | 0-8.4 | 2.23 | | -0.28 | | 2.87 |
|  |  | ***cqPC-C9-2*** | | C09 | 8.12 | | 6.47 - 9.78 | | 2.055-2.98 | -0.32--0.34 | | 3.88-4.18 | 12WH/11GS | | ***qPC-12WH19-2*** | | 8.41 | 6.7-10.4 | 2.98 | | -0.34 | | 3.88 |
|  |  |  |  |  |  |  |  |  |  |  |  |  |  |  | ***qPC-11GS19*** | | 11.81 | 1.7-17.4 | 2.05 | | -0.32 | | 4.18 |
|  |  | ***cqPC-C9-3*** | | C09 | 70.54 | | 69.35 - 71.72 | | 2.31-3.09 | -0.23--0.603 | | 2.92-3.22 | 13DL/13WH | | ***qPC-13DL19-1*** | | 70.01 | 67.7-70.5 | 3.09 | | -0.23 | | 3.22 |
|  |  |  |  |  |  |  |  |  |  |  |  |  |  |  | ***qPC-13WH19-1*** | | 71.91 | 70-74.5 | 2.31 | | -0.60 | | 2.92 |
|  |  | ***cqPC-C9-4*** | | C09 | 77.31 | | 74.86 - 79.75 | | 2.41-2.60 | -0.2--0.64 | | 2.51-3.28 | 13DL/13WH | | ***qPC-13DL19-2*** | | 77.31 | 74.7-80.3 | 2.41 | | -0.21 | | 2.51 |
|  |  |  |  |  |  |  |  |  |  |  |  |  |  |  | ***qPC-13WH19-2*** | | 77.31 | 74.5-84.6 | 2.60 | | -0.64 | | 3.28 |
| **Table S4. BSA sequencing of the two parents and two bulks.** | | | | | | | | | | |  | | |  | |  | | | |  | |  | |
| **Sample** | | | **Mapped reads** | | | **Total reads** | | **Mapping rate (%)** | | | **Average depth (X)** | | | **Coverage at least 1X (%)** | | **Coverage at least 4X (%)** | | | | **SNP** | | **InDel** | |
| Ken-C8 | | | 93,932,387 | | | 96,369,652 | | 97.47 | | | 17.16 | | | 89.31 | | 78.2 | | | | 2,695,926 | | 756,361 | |
| N53-2 | | | 111,304,287 | | | 115,268,130 | | 96.56 | | | 19.82 | | | 91.99 | | 84.07 | | | |  |  |  |  |
| LOW | | | 231,057,024 | | | 274,084,570 | | 84.3 | | | 35.85 | | | 97.93 | | 93.72 | | | |  | |  | |
| HIGH | | | 249,399,218 | | | 274,853,538 | | 90.74 | | | 39 | | | 95.99 | | 91.95 | | | |  | |  | |

| **Table S5. AGRs identified underlying SOC based on BSA.** | | | |
| --- | --- | --- | --- |
| **AGR** | **Chromosome** | **Start (Mb)** | **End (Mb)** |
| ***AGR_A2-1*** | chrA02 | 3.249 | 3.250 |
| ***AGR_A2-2*** | chrA02 | 3.293 | 3.321 |
| ***AGR_A2-3*** | chrA02 | 3.324 | 3.333 |
| ***AGR_A2-4*** | chrA02 | 3.615 | 3.626 |
| ***AGR_A2-5*** | chrA02 | 3.645 | 3.653 |
| ***AGR_A3-1*** | chrA03 | 14.362 | 14.365 |
| ***AGR_A3-2*** | chrA03 | 14.369 | 14.386 |
| ***AGR_A3-3*** | chrA03 | 14.451 | 14.456 |
| ***AGR_A3-4*** | chrA03 | 14.459 | 14.499 |
| ***AGR_A3-5*** | chrA03 | 14.508 | 14.627 |
| ***AGR_A3-6*** | chrA03 | 17.160 | 17.362 |
| ***AGR_A3-7*** | chrA03 | 17.363 | 22.486 |
| ***AGR_A4-1*** | chrA04 | 5.459 | 5.488 |
| ***AGR_A4-2*** | chrA04 | 19.141 | 19.148 |
| ***AGR_A5-1*** | chrA05 | 2.377 | 7.491 |
| ***AGR_A5-2*** | chrA05 | 19.787 | 19.838 |
| ***AGR_A5-3*** | chrA05 | 20.379 | 20.384 |
| ***AGR_A5-4*** | chrA05 | 20.512 | 20.553 |
| ***AGR_A6-1*** | chrA06 | 24.099 | 24.371 |
| ***AGR_A7-1*** | chrA07 | 21.229 | 21.233 |
| ***AGR_A7-2*** | chrA07 | 21.235 | 22.451 |
| ***AGR_A7-3*** | chrA07 | 22.452 | 22.454 |
| ***AGR_A8-1*** | chrA08 | 0.832 | 2.843 |
| ***AGR_A8-2*** | chrA08 | 2.846 | 2.849 |
| ***AGR_A8-3*** | chrA08 | 2.914 | 2.916 |
| ***AGR_A8-4*** | chrA08 | 7.150 | 13.068 |
| ***AGR_A9-1*** | chrA09 | 22.259 | 22.688 |
| ***AGR_A9-2*** | chrA09 | 22.728 | 22.729 |
| ***AGR_A9-3*** | chrA09 | 22.739 | 22.748 |
| ***AGR_A9-4*** | chrA09 | 22.890 | 23.336 |
| ***AGR_A9-5*** | chrA09 | 23.341 | 23.342 |
| ***AGR_A9-6*** | chrA09 | 29.687 | 29.688 |
| ***AGR_A9-7*** | chrA09 | 29.691 | 29.728 |
| ***AGR_A9-8*** | chrA09 | 29.774 | 31.256 |
| ***AGR_A9-9*** | chrA09 | 31.292 | 31.298 |
| ***AGR_A9-10*** | chrA09 | 31.320 | 31.410 |
| ***AGR_A9-11*** | chrA09 | 31.519 | 31.610 |
| ***AGR_A9-12*** | chrA09 | 31.615 | 33.858 |
| ***AGR_A10*** | chrA10 | 17.277 | 17.395 |
| ***AGR_C1-1*** | chrC01 | 1.405 | 1.409 |
| ***AGR_C1-2*** | chrC01 | 1.411 | 1.412 |
| ***AGR_C1-3*** | chrC01 | 1.441 | 1.603 |
| ***AGR_C1-4*** | chrC01 | 1.612 | 1.619 |
| ***AGR_C1-5*** | chrC01 | 1.621 | 1.622 |
| ***AGR_C1-6*** | chrC01 | 1.623 | 1.657 |
| ***AGR_C1-7*** | chrC01 | 1.772 | 1.790 |
| ***AGR_C1-8*** | chrC01 | 1.794 | 1.798 |
| ***AGR_C1-9*** | chrC01 | 1.963 | 1.966 |
| ***AGR_C1-10*** | chrC01 | 1.967 | 1.968 |
| ***AGR_C1-11*** | chrC01 | 1.969 | 1.973 |
| ***AGR_C1-12*** | chrC01 | 1.974 | 1.983 |
| ***AGR_C1-13*** | chrC01 | 10.130 | 10.131 |
| ***AGR_C1-14*** | chrC01 | 10.132 | 10.630 |
| ***AGR_C2-1*** | chrC02 | 5.642 | 6.394 |
| ***AGR_C2-2*** | chrC02 | 6.395 | 6.396 |
| ***AGR_C2-3*** | chrC02 | 6.404 | 6.437 |
| ***AGR_C2-4*** | chrC02 | 10.793 | 10.794 |
| ***AGR_C2-5*** | chrC02 | 29.136 | 29.414 |
| ***AGR_C2-6*** | chrC02 | 41.642 | 41.946 |
| ***AGR_C2-7*** | chrC02 | 46.159 | 46.196 |
| ***AGR_C3-1*** | chrC03 | 2.935 | 3.021 |
| ***AGR_C3-2*** | chrC03 | 3.034 | 3.058 |
| ***AGR_C3-3*** | chrC03 | 3.081 | 4.750 |
| ***AGR_C3-4*** | chrC03 | 4.760 | 4.761 |
| ***AGR_C3-5*** | chrC03 | 6.241 | 6.256 |
| ***AGR_C3-6*** | chrC03 | 6.263 | 6.729 |
| ***AGR_C3-7*** | chrC03 | 9.097 | 10.565 |
| ***AGR_C3-8*** | chrC03 | 10.593 | 11.053 |
| ***AGR_C3-9*** | chrC03 | 12.077 | 12.887 |
| ***AGR_C3-10*** | chrC03 | 14.076 | 14.966 |
| ***AGR_C3-11*** | chrC03 | 36.502 | 36.516 |
| ***AGR_C3-12*** | chrC03 | 36.518 | 42.371 |
| ***AGR_C3-13*** | chrC03 | 42.380 | 42.382 |
| ***AGR_C3-14*** | chrC03 | 46.541 | 46.807 |
| ***AGR_C3-15*** | chrC03 | 46.810 | 46.816 |
| ***AGR_C3-16*** | chrC03 | 48.960 | 56.668 |
| ***AGR_C3-17*** | chrC03 | 60.530 | 60.536 |
| ***AGR_C3-18*** | chrC03 | 60.543 | 60.544 |
| ***AGR_C4-1*** | chrC04 | 5.509 | 6.508 |
| ***AGR_C4-2*** | chrC04 | 8.433 | 8.829 |
| ***AGR_C4-3*** | chrC04 | 8.830 | 8.834 |
| ***AGR_C4-4*** | chrC04 | 48.350 | 48.863 |
| ***AGR_C4-5*** | chrC04 | 48.865 | 48.929 |
| ***AGR_C5-1*** | chrC05 | 3.140 | 3.660 |
| ***AGR_C5-2*** | chrC05 | 3.853 | 3.854 |
| ***AGR_C5-3*** | chrC05 | 3.855 | 4.891 |
| ***AGR_C5-4*** | chrC05 | 7.285 | 7.287 |
| ***AGR_C5-5*** | chrC05 | 7.288 | 7.290 |
| ***AGR_C5-6*** | chrC05 | 7.291 | 8.512 |
| ***AGR_C5-7*** | chrC05 | 11.386 | 13.061 |
| ***AGR_C5-8*** | chrC05 | 13.080 | 13.081 |
| ***AGR_C5-9*** | chrC05 | 13.675 | 15.006 |
| ***AGR_C5-10*** | chrC05 | 15.012 | 15.020 |
| ***AGR_C5-11*** | chrC05 | 15.433 | 15.465 |
| ***AGR_C5-12*** | chrC05 | 15.536 | 15.584 |
| ***AGR_C5-13*** | chrC05 | 15.685 | 15.692 |
| ***AGR_C5-14*** | chrC05 | 15.855 | 15.962 |
| ***AGR_C5-15*** | chrC05 | 16.123 | 16.170 |
| ***AGR_C5-16*** | chrC05 | 16.401 | 16.504 |
| ***AGR_C5-17*** | chrC05 | 16.533 | 16.536 |
| ***AGR_C5-18*** | chrC05 | 16.584 | 17.709 |
| ***AGR_C5-19*** | chrC05 | 18.281 | 18.349 |
| ***AGR_C5-20*** | chrC05 | 19.406 | 20.556 |
| ***AGR_C5-21*** | chrC05 | 20.574 | 20.595 |
| ***AGR_C5-22*** | chrC05 | 20.771 | 20.777 |
| ***AGR_C5-23*** | chrC05 | 21.556 | 21.608 |
| ***AGR_C5-24*** | chrC05 | 21.631 | 22.027 |
| ***AGR_C5-25*** | chrC05 | 24.461 | 25.404 |
| ***AGR_C5-26*** | chrC05 | 25.820 | 25.945 |
| ***AGR_C5-27*** | chrC05 | 25.964 | 26.860 |
| ***AGR_C5-28*** | chrC05 | 26.866 | 26.937 |
| ***AGR_C5-29*** | chrC05 | 28.637 | 30.840 |
| ***AGR_C5-30*** | chrC05 | 30.842 | 30.845 |
| ***AGR_C5-31*** | chrC05 | 30.846 | 32.910 |
| ***AGR_C5-32*** | chrC05 | 33.990 | 37.061 |
| ***AGR_C5-33*** | chrC05 | 37.312 | 40.210 |
| ***AGR_C6-1*** | chrC06 | 15.773 | 15.774 |
| ***AGR_C6-2*** | chrC06 | 15.775 | 16.517 |
| ***AGR_C6-3*** | chrC06 | 18.873 | 18.874 |
| ***AGR_C6-4*** | chrC06 | 19.024 | 19.025 |
| ***AGR_C8-1*** | chrC08 | 35.800 | 36.357 |
| ***AGR_C8-2*** | chrC08 | 36.398 | 36.569 |
| ***AGR_C8-3*** | chrC08 | 36.584 | 36.587 |
| ***AGR_C8-4*** | chrC08 | 36.685 | 36.687 |
| ***AGR_C8-5*** | chrC08 | 36.920 | 36.978 |
| ***AGR_C8-6*** | chrC08 | 36.980 | 37.014 |
| ***AGR_C8-7*** | chrC08 | 37.028 | 37.087 |
| ***AGR_C8-8*** | chrC08 | 37.088 | 37.109 |
| ***AGR_C8-9*** | chrC08 | 37.175 | 38.179 |
| ***AGR_C9-1*** | chrC09 | 42.566 | 42.570 |
| ***AGR_C9-2*** | chrC09 | 45.222 | 45.223 |
| ***AGR_C9-3*** | chrC09 | 45.766 | 45.783 |
| ***AGR_C9-4*** | chrC09 | 46.554 | 46.766 |
| ***AGR_Am-1*** | chrA05_random | 0.000 | 0.717 |
| ***AGR_Am-2*** | chrA06_random | 1.780 | 1.886 |
| ***AGR_Am-3*** | chrA06_random | 1.914 | 2.186 |
| ***AGR_Am-4*** | chrA08_random | 0.000 | 1.678 |
| ***AGR_Am-5*** | chrA08_random | 1.679 | 1.680 |
| ***AGR_Am-6*** | chrA09_random | 3.327 | 4.091 |
| ***AGR_Am-7*** | chrA10_random | 2.232 | 2.263 |
| ***AGR_Am-8*** | chrC02_random | 2.850 | 2.861 |
| ***AGR_Am-9*** | chrC03_random | 2.588 | 5.530 |
| ***AGR_Am-10*** | chrC05_random | 1.670 | 1.734 |
| ***AGR_Am-11*** | chrC05_random | 1.736 | 1.749 |
| ***AGR_Am-12*** | chrC05_random | 1.774 | 2.051 |
| ***AGR_Am-13*** | chrC05_random | 2.268 | 3.271 |
| ***AGR_Am-14*** | chrC05_random | 3.529 | 3.702 |
| ***AGR_Am-15*** | chrC08_random | 4.336 | 4.337 |
| ***AGR_Am-16*** | chrC08_random | 4.339 | 4.367 |
| ***AGR_Am-17*** | chrAnn_random | 9.186 | 9.363 |
| ***AGR_Am-18*** | chrAnn_random | 9.534 | 9.554 |
| ***AGR_Am-19*** | chrAnn_random | 19.901 | 20.339 |
| ***AGR_Cm-1*** | chrCnn_random | 5.609 | 6.020 |
| ***AGR_Cm-2*** | chrCnn_random | 11.200 | 11.374 |
| ***AGR_Cm-3*** | chrCnn_random | 29.022 | 29.697 |
| ***AGR_Cm-4*** | chrUnn_random | 2.369 | 2.448 |
| ***AGR_Cm-5*** | chrUnn_random | 8.177 | 8.296 |

| **Table S6. AGRs overlapping with SOC-QTLs from the QTL mapping method based on a high-density genetic linkage map.** | | | | | | | |  |
| --- | --- | --- | --- | --- | --- | --- | --- | --- |
| **BSA** | | | | **QTL mapping** | | | | |
| **AGR** | **Chr.** | **Start (Mb)** | **End (Mb)** | **Consensus QTL** | **Genomic region (Mb)** | **Chromosome** | **Confidence interval (cM)** | **Marker interval** |
|  |  |  |  | ***cqOC-A1*** | 6.016 - 8.053 | A01 | 104.00-112.10 | Bn_A01_p0091 ~ Bn_A01_p0189_A1 |
|  |  |  |  | ***cqOC-A2-1*** |  | A02 | 0 - 7.08 | B060E11-1b ~ B021E05-2-A2 |
|  |  |  |  | ***cqOC-A2-2*** | 6.483 - 7.642 | A02 | 105.80-109.20 | Bn_A02_p0209_A2 ~ Bns_p2186_A2 |
|  |  |  |  | ***cqOC-A2-3*** | 6.531 - 19.73 | A02 | 113.42 - 119.10 | Bn_A02_p0326_A2 ~ Bn_A02_p0407_A2 |
|  |  |  |  | ***cqOC-A3-1*** | 3.364 - 4.284 | A03 | 23.58 - 28.43 | Bn_A03_p0733_A3 ~ Bn_A03_p0476_A3 |
|  |  |  |  | ***cqOC-A3-2*** | 4.651 - 5.386 | A03 | 31.9-35.8 | Bn_A03_p0707_A3 ~ Bn_A03_p0446_A3 |
| ***AGR_A3-1*** | chrA03 | 14.362 | 14.365 | ***cqOC-A3-3*** | 14.32 - 16.07 | A03 | 120.45 - 124.29 | Bns_p2873 ~ Bn_A03_p0721_A3 |
| ***AGR_A3-2*** | chrA03 | 14.369 | 14.386 |  |  |  |  |  |
| ***AGR_A3-3*** | chrA03 | 14.451 | 14.456 |  |  |  |  |  |
| ***AGR_A3-4*** | chrA03 | 14.459 | 14.499 |  |  |  |  |  |
| ***AGR_A3-5*** | chrA03 | 14.508 | 14.627 |  |  |  |  |  |
| ***AGR_A3-7*** | chrA03 | 17.363 | 22.486 | ***cqOC-A3-4*** | 21.36 - 21.86 | A03 | 150.70-153.90 | Bn_A03_p0586 ~ Bns_p3760_A3 |
|  |  |  |  | ***cqOC-A3-5*** | 22.1 - 23.15 | A03 | 182.10 - 183.59 | Bn_A03_p0699 ~ Bns_p2343_A3 |
|  |  |  |  | ***cqOC-A3-6*** |  | A03 | 183.20-195.70 | Bn_A03_p0460_A3 ~ CNU370a |
|  |  |  |  | ***cqOC-A3-7*** |  | A03 | 200.26 - 208.03 | CNU370a ~ BRAS010-N4/N9 |
| ***AGR_A8-1*** | chrA08 | 0.832 | 2.843 | ***cqOC-A8-1*** | 2.022 - 2.088 | A08 | 1.08 - 1.85 | Bn_A08_p1603_A8 ~ Bn_A08_p1660 |
| ***AGR_A8-4*** | chrA08 | 7.15 | 13.068 | ***cqOC-A8-2*** | 9.198 - 10.83 | A08 | 7.34 - 9.88 | Bns_p2739_A8 ~ Bns_p2735_A8 |
|  |  |  |  | ***cqOC-A8-3*** | 10.37 - 12.21 | A08 | 10.85 - 13.78 | Bns_p2735_A8 ~ Bn_A08_p1589_A8 |
|  |  |  |  | ***cqOC-A8-4*** | 11.85 - 12.21 | A08 | 17.61 - 18.41 | Bn_A08_p1586_A8 ~ Bn_A08_p1618_A8 |
|  |  |  |  | ***cqOC-A8-5*** | 11.06 - 11.9 | A08 | 19.53 - 20.49 | Bn_A08_p1619 ~ Bn_A08_p1587_A8 |
|  |  |  |  | ***cqOC-A8-6*** | 11.96 - 12.81 | A08 | 20.4-24.2 | Bn_A08_p1585_A8 ~ Bn_A08_p1590 |
|  |  |  |  | ***cqOC-A9-1*** |  | A09 | 0.19 - 2.14 | Bns_p2294_A9 ~ Bn_A01_p0042_A9 |
|  |  |  |  | ***cqOC-A9-2*** | 3.264 - 4.344 | A09 | 21.81 - 24.73 | Bns_p2779_A9 ~ Bn_A09_p1846_A9 |
|  |  |  |  | ***cqOC-A9-3*** | 5.878 - 6.354 | A09 | 30.4-32.2 | Bn_A09_p1750 ~ Bn_A09_p1805 |
|  |  |  |  | ***cqOC-A9-4*** | 6.354 - 9.293 | A09 | 32.36 - 36.17 | Bn_A09_p1805 ~ Bn_A09_p1823_A9 |
|  |  |  |  | ***cqOC-A9-5*** | 24.78 - 26 | A09 | 67.1-73.1 | Bn_A05_p0981_A9 ~ Bn_A09_p1694_A9 |
|  |  |  |  | ***cqOC-A9-6*** | 28.29 - 28.49 | A09 | 94.9-96.7 | Bn_A09_p1813_A9 ~ Bn_A09_p1743_A9 |
|  |  |  |  | ***cqOC-A9-7*** | 28.49 - 28.65 | A09 | 97.23 - 99.58 | Bn_A09_p1743_A9 ~ Bn_A09_p1746 |
|  |  |  |  | ***cqOC-A9-8*** | 28.68 - 29.66 | A09 | 99.9-101.2 | Bn_A09_p1700 ~ Bn_A09_p1713_A9 |
| ***AGR_A9-8*** | chrA09 | 29.774 | 31.256 | ***cqOC-A9-9*** | 30.61 - 30.68 | A09 | 105.79 - 106.77 | Bns_p2574_A9 ~ Bn_A09_p1830_A9 |
| ***AGR_A9-9*** | chrA09 | 31.292 | 31.298 | ***cqOC-A9-10*** | 31.09 - 33.75 | A09 | 111.62 - 113.79 | Bns_p2570 ~ Bn_A09_p1707_A9 |
| ***AGR_A9-10*** | chrA09 | 31.320 | 31.410 |  |  |  |  |  |
| ***AGR_A9-11*** | chrA09 | 31.519 | 31.610 |  |  |  |  |  |
| ***AGR_A9-12*** | chrA09 | 31.615 | 33.858 |  |  |  |  |  |
|  |  |  |  | ***cqOC-A9-11*** | 33.75 - 33.83 | A09 | 116.16 - 116.59 | Bn_A09_p1705_A9 ~ Bn_A09_p1706_A9 |
|  |  |  |  | ***cqOC-A9-12*** | 32.53 - 33.83 | A09 | 119.88 - 124.13 | Bn_A09_p1888 ~ Bn_A09_p1885_A9 |
|  |  |  |  | ***cqOC-A10-1*** | 13.91 - 15.49 | A10 | 0 - 2.59 | Bn_A10_p1926 ~ Bn_A10_p2015 |
|  |  |  |  | ***cqOC-A10-2*** | 0.821 - 13.91 | A10 | 5.8-16.7 | Bn_A10_p2015 ~ niab144-A10 |
|  |  |  |  | ***cqOC-A10-3*** |  | A10 | 14.35 - 21.71 | Bn_A10_p1996_A10 ~ BnGMS625-A10/C9 |
|  |  |  |  | ***cqOC-C2-1*** | 37.24 - 39.02 | C02 | 82.1 - 86.18 | Bn_C_p2030_C2 ~ Bns_p2108_C2 |
| ***AGR_C2-6*** | chrC02 | 41.642 | 41.946 | ***cqOC-C2-2*** | 39 - 41.76 | C02 | 89.17 - 93.04 | Bns_p2329_C2 ~ Bns_p3492_C2 |
|  |  |  |  | ***cqOC-C2-3*** | 40.35 - 43.54 | C02 | 97.6-103.1 | Bns_p2762 ~ Bns_p2933 |
|  |  |  |  | ***cqOC-C2-4*** | 43.78 - 44.13 | C02 | 109.45 - 112.72 | Bns_p2945_C2 ~ Bn_A02_p0334_C2 |
|  |  |  |  | ***cqOC-C3-1*** | 0.079 - 1.592 | C03 | 142.23 - 147.64 | Bn_A03_p0486_C3 ~ Bns_p2627_C3 |
| ***AGR_C3-1*** | chrC03 | 2.935 | 3.021 | ***cqOC-C3-2*** | 2.63 - 3.386 | C03 | 163.56 - 168.65 | Bns_p3685 ~ Bns_p3238 |
| ***AGR_C3-2*** | chrC03 | 3.034 | 3.058 |  |  |  |  |  |
| ***AGR_C3-3*** | chrC03 | 3.081 | 4.750 |  |  |  |  |  |
|  |  |  |  | ***cqOC-C3-3*** | 4.06 - 5.014 | C03 | 171.6-173.9 | Bn_A03_p0598_C3 ~ Bns_p3459_C3 |
| ***AGR_C3-4*** | chrC03 | 4.760 | 4.761 |  |  |  |  |  |
| ***AGR_C3-16*** | chrC03 | 48.960 | 56.668 | ***cqOC-C3-4*** | 52.35 - 52.62 | C03 | 299.84 - 301.12 | Bns_p2064_C3 ~ Bns_p3674 |
|  |  |  |  | ***cqOC-C3-5*** | 53.83 - 55.41 | C03 | 308.17 - 309.86 | Bns_p2851 ~ Bns_p2708_C3 |
|  |  |  |  | ***cqOC-C3-6*** | 55.83 - 56.37 | C03 | 311.35 - 313.31 | Bns_p2190 ~ Bns_p2181_C3 |
|  |  |  |  | ***cqOC-C3-7*** | 56.37 - 58.52 | C03 | 314.17 - 322.64 | Bns_p2183_C3 ~ Bns_p3245_C3 |
|  |  |  |  | ***cqOC-C5-1*** | 40.06 - 0 | C05 | 58.2-85.8 | Bns_p2840_C5 ~ niab038-A9 |
|  |  |  |  | ***cqOC-C5-2*** | 40.06 - 0 | C05 | 123.4-136 | Na12G12 ~ Bns_p2550 |
|  |  |  |  | ***cqOC-C5-3*** | 9.283 - 11.18 | C05 | 181.38 - 184.99 | Bns_p3498_C5 ~ Bns_p2759_C5 |
| ***AGR_C5-9*** | chrC05 | 13.675 | 15.006 | ***cqOC-C5-4*** | 13.29 - 21.77 | C05 | 190.1 - 192.06 | Bn_A09_p1842_C5 ~ Bns_p3562_C5 |
| ***AGR_C5-10*** | chrC05 | 15.012 | 15.020 |  |  |  |  |  |
| ***AGR_C5-11*** | chrC05 | 15.433 | 15.465 |  |  |  |  |  |
| ***AGR_C5-12*** | chrC05 | 15.536 | 15.584 |  |  |  |  |  |
| ***AGR_C5-13*** | chrC05 | 15.685 | 15.692 |  |  |  |  |  |
| ***AGR_C5-14*** | chrC05 | 15.855 | 15.962 |  |  |  |  |  |
| ***AGR_C5-15*** | chrC05 | 16.123 | 16.170 |  |  |  |  |  |
| ***AGR_C5-16*** | chrC05 | 16.401 | 16.504 |  |  |  |  |  |
| ***AGR_C5-17*** | chrC05 | 16.533 | 16.536 |  |  |  |  |  |
| ***AGR_C5-18*** | chrC05 | 16.584 | 17.709 |  |  |  |  |  |
| ***AGR_C5-19*** | chrC05 | 18.281 | 18.349 |  |  |  |  |  |
| ***AGR_C5-20*** | chrC05 | 19.406 | 20.556 |  |  |  |  |  |
| ***AGR_C5-21*** | chrC05 | 20.574 | 20.595 |  |  |  |  |  |
| ***AGR_C5-22*** | chrC05 | 20.771 | 20.777 |  |  |  |  |  |
| ***AGR_C5-23*** | chrC05 | 21.556 | 21.608 |  |  |  |  |  |
| ***AGR_C5-24*** | chrC05 | 21.631 | 22.027 |  |  |  |  |  |
| ***AGR_C5-33*** | chrC05 | 37.312 | 40.210 | ***cqOC-C5-5*** | 39.46 - 40.1 | C05 | 219.85 - 220.98 | B087P06-1-A5 ~ Bns_p2824_C5 |
|  |  |  |  | ***cqOC-C5-6*** |  | C05 | 229.22 - 231.26 | Bns_p2834_C5 ~ Bns_p2844 |
|  |  |  |  | ***cqOC-C5-7*** | 40.26 - 42.76 | C05 | 232.79 - 235.24 | Bns_p2843 ~ Bns_p2845 |
|  |  |  |  | ***cqOC-C5-8*** | 40.35 - 42.76 | C05 | 234.66 - 240.11 | Bns_p2848 ~ Bn_A02_p0315_C5 |
|  |  |  |  | ***cqOC-C5-9*** | 41.02 - 41.34 | C05 | 249.56 - 252.01 | Bns_p3552_C5 ~ Bns_p3066 |
|  |  |  |  | ***cqOC-C5-10*** | 41.75 - 42.6 | C05 | 257.20-259.40 | Bns_p3351_C5 ~ Bn_A05_p1054 |
|  |  |  |  | ***cqOC-C6-1*** |  | C06 | 15.25 - 32.72 | Bn_A07_p1388 ~ Bn_A01_p0157_C6 |
| ***AGR_C6-3*** | chrC06 | 18.873 | 18.874 | ***cqOC-C6-2*** | 18.72 - 21.8 | C06 | 147.65 - 151.02 | Bns_p3085_C6 ~ Bns_p2208 |
| ***AGR_C6-4*** | chrC06 | 19.024 | 19.025 |  |  |  |  |  |
|  |  |  |  | ***cqOC-C6-3*** | 21.8 - 34.28 | C06 | 152.6 - 162.4 | Bns_p2208 ~ Bns_p2144 |
|  |  |  |  | ***cqOC-C6-4*** |  | C06 | 155.2-169.8 | Bns_p2201 ~ Bns_p2694 |
|  |  |  |  | ***cqOC-C6-5*** |  | C06 | 180.18 - 183.45 | Bns_p2490_C6 ~ Bns_p3615_C6 |
|  |  |  |  | ***cqOC-C6-6*** |  | C06 | 187.58 - 188.96 | Bns_p3421_C6 ~ Bns_p3657_C6 |
|  |  |  |  | ***cqOC-C6-7*** |  | C006 | 195.2-197 | Bns_p2696_C6 ~ Bns_p3315 |
|  |  |  |  | ***cqOC-C6-8*** |  | C06 | 198.88 - 199.33 | Bns_p3363 ~ Bns_p2278_C6 |
|  |  |  |  | ***cqOC-C6-9*** |  | C06 | 200.4-202.3 | Bns_p3317 ~ Bns_p3366_C6 |
|  |  |  |  | ***cqOC-C6-10*** |  | C06 | 203.56 - 204.05 | Bns_p2141 ~ Bns_p2984 |
| ***AGR_C8-1*** | chrC08 | 35.800 | 36.357 | ***cqOC-C6-11*** | 34.14 - 35.89 | C06 | 206.5-211.6 | Bns_p2985_C6 ~ Bns_p3008_C6 |
|  |  |  |  | ***cqOC-C8*** | 29.29 - 34.28 | C08 | 62.66 - 69.65 | Bns_p2503_C8 ~ Bns_p2572_C8 |
|  |  |  |  | ***cqOC-C9-1*** |  | C09 | 4.08 - 10.7 | FITO516b-N1 ~ CB10064 |
|  |  |  |  | ***cqOC-C9-2*** | 0.414 - 1.338 | C09 | 24.6-29.8 | Bns_p2914_C9 ~ Bns_p2925_C9 |

| **Table S7. Genomic regions corresponding to the confidence interval of SOC and SPC-QTLs.** | | | | |  |  |
| --- | --- | --- | --- | --- | --- | --- |
|  | **Consensus QTL** | **Chromosome** | **Confidence interval (cM)** | **Marker interval** | **Genomic region (Mb)** | **Coverage length (Mb)** |
| **SOC** | ***cqOC-A1*** | A01 | 104.00-112.10 | Bn_A01_p0091 ~ Bn_A01_p0189_A1 | 6.016 - 8.053 | 2.04 |
|  | ***cqOC-A2-1*** | A02 | 0 - 7.08 | B060E11-1b ~ B021E05-2-A2 |  |  |
|  | ***cqOC-A2-2*** | A02 | 105.80-109.20 | Bn_A02_p0209_A2 ~ Bns_p2186_A2 | 6.483 - 7.642 | 1.16 |
|  | ***cqOC-A2-3*** | A02 | 113.42 - 119.10 | Bn_A02_p0326_A2 ~ Bn_A02_p0407_A2 | 6.531 - 19.73 | 13.21 |
|  | ***cqOC-A3-1*** | A03 | 23.58 - 28.43 | Bn_A03_p0733_A3 ~ Bn_A03_p0476_A3 | 3.364 - 4.284 | 0.92 |
|  | ***cqOC-A3-2*** | A03 | 31.9-35.8 | Bn_A03_p0707_A3 ~ Bn_A03_p0446_A3 | 4.651 - 5.386 | 0.74 |
|  | ***cqOC-A3-3*** | A03 | 120.45 - 124.29 | Bns_p2873 ~ Bn_A03_p0721_A3 | 14.32 - 16.07 | 1.75 |
|  | ***cqOC-A3-4*** | A03 | 150.70-153.90 | Bn_A03_p0586 ~ Bns_p3760_A3 | 21.36 - 21.86 | 0.50 |
|  | ***cqOC-A3-5*** | A03 | 182.10 - 183.59 | Bn_A03_p0699 ~ Bns_p2343_A3 | 22.1 - 23.15 | 1.05 |
|  | ***cqOC-A3-6*** | A03 | 183.20-195.70 | Bn_A03_p0460_A3 ~ CNU370a |  |  |
|  | ***cqOC-A3-7*** | A03 | 200.26 - 208.03 | CNU370a ~ BRAS010-N4/N9 |  |  |
|  | ***cqOC-A8-1*** | A08 | 1.08 - 1.85 | Bn_A08_p1603_A8 ~ Bn_A08_p1660 | 2.022 - 2.088 | 0.07 |
|  | ***cqOC-A8-2*** | A08 | 7.34 - 9.88 | Bns_p2739_A8 ~ Bns_p2735_A8 | 9.198 - 10.83 | 1.64 |
|  | ***cqOC-A8-3*** | A08 | 10.85 - 13.78 | Bns_p2735_A8 ~ Bn_A08_p1589_A8 | 10.37 - 12.21 | 1.84 |
|  | ***cqOC-A8-4*** | A08 | 17.61 - 18.41 | Bn_A08_p1586_A8 ~ Bn_A08_p1618_A8 | 11.85 - 12.21 | 0.36 |
|  | ***cqOC-A8-5*** | A08 | 19.53 - 20.49 | Bn_A08_p1619 ~ Bn_A08_p1587_A8 | 11.06 - 11.9 | 0.84 |
|  | ***cqOC-A8-6*** | A08 | 20.4-24.2 | Bn_A08_p1585_A8 ~ Bn_A08_p1590 | 11.96 - 12.81 | 0.85 |
|  | ***cqOC-A9-1*** | A09 | 0.19 - 2.14 | Bns_p2294_A9 ~ Bn_A01_p0042_A9 |  |  |
|  | ***cqOC-A9-10*** | A09 | 111.62 - 113.79 | Bns_p2570 ~ Bn_A09_p1707_A9 | 31.09 - 33.75 | 2.66 |
|  | ***cqOC-A9-11*** | A09 | 116.16 - 116.59 | Bn_A09_p1705_A9 ~ Bn_A09_p1706_A9 | 33.75 - 33.83 | 0.08 |
|  | ***cqOC-A9-12*** | A09 | 119.88 - 124.13 | Bn_A09_p1888 ~ Bn_A09_p1885_A9 | 32.53 - 33.83 | 1.30 |
|  | ***cqOC-A9-2*** | A09 | 21.81 - 24.73 | Bns_p2779_A9 ~ Bn_A09_p1846_A9 | 3.264 - 4.344 | 1.08 |
|  | ***cqOC-A9-3*** | A09 | 30.4-32.2 | Bn_A09_p1750 ~ Bn_A09_p1805 | 5.878 - 6.354 | 0.48 |
|  | ***cqOC-A9-4*** | A09 | 32.36 - 36.17 | Bn_A09_p1805 ~ Bn_A09_p1823_A9 | 6.354 - 9.293 | 2.94 |
|  | ***cqOC-A9-5*** | A09 | 67.1-73.1 | Bn_A05_p0981_A9 ~ Bn_A09_p1694_A9 | 24.78 - 26 | 1.22 |
|  | ***cqOC-A9-6*** | A09 | 94.9-96.7 | Bn_A09_p1813_A9 ~ Bn_A09_p1743_A9 | 28.29 - 28.49 | 0.20 |
|  | ***cqOC-A9-7*** | A09 | 97.23 - 99.58 | Bn_A09_p1743_A9 ~ Bn_A09_p1746 | 28.49 - 28.65 | 0.16 |
|  | ***cqOC-A9-8*** | A09 | 99.9-101.2 | Bn_A09_p1700 ~ Bn_A09_p1713_A9 | 28.68 - 29.66 | 0.98 |
|  | ***cqOC-A9-9*** | A09 | 105.79 - 106.77 | Bns_p2574_A9 ~ Bn_A09_p1830_A9 | 30.61 - 30.68 | 0.07 |
|  | ***cqOC-A10-1*** | A10 | 0 - 2.59 | Bn_A10_p1926 ~ Bn_A10_p2015 | 13.91 - 15.49 | 1.58 |
|  | ***cqOC-A10-2*** | A10 | 5.8-16.7 | Bn_A10_p2015 ~ niab144-A10 | 0.821 - 13.91 | 13.09 |
|  | ***cqOC-A10-3*** | A10 | 14.35 - 21.71 | Bn_A10_p1996_A10 ~ BnGMS625-A10/C9 |  |  |
|  | ***cqOC-C2-1*** | C02 | 82.1 - 86.18 | Bn_C_p2030_C2 ~ Bns_p2108_C2 | 37.24 - 39.02 | 1.79 |
|  | ***cqOC-C2-2*** | C02 | 89.17 - 93.04 | Bns_p2329_C2 ~ Bns_p3492_C2 | 39 - 41.76 | 2.76 |
|  | ***cqOC-C2-3*** | C02 | 97.6-103.1 | Bns_p2762 ~ Bns_p2933 | 40.35 - 43.54 | 3.19 |
|  | ***cqOC-C2-4*** | C02 | 109.45 - 112.72 | Bns_p2945_C2 ~ Bn_A02_p0334_C2 | 43.78 - 44.13 | 0.35 |
|  | ***cqOC-C3-1*** | C03 | 142.23 - 147.64 | Bn_A03_p0486_C3 ~ Bns_p2627_C3 | 0.079 - 1.592 | 1.51 |
|  | ***cqOC-C3-2*** | C03 | 163.56 - 168.65 | Bns_p3685 ~ Bns_p3238 | 2.63 - 3.386 | 0.76 |
|  | ***cqOC-C3-3*** | C03 | 171.6-173.9 | Bn_A03_p0598_C3 ~ Bns_p3459_C3 | 4.06 - 5.014 | 0.95 |
|  | ***cqOC-C3-4*** | C03 | 299.84 - 301.12 | Bns_p2064_C3 ~ Bns_p3674 | 52.35 - 52.62 | 0.28 |
|  | ***cqOC-C3-5*** | C03 | 308.17 - 309.86 | Bns_p2851 ~ Bns_p2708_C3 | 53.83 - 55.41 | 1.58 |
|  | ***cqOC-C3-6*** | C03 | 311.35 - 313.31 | Bns_p2190 ~ Bns_p2181_C3 | 55.83 - 56.37 | 0.54 |
|  | ***cqOC-C3-7*** | C03 | 314.17 - 322.64 | Bns_p2183_C3 ~ Bns_p3245_C3 | 56.37 - 58.52 | 2.15 |
|  | ***cqOC-C5-1*** | C05 | 58.2-85.8 | Bns_p2840_C5 ~ niab038-A9 | 40.06 - 0 |  |
|  | ***cqOC-C5-2*** | C05 | 123.4-136 | Na12G12 ~ Bns_p2550 | 40.06 - 0 |  |
|  | ***cqOC-C5-3*** | C05 | 181.38 - 184.99 | Bns_p3498_C5 ~ Bns_p2759_C5 | 9.283 - 11.18 | 1.90 |
|  | ***cqOC-C5-4*** | C05 | 190.1 - 192.06 | Bn_A09_p1842_C5 ~ Bns_p3562_C5 | 13.29 - 21.77 | 8.48 |
|  | ***cqOC-C5-5*** | C05 | 219.85 - 220.98 | B087P06-1-A5 ~ Bns_p2824_C5 | 39.46 - 40.1 | 0.64 |
|  | ***cqOC-C5-6*** | C05 | 229.22 - 231.26 | Bns_p2834_C5 ~ Bns_p2844 |  |  |
|  | ***cqOC-C5-7*** | C05 | 232.79 - 235.24 | Bns_p2843 ~ Bns_p2845 | 40.26 - 42.76 | 2.50 |
|  | ***cqOC-C5-8*** | C05 | 234.66 - 240.11 | Bns_p2848 ~ Bn_A02_p0315_C5 | 40.35 - 42.76 | 2.41 |
|  | ***cqOC-C5-9*** | C05 | 249.56 - 252.01 | Bns_p3552_C5 ~ Bns_p3066 | 41.02 - 41.34 | 0.32 |
|  | ***cqOC-C5-10*** | C05 | 257.20-259.40 | Bns_p3351_C5 ~ Bn_A05_p1054 | 41.75 - 42.6 | 0.86 |
|  | ***cqOC-C6-1*** | C06 | 15.25 - 32.72 | Bn_A07_p1388 ~ Bn_A01_p0157_C6 |  |  |
|  | ***cqOC-C6-2*** | C06 | 147.65 - 151.02 | Bns_p3085_C6 ~ Bns_p2208 | 18.72 - 21.8 | 3.08 |
|  | ***cqOC-C6-3*** | C06 | 152.6 - 162.4 | Bns_p2208 ~ Bns_p2144 | 21.8 - 34.28 | 12.47 |
|  | ***cqOC-C6-4*** | C06 | 155.2-169.8 | Bns_p2201 ~ Bns_p2694 |  |  |
|  | ***cqOC-C6-5*** | C06 | 180.18 - 183.45 | Bns_p2490_C6 ~ Bns_p3615_C6 |  |  |
|  | ***cqOC-C6-6*** | C06 | 187.58 - 188.96 | Bns_p3421_C6 ~ Bns_p3657_C6 |  |  |
|  | ***cqOC-C6-7*** | C006 | 195.2-197 | Bns_p2696_C6 ~ Bns_p3315 |  |  |
|  | ***cqOC-C6-8*** | C06 | 198.88 - 199.33 | Bns_p3363 ~ Bns_p2278_C6 |  |  |
|  | ***cqOC-C6-9*** | C06 | 200.4-202.3 | Bns_p3317 ~ Bns_p3366_C6 |  |  |
|  | ***cqOC-C6-10*** | C06 | 203.56 - 204.05 | Bns_p2141 ~ Bns_p2984 |  |  |
|  | ***cqOC-C6-11*** | C06 | 206.5-211.6 | Bns_p2985_C6 ~ Bns_p3008_C6 | 34.14 - 35.89 | 1.75 |
|  | ***cqOC-C8*** | C08 | 62.66 - 69.65 | Bns_p2503_C8 ~ Bns_p2572_C8 | 29.29 - 34.28 | 4.98 |
|  | ***cqOC-C9-1*** | C09 | 4.08 - 10.7 | FITO516b-N1 ~ CB10064 |  |  |
|  | ***cqOC-C9-2*** | C09 | 24.6-29.8 | Bns_p2914_C9 ~ Bns_p2925_C9 | 0.414 - 1.338 | 0.92 |
| **SPC** | ***cqPC-A2*** | A02 | 0 - 1.13 | B060E11-1b ~ B060E11-1a |  |  |
|  | ***cqPC-A3-1*** | A03 | 124.80-127.70 | Bn_A03_p0721_A3 ~ Bn_A03_p0674_A3 | 16.07 - 17.85 | 1.78 |
|  | ***cqPC-A3-2*** | A03 | 131.30-132.30 | Bn_A03_p0723_A3 ~ Bn_A03_p0451_A3 | 18.43 - 18.78 | 0.35 |
|  | ***cqPC-A3-3*** | A03 | 133.00-139.90 | Bn_A03_p0451_A3 ~ Bn_A03_p0627_A3 | 18.78 - 19.75 | 0.96 |
|  | ***cqPC-A3-4*** | A03 | 200.58 - 206.43 | CNU370a ~ BRAS010-N4/N9 |  |  |
|  | ***cqPC-A3-5*** | A03 | 214.60-216.90 | Bn_A03_p0622_A3 ~ Bns_p3715_A3 | 17.73 - 17.78 | 0.05 |
|  | ***cqPC-A4-1*** | A04 | 13.00-15.50 | Bn_A04_p0926 ~ Bn_A04_p0910 | 0.656 - 1.342 | 0.69 |
|  | ***cqPC-A4-2*** | A04 | 52.50-56.40 | Bn_A04_p0863_A4 ~ Bn_A04_p0870_A4 | 13.86 - 15.33 | 1.47 |
|  | ***cqPC-A7*** | A07 | 95.80-100.90 | Bn_A07_p1527_A7 ~ Bn_A07_p1528_A7 | 15.27 - 15.55 | 0.28 |
|  | ***cqPC-A9-1*** | A09 | -8.36 | Bns_p2294_A9 ~ Bn_A09_p1674 | 0.121 - 0.519 | 0.40 |
|  | ***cqPC-A9-2*** | A09 | 13.47 - 19.32 | Bn_A01_p0149_A9 ~ Bn_A09_p1874_A9 | 0.686 - 2.872 | 2.19 |
|  | ***cqPC-A9-3*** | A09 | 20.59 - 27.18 | Bns_p3309_A9 ~ Bn_A09_p1847_A9 | 2.872 - 4.756 | 1.88 |
|  | ***cqPC-A9-4*** | A09 | 31.20-33.40 | Bns_p3372 ~ Bn_A02_p0390 | 6.402 - 10.21 | 3.81 |
|  | ***cqPC-A9-5*** | A09 | 33.4 - 42.21 | Bn_A09_p1821 ~ Bn_A09_p1824_A9 | 5.484 - 10.43 | 4.95 |
|  | ***cqPC-C1-1*** | C01 | 93.05 - 94.53 | Bns_p2299_C1 ~ Bn_A01_p0136_C1 | 36.35 - 37.02 | 0.67 |
|  | ***cqPC-C1-2*** | C01 | 150.05 - 154.33 | Bns_p3713 ~ Bn_A02_p0273 |  |  |
|  | ***cqPC-C1-3*** | C01 | 158.0 0- 160.72 | Bn_A08_p1607 ~ Bn_A05_p0983 |  |  |
|  | ***cqPC-C1-4*** | C01 | 166.00-176.90 | Bns_p3666 ~ Bn_A10_p2024 |  |  |
|  | ***cqPC-C3-1*** | C03 | 287.50-288.40 | Bns_p3631_C3 ~ Bns_p3640_C3 | 49.12 - 49.24 | 0.12 |
|  | ***cqPC-C3-2*** | C03 | 297.38 - 300.15 | Bn_A08_p1627 ~ Bns_p2738_C3 | 50.23 - 52.62 | 2.39 |
|  | ***cqPC-C3-3*** | C03 | 302.84 - 304.59 | Bns_p2092_C3 ~ Bns_p3024_C3 | 52.63 - 55.71 | 3.08 |
|  | ***cqPC-C3-4*** | C03 | 310.1-319.9 | Bns_p2188_C3 ~ Bns_p2931_C3 | 55.71 - 56.64 | 0.94 |
|  | ***cqPC-C5-1*** | C05 | 121.3-136 | Na12G12 ~ Bns_p2547_C5 | 0.41 - 1.541 | 1.13 |
|  | ***cqPC-C5-2*** | C05 | 139.89 - 144.03 | Bns_p2547_C5 ~ Bns_p3413_C5 | 1.541 - 2.295 | 0.75 |
|  | ***cqPC-C5-3*** | C05 | 221.80-225.90 | CB10229-N15 ~ Bns_p2826 | 39.46 - 40.11 | 0.65 |
|  | ***cqPC-C5-4*** | C05 | 231.20-233.90 | Bns_p2827_C5 ~ Bns_p2373_C5 | 39.82 - 40.42 | 0.59 |
|  | ***cqPC-C5-5*** | C05 | 234.67 - 240.1 | Bns_p2848 ~ Bn_A02_p0315_C5 | 40.42 - 42.76 | 2.35 |
|  | ***cqPC-C5-6*** | C05 | 251.54 - 252.89 | Bns_p3335_C5 ~ Bns_p3334 | 40.72 - 40.83 | 0.11 |
|  | ***cqPC-C5-7*** | C05 | 260.26 - 261.37 | Bns_p3455_C5 ~ Bns_p3338_C5 | 41.54 - 42.6 | 1.06 |
|  | ***cqPC-C6-1*** | C06 | 50.31 - 58.58 | Na10C06-N16 ~ FITO035 |  |  |
|  | ***cqPC-C6-2*** | C06 | 145.01 - 153.8 | Bns_p3085_C6 ~ Bns_p2206 | 18.72 - 21.93 | 3.21 |
|  | ***cqPC-C6-3*** | C06 | 153.85 - 159.35 | Bns_p2205_C6 ~ Bns_p2616_C6 | 21.8 - 29.48 | 7.68 |
|  | ***cqPC-C7*** | C07 | 105.01 - 114.66 | CB10431-N17 ~ BRAS014b-N6/N17 |  |  |
|  | ***cqPC-C8*** | C08 | 11.3-16.7 | Na10F01 ~ Bns_p3623 | 9.089 - 14.85 | 5.77 |
|  | ***cqPC-C9-1*** | C09 | 0.0 - 6.7 | BnGMS371-A9/C9 ~ CNU406-A9 |  |  |
|  | ***cqPC-C9-2*** | C09 | 6.47 - 9.78 | SA97 ~ CB10064 |  |  |
|  | ***cqPC-C9-3*** | C09 | 69.35 - 71.72 | Bns_p3688_C9 ~ Bns_p2466 | 36.02 - 38.67 | 2.64 |
|  | ***cqPC-C9-4*** | C09 | 74.86 - 79.75 | Bns_p3584 ~ Bns_p2042 | 39.48 - 41.17 | 1.70 |

| **Table S8. The candidates related to acyl-lipid metabolism falling within the SOC-QTL regions.** | | | | | |
| --- | --- | --- | --- | --- | --- |
| **SOC-QTL** | **Gene alias** | **A. thaliana Locus** | **ARALIP pathway(s)** | **ARALIP Protein/gene abbrev** | **ARALIP Protein family name/isoform** |
| *cqOC-A1* | BnaA01g12060D | AT4G22240 | Miscellaneous: lipid related |  | Plastid Lipid-associated Protein |
| *cqOC-A1* | BnaA01g12140D | AT4G22330 | Sphingolipid Synthesis | CES1 | Ceramidase |
| *cqOC-A1* | BnaA01g12150D | AT4G22340 | Eukaryotic Phospholipid Synthesis |  | CDP-DAG Synthase |
| *cqOC-A1* | BnaA01g12260D | AT4G22520 | Cuticular Wax Synthesis | LTP type 6 | Lipid Transfer Protein type 6 |
| *cqOC-A1* | BnaA01g12290D | AT4G22550 | Plastidial Glycerolipid, Galactolipid and Sulfolipid Synthesis | LPP &beta; | Phosphatidate Phosphatase |
| *cqOC-A1* | BnaA01g12830D | AT1G73550 | Cuticular Wax Synthesis | LTP type 5 | Lipid Transfer Protein type 5 |
| *cqOC-A1* | BnaA01g13470D | AT4G23850 | Eukaryotic Phospholipid Synthesis | LACS4 | Long-Chain Acyl-CoA Synthetase |
| *cqOC-A1* | BnaA01g13630D | AT4G24160 | Lipase |  | Cardiolipin-Specific Deacylase |
| *cqOC-A1* | BnaA01g13710D | AT4G24230 | Eukaryotic Phospholipid Synthesis | ACBP3 | Acyl CoA Binding Protein; Acyl-CoA Binding Protein |
| *cqOC-A1* | BnaA01g14400D | AT4G25050 | Plastidial Fatty Acid Synthesis | ACP4 | Acyl Carrier Protein |
| *cqOC-A1* | BnaA01g14480D | AT4G25140 | TAG Synthesis |  | Oil-Body Oleosin |
| *cqOC-A1* | BnaA01g14970D | AT4G25750 | Cuticular Wax Synthesis | WBC4 / ABCG4 | ABC Transporter |
| *cqOC-A1* | BnaA01g15140D | AT4G25970 | Eukaryotic Phospholipid Synthesis | PSD3 | Phosphatidylserine Decarboxylase |
| *cqOC-A10-1* | BnaA10g19780D | AT5G13580 | Cuticular Wax Synthesis | WBC6; WBC6 / ABCG6 | ABC transporter; ABC Transporter |
| *cqOC-A10-1* | BnaA10g20920D | AT5G11650 | TAG Degradation |  | Monoacylglycerol Lipase (MAGL) |
| *cqOC-A10-1* | BnaA10g20970D | AT1G77590 | Plastidial Fatty Acid Synthesis | LACS9 | Long-Chain Acyl-CoA Synthetase (plastidial); Long-Chain Acyl-CoA Synthetase |
| *cqOC-A10-1* | BnaA10g21280D | AT5G11190 | Cuticular Wax Synthesis | SHN2 | SHN Transcription Factors |
| *cqOC-A10-1* | BnaA10g21780D | AT5G10480 | Fatty Acid Elongation and Cuticular Wax Synthesis | HACD; HCD/PAS2 | Hydroxyacyl-CoA Dehydratase |
| *cqOC-A10-1* | BnaA10g23000D | AT5G08460 | GDSL |  |  |
| *cqOC-A10-1* | BnaA10g23290D | AT5G08030 | Miscellaneous: lipid related |  | Glycerophosphoryl Diester Phosphodiesterase |
| *cqOC-A10-2* | BnaA10g02340D | AT1G04010 | Miscellaneous: lipid related |  | Phospholipid : Acyl acceptor Acyltransferase |
| *cqOC-A10-2* | BnaA10g02480D | AT1G04220 | Fatty Acid Elongation and Cuticular Wax Synthesis | KCS2 (DAISY); KCS2 | Ketoacyl-CoA Synthase |
| *cqOC-A10-2* | BnaA10g02780D | AT1G04640 | Mitochondrial Fatty Acid and Lipoic Acid Synthesis | LT | Lipoyltransferase |
| *cqOC-A10-2* | BnaA10g03520D | AT1G05790 | Lipase |  |  |
| *cqOC-A10-2* | BnaA10g03530D | AT1G05800 | Galactolipid degradation | DGL | Acylhydrolase (DAD1-like) |
| *cqOC-A10-2* | BnaA10g03830D | AT1G05630 | Lipid Signaling |  | Phosphoinositide 5-Phosphatase Type II |
| *cqOC-A10-2* | BnaA10g04030D | AT1G06080 | Miscellaneous: lipid related |  | Acyl-CoA Desaturase-like |
| *cqOC-A10-2* | BnaA10g04040D | AT1G06090 | Miscellaneous: lipid related |  | Acyl-CoA Desaturase-like |
| *cqOC-A10-2* | BnaA10g04100D | AT1G34260 | Lipid Signaling |  | Phosphatidylinositol-Phosphate Kinase type III |
| *cqOC-A10-2* | BnaA10g04260D | AT1G06520 | Cutin Synthesis | GPAT1 | Glycerol-3-Phosphate Acyltransferase (mitochondrial); Glycerol-3-Phosphate Acyltransferase |
| *cqOC-A10-2* | BnaA10g04450D | AT2G24560 | GDSL |  |  |
| *cqOC-A10-2* | BnaA10g04680D | AT1G07230 | Plastidial Glycerolipid, Galactolipid and Sulfolipid Synthesis | NPC1 (Non specific) | Phospholipase C (Non specific) |
| *cqOC-A10-2* | BnaA10g05240D | AT3G18570 | TAG Synthesis |  | Oil-Body Oleosin |
| *cqOC-A10-2* | BnaA10g05760D | AT5G51210 | TAG Synthesis |  | Oil-Body Oleosin |
| *cqOC-A10-2* | BnaA10g06020D | AT5G50690 | TAG Synthesis |  | Steroleosin |
| *cqOC-A10-2* | BnaA10g06730D | AT5G12420 | Cuticular Wax Synthesis |  | Bifunctional Wax Ester Synthase / Diacylglycerol Acyltransferase |
| *cqOC-A10-2* | BnaA10g07590D | AT5G52160 | Cuticular Wax Synthesis | LTP type 3 | Lipid Transfer Protein type 3 |
| *cqOC-A10-2* | BnaA10g07670D | AT5G51950 | Cutin Synthesis |  | Omega-Hydroxy Fatty Acyl Dehydrogenase; omega-Hydroxy Fatty Acyl Dehydrogenase |
| *cqOC-A10-2* | BnaA10g09280D | AT3G53510 | Cuticular Wax Synthesis | WBC2 / ABCG2 | ABC Transporter |
| *cqOC-A10-2* | BnaA10g09300D | AT1G08510 | Plastidial Fatty Acid Synthesis | FatB | Acyl-ACP Thioesterase B |
| *cqOC-A10-2* | BnaA10g09310D | AT5G55050 | GDSL |  |  |
| *cqOC-A10-2* | BnaA10g09320D | AT5G55050 | GDSL |  |  |
| *cqOC-A10-2* | BnaA10g09480D | AT5G55240 | TAG Synthesis |  | Caleosin |
| *cqOC-A10-2* | BnaA10g09530D | AT5G55340 | Cuticular Wax Synthesis |  | Wax Synthase |
| *cqOC-A10-2* | BnaA10g09590D | AT5G55460 | Cuticular Wax Synthesis | LTP type 3 | Lipid Transfer Protein type 3 |
| *cqOC-A10-2* | BnaA10g09610D | AT5G55380 | Cuticular Wax Synthesis |  | Wax Synthase |
| *cqOC-A10-2* | BnaA10g09620D | AT5G55350 | Cuticular Wax Synthesis |  | Wax Synthase |
| *cqOC-A10-2* | BnaA10g09630D | AT5G55360 | Cuticular Wax Synthesis |  | Wax Synthase |
| *cqOC-A10-2* | BnaA10g09640D | AT5G55410 | Cuticular Wax Synthesis | LTP type 3 | Lipid Transfer Protein type 3 |
| *cqOC-A10-2* | BnaA10g10730D | AT5G56480 | Cuticular Wax Synthesis | LTP type 3 | Lipid Transfer Protein type 3 |
| *cqOC-A10-2* | BnaA10g11040D | AT5G57020 | Miscellaneous: lipid related |  | Protein N-Myristoyltransferase |
| *cqOC-A10-2* | BnaA10g11230D | AT5G57190 | Eukaryotic Phospholipid Synthesis | PSD2 | Phosphatidylserine Decarboxylase |
| *cqOC-A10-2* | BnaA10g11680D | AT5G57800 | Cuticular Wax Synthesis | CER3/WAX2/YRE/FLP1 | CER3 Protein |
| *cqOC-A10-2* | BnaA10g13450D | AT5G60620 | TAG Synthesis | GPAT9? (mammalian homologue) | Glycerol-3-Phosphate Acyltransferase (mammalian homologue) |
| *cqOC-A10-2* | BnaA10g13850D | AT5G22500 | Cuticular Wax Synthesis | AlcFAR1 | Alcohol-forming Fatty Acyl-CoA Reductase |
| *cqOC-A10-2* | BnaA10g13920D | AT3G44540 | Cuticular Wax Synthesis | AlcFAR4 | Alcohol-forming Fatty Acyl-CoA Reductase (ER); Alcohol-forming Fatty Acyl-CoA Reductase |
| *cqOC-A10-2* | BnaA10g13960D | ATCG00500 | Plastidial Fatty Acid Synthesis | &beta;-CT (Subunit of Heteromeric ACCase) | Carboxyltransferase &beta; Subunit of Heteromeric ACCase |
| *cqOC-A10-2* | BnaA10g14700D | AT5G20840 | Lipid Signaling |  | Sac domain-containing Phosphoinositide Phosphatase |
| *cqOC-A10-2* | BnaA10g15720D | AT5G19410 | Cuticular Wax Synthesis | WBC24 / ABCG23 | ABC Transporter |
| *cqOC-A10-2* | BnaA10g15820D | AT5G19290 | TAG Degradation |  | Monoacylglycerol Lipase (MAGL); Lysophospholipase |
| *cqOC-A10-2* | BnaA10g15860D | AT5G19200 | Sphingolipid Synthesis |  | Ketosphinganine Reductase |
| *cqOC-A10-2* | BnaA10g16330D | AT5G18630 | TAG Degradation |  | Triacylglycerol Lipase (TAGL) |
| *cqOC-A10-2* | BnaA10g17000D | AT5G17780 | Lipid Signaling |  | Lysophospholipase |
| *cqOC-A10-2* | BnaA10g17650D | AT1G76680 | Lipid Signaling |  | Oxo-Phytodienoic Acid Reductase |
| *cqOC-A10-2* | BnaA10g17730D | AT2G03140 | Lipase |  |  |
| *cqOC-A10-2* | BnaA10g18080D | AT3G02630 | Plastidial Fatty Acid Synthesis | DES5 | Stearoyl-ACP Desaturase |
| *cqOC-A10-2* | BnaA10g18090D | AT5G16230 | Plastidial Fatty Acid Synthesis | DES3 | Stearoyl-ACP Desaturase |
| *cqOC-A10-2* | BnaA10g18180D | AT5G16120 | TAG Degradation |  | Monoacylglycerol Lipase (MAGL) |
| *cqOC-A10-2* | BnaA10g18250D | AT5G16080 | Lipase |  |  |
| *cqOC-A10-2* | BnaA10g18680D | AT5G15530 | Plastidial Fatty Acid Synthesis | BCCP2 (Subunit of Heteromeric ACCase) | Biotin Carboxyl Carrier Protein of Heteromeric ACCase |
| *cqOC-A10-2* | BnaA10g18720D | AT3G58210 | Phospholipase |  |  |
| *cqOC-A10-2* | BnaA10g19400D | AT5G14310 | Lipase |  |  |
| *cqOC-A10-2* | BnaA10g19480D | AT5G14180 | TAG Degradation |  | Triacylglycerol Lipase (TAGL) |
| *cqOC-A2-2* | BnaA02g12590D | AT1G66850 | Cuticular Wax Synthesis | LTP type 2 | Lipid Transfer Protein type 2 |
| *cqOC-A2-2* | BnaA02g13230D | AT1G67560 | Lipase |  | Lipoxygenase |
| *cqOC-A2-2* | BnaA02g13270D | AT1G77590 | Plastidial Fatty Acid Synthesis | LACS9 | Long-Chain Acyl-CoA Synthetase (plastidial); Long-Chain Acyl-CoA Synthetase |
| *cqOC-A2-2* | BnaA02g13310D | AT1G67730 | Fatty Acid Elongation and Cuticular Wax Synthesis | KCR1 | Ketoacyl-CoA Reductase |
| *cqOC-A2-2* | BnaA02g13510D | AT1G68000 | Eukaryotic Phospholipid Synthesis | PIS1 | Phosphatidylinositol Synthase |
| *cqOC-A2-3* | BnaA02g14460D | AT1G69640 | Sphingolipid Synthesis | SBH1 | Sphingobase C4-Hydroxylase |
| *cqOC-A2-3* | BnaA02g15090D | AT1G70670 | TAG Synthesis |  | Caleosin |
| *cqOC-A2-3* | BnaA02g15290D | AT1G71010 | Lipid Signaling |  | Phosphatidylinositol-Phosphate Kinase type III |
| *cqOC-A2-3* | BnaA02g15690D | AT1G71960 | Cuticular Wax Synthesis | WBC26 / ABCG25 | ABC Transporter |
| *cqOC-A2-3* | BnaA02g15770D | AT1G72110 | Cuticular Wax Synthesis |  | Bifunctional Wax Ester Synthase / Diacylglycerol Acyltransferase |
| *cqOC-A2-3* | BnaA02g15790D | AT1G72110 | Cuticular Wax Synthesis |  | Bifunctional Wax Ester Synthase / Diacylglycerol Acyltransferase |
| *cqOC-A2-3* | BnaA02g16020D | AT1G72520 | Lipase |  | Lipoxygenase |
| *cqOC-A2-3* | BnaA02g16070D | AT1G72700 | Miscellaneous: lipid related |  | Translocase |
| *cqOC-A2-3* | BnaA02g16200D | AT1G72970 | Cutin Synthesis | HTD | Omega-Hydroxy Fatty Acyl Dehydrogenase; omega-Hydroxy Fatty Acyl Dehydrogenase |
| *cqOC-A2-3* | BnaA02g16260D | AT1G73050 | Cutin Synthesis |  | Omega-Hydroxy Fatty Acyl Dehydrogenase; omega-Hydroxy Fatty Acyl Dehydrogenase |
| *cqOC-A2-3* | BnaA02g16270D | AT1G73050 | Cutin Synthesis |  | Omega-Hydroxy Fatty Acyl Dehydrogenase; omega-Hydroxy Fatty Acyl Dehydrogenase |
| *cqOC-A2-3* | BnaA02g16280D | AT1G73050 | Cutin Synthesis |  | Omega-Hydroxy Fatty Acyl Dehydrogenase; omega-Hydroxy Fatty Acyl Dehydrogenase |
| *cqOC-A2-3* | BnaA02g16470D | AT1G73550 | Cuticular Wax Synthesis | LTP type 5 | Lipid Transfer Protein type 5 |
| *cqOC-A2-3* | BnaA02g16520D | AT1G73680 | Lipid Signaling |  | &&alpha;;-Dioxygenase-Peroxidase (involved in fatty acid &&alpha;;-oxidation) |
| *cqOC-A2-3* | BnaA02g16570D | AT1G73780 | Cuticular Wax Synthesis | LTP type 2 | Lipid Transfer Protein type 2 |
| *cqOC-A2-3* | BnaA02g17050D | AT1G74960 | Plastidial Fatty Acid Synthesis | KASII; KASII (fab1) | Ketoacyl-ACP Synthase II |
| *cqOC-A2-3* | BnaA02g17090D | AT1G75020 | TAG Synthesis | LPAAT4 | 1-acylglycerol-3-phosphate acyltransferase; 1-Acylglycerol-3-Phosphate Acyltransferase |
| *cqOC-A2-3* | BnaA02g17170D | AT1G75020 | TAG Synthesis | LPAAT4 | 1-acylglycerol-3-phosphate acyltransferase; 1-Acylglycerol-3-Phosphate Acyltransferase |
| *cqOC-A2-3* | BnaA02g17240D | AT1G47620 | Cuticular Wax Synthesis | CYP96A8 | Midchain Alkane Hydroxylase / Cytochrome P45, 96A |
| *cqOC-A2-3* | BnaA02g17580D | AT1G75910 | Lipase |  |  |
| *cqOC-A2-3* | BnaA02g17590D | AT1G75930 | Lipase |  |  |
| *cqOC-A2-3* | BnaA02g17670D | AT1G76680 | Lipid Signaling |  | Oxo-Phytodienoic Acid Reductase |
| *cqOC-A2-3* | BnaA02g18240D | AT1G77660 | Lipid Signaling |  | Phosphatidylinositol-Phosphate Kinase type IB |
| *cqOC-A2-3* | BnaA02g18410D | AT1G77420 | TAG Degradation |  | Monoacylglycerol Lipase (MAGL) |
| *cqOC-A2-3* | BnaA02g18990D | AT3G57310 | Cuticular Wax Synthesis | LTP type 2 | Lipid Transfer Protein type 2 |
| *cqOC-A2-3* | BnaA02g19010D | AT1G78690 | Mitochondrial Phospholipid Synthesis |  | Cardiolipin Transacylase |
| *cqOC-A2-3* | BnaA02g20010D | AT2G34770 | Sphingolipid Synthesis | FAH1 | Fatty Acid 2-hydroxylase |
| *cqOC-A2-3* | BnaA02g20580D | AT5G24220 | Lipase |  | Lipid Acylhydrolase-like |
| *cqOC-A2-3* | BnaA02g21360D | AT4G12360 | Cuticular Wax Synthesis | LTP type 5 | Lipid Transfer Protein type 5 |
| *cqOC-A2-3* | BnaA02g21490D | AT4G31810 | Mitochondrial Fatty Acid and Lipoic Acid Synthesis | | Mitochondrial Enoyl-CoA Hydratase |
| *cqOC-A2-3* | BnaA02g21860D | AT4G11030 | Eukaryotic Phospholipid Synthesis | LACS5 | Long-Chain Acyl-CoA Synthetase |
| *cqOC-A2-3* | BnaA02g22340D | AT5G43760 | Fatty Acid Elongation and Cuticular Wax Synthesis | KCS2 | Ketoacyl-CoA Synthase |
| *cqOC-A2-3* | BnaA02g22950D | AT5G42170 | GDSL |  |  |
| *cqOC-A2-3* | BnaA02g23180D | AT5G42650 | Lipid Signaling |  | Allene Oxide Synthase |
| *cqOC-A2-3* | BnaA02g24170D | AT5G45950 | GDSL |  |  |
| *cqOC-A2-3* | BnaA02g24180D | AT5G45960 | GDSL |  |  |
| *cqOC-A2-3* | BnaA02g24270D | AT4G32170 | Cuticular Wax Synthesis | CYP96A2 | Midchain Alkane Hydroxylase / Cytochrome P45, 96A |
| *cqOC-A2-3* | BnaA02g24400D | AT5G46290 | Plastidial Fatty Acid Synthesis | KASI | Ketoacyl-ACP Synthase I |
| *cqOC-A2-3* | BnaA02g24550D | AT2G34690 | Sphingolipid Synthesis | ACD11 | Sphingosine Transfer Protein |
| *cqOC-A2-3* | BnaA02g24580D | AT2G34690 | Sphingolipid Synthesis | ACD11 | Sphingosine Transfer Protein |
| *cqOC-A2-3* | BnaA02g25590D | AT5G47630 | Mitochondrial Fatty Acid and Lipoic Acid Synthesis | | Acyl Carrier Protein |
| *cqOC-A2-3* | BnaA02g25680D | AT5G47730 | Miscellaneous: lipid related |  | Sec14-like Protein |
| *cqOC-A2-3* | BnaA02g25900D | AT3G43570 | GDSL |  |  |
| *cqOC-A3-1* | BnaA03g08520D | AT3G44540 | Cuticular Wax Synthesis | AlcFAR4 | Alcohol-forming Fatty Acyl-CoA Reductase (ER); Alcohol-forming Fatty Acyl-CoA Reductase |
| *cqOC-A3-1* | BnaA03g08950D | AT5G60340 | Mitochondrial Fatty Acid and Lipoic Acid Synthesis | HAD | Hydroxyacyl-ACP Dehydrase |
| *cqOC-A3-1* | BnaA03g09530D | AT5G59320 | Cuticular Wax Synthesis | LTP type 1 | Lipid Transfer Protein type 1 |
| *cqOC-A3-1* | BnaA03g09540D | AT5G59320 | Cuticular Wax Synthesis | LTP type 1 | Lipid Transfer Protein type 1 |
| *cqOC-A3-2* | BnaA03g11410D | AT5G55450 | Cuticular Wax Synthesis | LTP type 3 | Lipid Transfer Protein type 3 |
| *cqOC-A3-2* | BnaA03g11420D | AT5G55410 | Cuticular Wax Synthesis | LTP type 3 | Lipid Transfer Protein type 3 |
| *cqOC-A3-2* | BnaA03g11440D | AT5G55370 | Cuticular Wax Synthesis |  | Wax Synthase |
| *cqOC-A3-2* | BnaA03g11450D | AT5G55340 | Cuticular Wax Synthesis |  | Wax Synthase |
| *cqOC-A3-2* | BnaA03g11460D | AT5G55350 | Cuticular Wax Synthesis |  | Wax Synthase |
| *cqOC-A3-2* | BnaA03g11470D | AT5G55360 | Cuticular Wax Synthesis |  | Wax Synthase |
| *cqOC-A3-2* | BnaA03g11610D | AT5G55050 | GDSL |  |  |
| *cqOC-A3-2* | BnaA03g11620D | AT5G55050 | GDSL |  |  |
| *cqOC-A3-3* | BnaA03g29710D | AT3G06860 | beta-Oxidation | MFP2 | Multifunctional Protein |
| *cqOC-A3-3* | BnaA03g30020D | AT3G07450 | Cuticular Wax Synthesis | LTP type 3 | Lipid Transfer Protein type 3 |
| *cqOC-A3-3* | BnaA03g30260D | AT3G07960 | Lipid Signaling |  | Phosphatidylinositol-Phosphate Kinase type IB |
| *cqOC-A3-3* | BnaA03g30270D | AT3G07960 | Lipid Signaling |  | Phosphatidylinositol-Phosphate Kinase type IB |
| *cqOC-A3-3* | BnaA03g31050D | AT3G08770 | Cuticular Wax Synthesis | LTP type 1 | Lipid Transfer Protein type 1 |
| *cqOC-A3-3* | BnaA03g31060D | AT3G08770 | Cuticular Wax Synthesis | LTP type 1 | Lipid Transfer Protein type 1 |
| *cqOC-A3-3* | BnaA03g31600D | AT3G11170 | Plastidial Glycerolipid, Galactolipid and Sulfolipid Synthesis | FAD7 | Linoleate Desaturase |
| *cqOC-A3-3* | BnaA03g32450D | AT3G12800 | beta-Oxidation |  | Dienoyl-CoA Reductase |
| *cqOC-A3-4* | BnaA03g42860D | AT4G16820 | TAG degradation |  | Acylhydrolase (DAD1-like) |
| *cqOC-A3-4* | BnaA03g43040D | AT3G58210 | Phospholipase |  |  |
| *cqOC-A3-4* | BnaA03g43090D | AT4G17483 | Miscellaneous: lipid related |  | Thioesterase (PPT1-like) |
| *cqOC-A3-5* | BnaA03g44190D | AT4G19860 | Phospholipase |  | Phospholipid : Acyl acceptor Acyltransferase |
| *cqOC-A3-5* | BnaA03g44270D | AT4G19860 | Phospholipase |  | Phospholipid : Acyl acceptor Acyltransferase |
| *cqOC-A3-5* | BnaA03g44730D | AT4G21540 | Sphingolipid Synthesis |  | Long Chain Base Kinase |
| *cqOC-A3-5* | BnaA03g45270D | AT4G22300 | Lipid Signaling |  | Lysophospholipase |
| *cqOC-A3-5* | BnaA03g45300D | AT4G22330 | Sphingolipid Synthesis | CES1 | Ceramidase |
| *cqOC-A3-5* | BnaA03g45330D | AT4G22490 | Cuticular Wax Synthesis | LTP type 6 | Lipid Transfer Protein type 6 |
| *cqOC-A8-2* | BnaA08g10210D | AT4G22330 | Sphingolipid Synthesis | CES1 | Ceramidase |
| *cqOC-A8-2* | BnaA08g10330D | AT4G22520 | Cuticular Wax Synthesis | LTP type 6 | Lipid Transfer Protein type 6 |
| *cqOC-A8-2* | BnaA08g10850D | AT4G35110 | Phospholipase |  |  |
| *cqOC-A8-2* | BnaA08g10960D | AT4G34930 | Phospholipase |  | Glycosylphosphatidylinositol-specific Phospholipase C |
| *cqOC-A8-2* | BnaA08g11130D | AT4G34520 | Fatty Acid Elongation and Cuticular Wax Synthesis | KCS18/FAE1 | Ketoacyl-CoA Synthase |
| *cqOC-A8-2* | BnaA08g11140D | AT4G34510 | Fatty Acid Elongation and Cuticular Wax Synthesis | KCS17 | Ketoacyl-CoA Synthase |
| *cqOC-A8-2* | BnaA08g11440D | AT4G33790 | Cuticular Wax Synthesis | AlcFAR3 (CER4); AlcFAR3/CER4 | Alcohol-forming Fatty Acyl-CoA Reductase (ER); Alcohol-forming Fatty Acyl-CoA Reductase |
| *cqOC-A8-2* | BnaA08g11640D | AT4G34050 | Aromatic Suberin Synthesis |  | Caffeoyl-CoA O-Methyltransferase |
| *cqOC-A8-2* | BnaA08g11810D | AT4G33355 | Cuticular Wax Synthesis | LTP type 1 | Lipid Transfer Protein type 1 |
| *cqOC-A8-3* | BnaA08g12350D | AT4G31810 | Mitochondrial Fatty Acid and Lipoic Acid Synthesis | | Mitochondrial Enoyl-CoA Hydratase |
| *cqOC-A8-3* | BnaA08g12720D | AT4G31050 | Plastidial Fatty Acid Synthesis |  | Lipoyltransferase |
| *cqOC-A8-3* | BnaA08g12730D | AT4G31020 | Phospholipase |  |  |
| *cqOC-A8-3* | BnaA08g12780D | AT4G30950 | Plastidial Glycerolipid, Galactolipid and Sulfolipid Synthesis | FAD6 | Oleate Desaturase |
| *cqOC-A8-3* | BnaA08g12800D | AT4G30950 | Plastidial Glycerolipid, Galactolipid and Sulfolipid Synthesis | FAD6 | Oleate Desaturase |
| *cqOC-A8-3* | BnaA08g12850D | AT4G30880 | Cuticular Wax Synthesis | LTP type 3 | Lipid Transfer Protein type 3 |
| *cqOC-A8-3* | BnaA08g13200D | AT4G30140 | GDSL |  |  |
| *cqOC-A8-3* | BnaA08g13220D | AT4G30140 | GDSL |  |  |
| *cqOC-A8-3* | BnaA08g13410D | AT4G29460 | Phospholipase |  | Phospholipase A2 |
| *cqOC-A8-3* | BnaA08g13520D | AT4G29070 | Phospholipase |  | Phospholipase A2 |
| *cqOC-A8-3* | BnaA08g13870D | AT2G20900 | Lipid Signaling |  | Diacylglycerol Kinase |
| *cqOC-A8-3* | BnaA08g14190D | AT4G27030 | Plastidial Glycerolipid, Galactolipid and Sulfolipid Synthesis | FAD4 | Phosphatidylglycerol Desaturase |
| *cqOC-A8-3* | BnaA08g14200D | AT4G27030 | Plastidial Glycerolipid, Galactolipid and Sulfolipid Synthesis | FAD4 | Phosphatidylglycerol Desaturase |
| *cqOC-A8-6* | BnaA08g14540D | AT4G25140 | TAG Synthesis |  | Oil-Body Oleosin |
| *cqOC-A8-6* | BnaA08g14550D | AT4G25050 | Plastidial Fatty Acid Synthesis | ACP4 | Acyl Carrier Protein |
| *cqOC-A9-10* | BnaA09g45720D | AT1G14190 | Cutin Synthesis |  | Omega-Hydroxy Fatty Acyl Dehydrogenase; omega-Hydroxy Fatty Acyl Dehydrogenase |
| *cqOC-A9-10* | BnaA09g46200D | AT1G13580 | Sphingolipid Synthesis | LAG13 | Ceramide Synthase |
| *cqOC-A9-10* | BnaA09g46210D | AT1G13560 | Eukaryotic Phospholipid Synthesis |  | Diacylglycerol Cholinephosphotransferase |
| *cqOC-A9-10* | BnaA09g46440D | AT1G13210 | Miscellaneous: lipid related |  | Translocase |
| *cqOC-A9-10* | BnaA09g47830D | AT1G10900 | Lipid Signaling |  | Phosphatidylinositol-Phosphate Kinase type IB |
| *cqOC-A9-10* | BnaA09g48190D | AT1G59820 | Miscellaneous: lipid related |  | Translocase |
| *cqOC-A9-10* | BnaA09g48250D | AT5G35360 | Plastidial Fatty Acid Synthesis | BC (Subunit of Heteromeric ACCase) | Biotin Carboxylase of Heteromeric ACCase |
| *cqOC-A9-10* | BnaA09g48610D | AT1G08980 | Lipid Signaling |  | Fatty Acid Amide Hydrolase |
| *cqOC-A9-10* | BnaA09g50060D | AT1G06090 | Miscellaneous: lipid related |  | Acyl-CoA Desaturase-like |
| *cqOC-A9-10* | BnaA09g50070D | AT1G06090 | Miscellaneous: lipid related |  | Acyl-CoA Desaturase-like |
| *cqOC-A9-10* | BnaA09g50080D | AT1G06090 | Miscellaneous: lipid related |  | Acyl-CoA Desaturase-like |
| *cqOC-A9-10* | BnaA09g50250D | AT1G05630 | Lipid Signaling |  | Phosphoinositide 5-Phosphatase Type II |
| *cqOC-A9-10* | BnaA09g50430D | AT5G65158 | Lipase |  |  |
| *cqOC-A9-10* | BnaA09g50730D | AT1G04010 | Miscellaneous: lipid related |  | Phospholipid : Acyl acceptor Acyltransferase |
| *cqOC-A9-10* | BnaA09g51230D | AT1G02660 | Lipid acylhydrolase |  | Lipid Acylhydrolase-like |
| *cqOC-A9-11* | BnaA09g51510D | AT1G01090 | Plastidial Fatty Acid Synthesis | PDH (E1alpha: component of Pyruvate Dehydrogenase Complex ) | Pyruvate Dehydrogenase &alpha; subunit, E1a component of Pyruvate Dehydrogenase Complex |
| *cqOC-A9-11* | BnaA09g51530D | AT1G01120 | Fatty Acid Elongation and Cuticular Wax Synthesis | KCS1 | Ketoacyl-CoA Synthase |
| *cqOC-A9-2* | BnaA09g07080D | AT5G65110 | beta-Oxidation | ACX2 | Acyl-CoA Oxidase |
| *cqOC-A9-2* | BnaA09g07600D | AT5G67050 | TAG Degradation |  | Triacylglycerol Lipase (TAGL); Lipid Acylhydrolase-like |
| *cqOC-A9-2* | BnaA09g08140D | AT5G66020 | Lipid Signaling |  | Sac domain-containing Phosphoinositide Phosphatase |
| *cqOC-A9-2* | BnaA09g08200D | AT2G11810 | Plastidial Glycerolipid, Galactolipid and Sulfolipid Synthesis | MGD3 | Monogalactosyldiacylglycerol Synthase |
| *cqOC-A9-4* | BnaA09g12230D | AT2G46500 | Lipid Signaling |  | Phosphatidylinositol-4-Kinase &gamma; |
| *cqOC-A9-4* | BnaA09g12710D | AT1G62790 | Cuticular Wax Synthesis | LTP type 5 | Lipid Transfer Protein type 5 |
| *cqOC-A9-4* | BnaA09g12890D | AT1G62640 | Plastidial Fatty Acid Synthesis | KASIII | Ketoacyl-ACP Synthase III |
| *cqOC-A9-4* | BnaA09g12940D | AT1G63050 | TAG Synthesis |  | 1-acylglycerol-3-phosphocholine Acyltransferase |
| *cqOC-A9-4* | BnaA09g13270D | AT1G62430 | Eukaryotic Phospholipid Synthesis | CDS1 | CDP-DAG Synthase |
| *cqOC-A9-4* | BnaA09g13590D | AT1G61850 | Galactolipid degradation | AtPLA1 |  |
| *cqOC-A9-4* | BnaA09g13840D | AT3G15870 | Miscellaneous: lipid related |  | Acyl-CoA Desaturase-like |
| *cqOC-A9-4* | BnaA09g13990D | AT3G57310 | Cuticular Wax Synthesis | LTP type 2 | Lipid Transfer Protein type 2 |
| *cqOC-A9-4* | BnaA09g14000D | AT3G57310 | Cuticular Wax Synthesis | LTP type 2 | Lipid Transfer Protein type 2 |
| *cqOC-A9-4* | BnaA09g14090D | AT1G60890 | Lipid Signaling |  | Phosphatidylinositol-Phosphate Kinase type IB |
| *cqOC-A9-4* | BnaA09g14140D | AT1G60490 | Lipid Signaling |  | Phosphatidylinositol-3-Kinase |
| *cqOC-A9-4* | BnaA09g14610D | AT1G58725 | GDSL |  |  |
| *cqOC-A9-4* | BnaA09g15080D | AT1G57750 | Cuticular Wax Synthesis | CYP96A15/MAH1 | Midchain Alkane Hydroxylase / Cytochrome P45, 96A; Secondary Alcohol Dehydrogenase |
| *cqOC-A9-4* | BnaA09g15090D | AT1G57750 | Cuticular Wax Synthesis | CYP96A15/MAH1 | Midchain Alkane Hydroxylase / Cytochrome P45, 96A; Secondary Alcohol Dehydrogenase |
| *cqOC-A9-4* | BnaA09g15100D | AT1G57750 | Cuticular Wax Synthesis | CYP96A15/MAH1 | Midchain Alkane Hydroxylase / Cytochrome P45, 96A; Secondary Alcohol Dehydrogenase |
| *cqOC-A9-4* | BnaA09g15130D | AT1G57750 | Cuticular Wax Synthesis | CYP96A15/MAH1 | Midchain Alkane Hydroxylase / Cytochrome P45, 96A; Secondary Alcohol Dehydrogenase |
| *cqOC-A9-4* | BnaA09g15160D | AT1G57750 | Cuticular Wax Synthesis | CYP96A15/MAH1 | Midchain Alkane Hydroxylase / Cytochrome P45, 96A; Secondary Alcohol Dehydrogenase |
| *cqOC-A9-4* | BnaA09g15250D | AT1G56670 | GDSL |  |  |
| *cqOC-A9-4* | BnaA09g15550D | AT1G58725 | GDSL |  |  |
| *cqOC-A9-4* | BnaA09g15560D | AT1G58725 | GDSL |  |  |
| *cqOC-A9-4* | BnaA09g15670D | AT5G03610 | GDSL |  |  |
| *cqOC-A9-4* | BnaA09g15750D | AT5G42870 | TAG Synthesis |  | Phosphatidate Phosphatase (ER); Phosphatidate Phosphatase |
| *cqOC-A9-5* | BnaA09g34250D | AT3G54320 | Plastidial Fatty Acid Synthesis | WRI1 | AP2/ERWEBP Transcription Factors |
| *cqOC-A9-5* | BnaA09g34830D | AT3G55110 | Cuticular Wax Synthesis | WBC18 / ABCG18 | ABC Transporter |
| *cqOC-A9-5* | BnaA09g34870D | AT3G55180 | TAG Degradation |  | Monoacylglycerol Lipase (MAGL) |
| *cqOC-A9-5* | BnaA09g34910D | AT3G55290 | Plastidial Fatty Acid Synthesis |  | Ketoacyl-ACP Reductase |
| *cqOC-A9-5* | BnaA09g34930D | AT3G55360 | Fatty Acid Elongation and Cuticular Wax Synthesis | ECR; ECR/CER1 | Enoyl-CoA Reductase |
| *cqOC-A9-5* | BnaA09g35480D | AT3G55940 | Phospholipase |  | Phosphoinositide-specific Phospholipase C |
| *cqOC-A9-5* | BnaA09g35520D | AT3G56060 | Cutin Synthesis |  | Omega-Hydroxy Fatty Acyl Dehydrogenase; omega-Hydroxy Fatty Acyl Dehydrogenase |
| *cqOC-A9-5* | BnaA09g35530D | AT3G56060 | Cutin Synthesis |  | Omega-Hydroxy Fatty Acyl Dehydrogenase; omega-Hydroxy Fatty Acyl Dehydrogenase |
| *cqOC-A9-6* | BnaA09g40500D | AT2G26420 | Lipid Signaling |  | Phosphatidylinositol-Phosphate Kinase type IB |
| *cqOC-A9-7* | BnaA09g40640D | AT2G26250 | Fatty Acid Elongation and Cuticular Wax Synthesis | KCS1/FDH | Ketoacyl-CoA Synthase |
| *cqOC-A9-8* | BnaA09g41930D | AT2G23540 | GDSL |  |  |
| *cqOC-A9-8* | BnaA09g42120D | AT2G23180 | Cuticular Wax Synthesis | CYP96A1 | Midchain Alkane Hydroxylase / Cytochrome P45, 96A |
| *cqOC-A9-8* | BnaA09g42230D | AT2G22170 | Lipase |  |  |
| *cqOC-A9-9* | BnaA09g44570D | AT1G18460 | Lipase |  |  |
| *cqOC-A9-9* | BnaA09g44630D | AT1G18280 | Cuticular Wax Synthesis | LTP type 5 | Lipid Transfer Protein type 5 |
| *cqOC-A9-9* | BnaA09g44640D | AT1G18280 | Cuticular Wax Synthesis | LTP type 5 | Lipid Transfer Protein type 5 |
| *cqOC-A9-9* | BnaA09g44650D | AT1G18280 | Cuticular Wax Synthesis | LTP type 5 | Lipid Transfer Protein type 5 |
| *cqOC-C2-1* | BnaC02g35410D | AT1G13580 | Sphingolipid Synthesis | LAG13 | Ceramide Synthase |
| *cqOC-C2-1* | BnaC02g35520D | AT3G25585 | TAG Synthesis | DAG-CPT | Diacylglycerol Cholinephosphotransferase |
| *cqOC-C2-1* | BnaC02g35530D | AT3G25610 | Miscellaneous: lipid related |  | Translocase |
| *cqOC-C2-1* | BnaC02g35560D | AT3G25620 | Cuticular Wax Synthesis | WBC21 / ABCG21 | ABC Transporter |
| *cqOC-C2-2* | BnaC02g36350D | AT3G26790 | TAG Synthesis | FUS3 | Transcriptional factor with high similarity to the B3 region of the VP1/ABI3-like proteins |
| *cqOC-C2-2* | BnaC02g36400D | AT3G26840 | Lipase |  |  |
| *cqOC-C2-2* | BnaC02g36410D | AT3G26840 | Lipase |  |  |
| *cqOC-C2-2* | BnaC02g36440D | AT3G26840 | Lipase |  |  |
| *cqOC-C2-2* | BnaC02g37030D | AT3G27660 | TAG Synthesis |  | Oil-Body Oleosin |
| *cqOC-C2-2* | BnaC02g37160D | AT3G27950 | GDSL |  |  |
| *cqOC-C2-2* | BnaC02g37590D | AT3G28910 | Cuticular Wax Synthesis | MYB3 | Myb Transcription Factors |
| *cqOC-C2-2* | BnaC02g37710D | AT5G38170 | Cuticular Wax Synthesis | LTP type 2 | Lipid Transfer Protein type 2 |
| *cqOC-C2-2* | BnaC02g38640D | AT5G48490 | Cuticular Wax Synthesis | LTP type 3 | Lipid Transfer Protein type 3 |
| *cqOC-C2-3* | BnaC02g38800D | AT5G48880 | beta-Oxidation | PKT1 | Ketoacyl-CoA Thiolase |
| *cqOC-C2-3* | BnaC02g38930D | AT5G49070 | Fatty Acid Elongation and Cuticular Wax Synthesis | KCS21 | Ketoacyl-CoA Synthase |
| *cqOC-C2-3* | BnaC02g39080D | AT5G49460 | Cuticular Wax Synthesis | ACLB-2 | ATP Citrate Lyase B subunit |
| *cqOC-C2-3* | BnaC02g39270D | AT5G29560 | TAG Synthesis |  | Caleosin |
| *cqOC-C2-3* | BnaC02g39790D | AT5G27630 | Eukaryotic Phospholipid Synthesis | ACBP5 | Acyl CoA Binding Protein |
| *cqOC-C3-1* | BnaC03g00360D | AT5G01220 | Plastidial Glycerolipid, Galactolipid and Sulfolipid Synthesis | SQD2 | UDP-sulfoquinovose:DAG sulfoquinovosyltransferase |
| *cqOC-C3-1* | BnaC03g01340D | AT5G03610 | GDSL |  |  |
| *cqOC-C3-1* | BnaC03g01430D | AT5G03810 | GDSL |  |  |
| *cqOC-C3-1* | BnaC03g01520D | AT5G04040 | TAG Degradation | SDP1 | Triacylglycerol Lipase (TAGL); Acyl-Hydrolase (Patatin-like) |
| *cqOC-C3-1* | BnaC03g01530D | AT5G04040 | TAG Degradation | SDP1 | Triacylglycerol Lipase (TAGL); Acyl-Hydrolase (Patatin-like) |
| *cqOC-C3-1* | BnaC03g01830D | AT5G04930 | Lipid Trafficking | ALA1 | Aminophospholipid ATPase; Translocase |
| *cqOC-C3-1* | BnaC03g02910D | AT5G62200 | Lipase |  |  |
| *cqOC-C3-1* | BnaC03g03140D | AT5G07530 | TAG Synthesis |  | Pollen-surface Oleosin |
| *cqOC-C3-1* | BnaC03g03150D | AT5G07550 | TAG Synthesis |  | Pollen-surface Oleosin |
| *cqOC-C3-2* | BnaC03g05590D | AT5G12420 | Cuticular Wax Synthesis |  | Bifunctional Wax Ester Synthase / Diacylglycerol Acyltransferase |
| *cqOC-C3-2* | BnaC03g05600D | AT5G12420 | Cuticular Wax Synthesis |  | Bifunctional Wax Ester Synthase / Diacylglycerol Acyltransferase |
| *cqOC-C3-2* | BnaC03g06380D | AT5G14310 | Lipase |  |  |
| *cqOC-C3-2* | BnaC03g06650D | AT5G14930 | TAG Degradation | SAG11 | Triacylglycerol Lipase (TAGL); Lipid Acylhydrolase-like |
| *cqOC-C3-2* | BnaC03g06660D | AT5G14930 | TAG Degradation | SAG11 | Triacylglycerol Lipase (TAGL); Lipid Acylhydrolase-like |
| *cqOC-C3-2* | BnaC03g06680D | AT5G14930 | TAG Degradation | SAG11 | Triacylglycerol Lipase (TAGL); Lipid Acylhydrolase-like |
| *cqOC-C3-2* | BnaC03g07000D | AT5G15530 | Plastidial Fatty Acid Synthesis | BCCP2 (Subunit of Heteromeric ACCase) | Biotin Carboxyl Carrier Protein of Heteromeric ACCase |
| *cqOC-C3-3* | BnaC03g10300D | AT5G20840 | Lipid Signaling |  | Sac domain-containing Phosphoinositide Phosphatase |
| *cqOC-C3-5* | BnaC03g65000D | AT4G22550 | Plastidial Glycerolipid, Galactolipid and Sulfolipid Synthesis | LPP &beta; | Phosphatidate Phosphatase |
| *cqOC-C3-5* | BnaC03g65690D | AT4G34930 | Phospholipase |  | Glycosylphosphatidylinositol-specific Phospholipase C |
| *cqOC-C3-5* | BnaC03g65700D | AT4G34920 | Phospholipase |  | Glycosylphosphatidylinositol-specific Phospholipase C |
| *cqOC-C3-6* | BnaC03g65980D | AT4G34520 | Fatty Acid Elongation and Cuticular Wax Synthesis | KCS18/FAE1 | Ketoacyl-CoA Synthase |
| *cqOC-C3-6* | BnaC03g66150D | AT4G11850 | Phospholipase |  | Phospholipase D &gamma; |
| *cqOC-C3-6* | BnaC03g66380D | AT4G33790 | Cuticular Wax Synthesis | AlcFAR3 (CER4); AlcFAR3/CER4 | Alcohol-forming Fatty Acyl-CoA Reductase (ER); Alcohol-forming Fatty Acyl-CoA Reductase |
| *cqOC-C3-7* | BnaC03g67370D | AT4G31810 | Mitochondrial Fatty Acid and Lipoic Acid Synthesis | | Mitochondrial Enoyl-CoA Hydratase |
| *cqOC-C3-7* | BnaC03g67820D | AT4G30950 | Plastidial Glycerolipid, Galactolipid and Sulfolipid Synthesis | FAD6 | Oleate Desaturase |
| *cqOC-C3-7* | BnaC03g67860D | AT4G30880 | Cuticular Wax Synthesis | LTP type 3 | Lipid Transfer Protein type 3 |
| *cqOC-C3-7* | BnaC03g68070D | AT4G30140 | GDSL |  |  |
| *cqOC-C3-7* | BnaC03g68610D | AT1G08980 | Lipid Signaling |  | Fatty Acid Amide Hydrolase |
| *cqOC-C5-7* | BnaC05g43390D | AT3G09560 | TAG Synthesis |  | Phosphatidate Phosphatase (ER); Phosphatidate Phosphatase |
| *cqOC-C5-7* | BnaC05g43760D | AT3G08770 | Cuticular Wax Synthesis | LTP type 1 | Lipid Transfer Protein type 1 |
| *cqOC-C5-7* | BnaC05g44000D | AT3G08510 | Phospholipase |  | Phosphoinositide-specific Phospholipase C |
| *cqOC-C5-7* | BnaC05g44270D | AT3G07690 | TAG Synthesis |  | NAD-dependent Glycerol-3-Phosphate Dehydrogenase |
| *cqOC-C5-7* | BnaC05g44510D | AT3G07450 | Cuticular Wax Synthesis | LTP type 3 | Lipid Transfer Protein type 3 |
| *cqOC-C5-7* | BnaC05g44540D | AT3G07400 | Lipid acylhydrolase |  | Lipid Acylhydrolase-like |
| *cqOC-C5-7* | BnaC05g45070D | AT3G06860 | beta-Oxidation | MFP2 | Multifunctional Protein |
| *cqOC-C5-7* | BnaC05g45760D | AT3G06060 | Sphingolipid Synthesis |  | Ketosphinganine Reductase |
| *cqOC-C5-7* | BnaC05g45860D | AT3G05970 | beta-Oxidation | LACS6 | Long-Chain Acyl-CoA Synthetase (peroxisomal) |
| *cqOC-C5-7* | BnaC05g46370D | AT3G05420 | Eukaryotic Phospholipid Synthesis | ACBP4 | Acyl CoA Binding Protein; Acyl-CoA Binding Protein |
| *cqOC-C5-7* | BnaC05g46590D | AT3G01420 | Lipid Signaling |  | &&alpha;;-Dioxygenase-Peroxidase (involved in fatty acid &&alpha;;-oxidation) |
| *cqOC-C5-7* | BnaC05g46600D | AT3G05180 | GDSL |  |  |
| *cqOC-C5-7* | BnaC05g47350D | AT3G04290 | GDSL |  |  |
| *cqOC-C5-7* | BnaC05g47870D | AT3G03310 | Miscellaneous: lipid related |  | Phospholipid : Acyl acceptor Acyltransferase |
| *cqOC-C5-3* | BnaC05g15620D | AT1G20130 | GDSL |  |  |
| *cqOC-C5-3* | BnaC05g15630D | AT1G20132 | GDSL |  |  |
| *cqOC-C5-3* | BnaC05g17280D | AT1G21920 | Lipid Signaling |  | Phosphatidylinositol-Phosphate Kinase type IB |
| *cqOC-C5-4* | BnaC05g20420D | AT1G26270 | Lipid Signaling |  | Phosphatidylinositol-4-Kinase &gamma; |
| *cqOC-C5-4* | BnaC05g21070D | AT1G24470 | Fatty Acid Elongation and Cuticular Wax Synthesis | KCR2 | Ketoacyl-CoA Reductase |
| *cqOC-C5-4* | BnaC05g21140D | AT1G24360 | Plastidial Fatty Acid Synthesis |  | Ketoacyl-ACP Reductase |
| *cqOC-C5-4* | BnaC05g21950D | AT1G28580 | GDSL |  |  |
| *cqOC-C5-4* | BnaC05g21980D | AT1G28580 | GDSL |  |  |
| *cqOC-C5-4* | BnaC05g23260D | AT1G30370 | Lipase |  | Acylhydrolase (DAD1-like) |
| *cqOC-C5-4* | BnaC05g23380D | AT1G06800 | TAG degradation |  | Acylhydrolase (DAD1-like) |
| *cqOC-C5-4* | BnaC05g24040D | AT1G15110 | Eukaryotic Phospholipid Synthesis |  | Base-Exchange-type Phosphatidylserine Synthase |
| *cqOC-C5-4* | BnaC05g24310D | AT1G32190 | Phospholipase |  |  |
| *cqOC-C5-4* | BnaC05g24510D | AT5G16390 | Plastidial Fatty Acid Synthesis | BCCP1 (Subunit of Heteromeric ACCase) | Biotin Carboxyl Carrier Protein of Heteromeric ACCase |
| *cqOC-C5-4* | BnaC05g25790D | AT1G48750 | Cuticular Wax Synthesis | LTP type 2 | Lipid Transfer Protein type 2 |
| *cqOC-C5-5* | BnaC05g41970D | AT3G11210 | GDSL |  |  |
| *cqOC-C5-5* | BnaC05g42010D | AT3G11170 | Plastidial Glycerolipid, Galactolipid and Sulfolipid Synthesis | FAD7 | Linoleate Desaturase |
| *cqOC-C5-5* | BnaC05g42450D | AT3G10570 | Cutin Synthesis | CYP77A6 | Fatty Acyl in-chain Hydroxylase (CYP77A) |
| *cqOC-C5-5* | BnaC05g42630D | AT3G14270 | Lipid Signaling |  | Phosphatidylinositol-Phosphate Kinase type III |
| *cqOC-C5-5* | BnaC05g42730D | AT3G10370 | Mitochondrial Phospholipid Synthesis | SDP6 | FAD-dependent Glycerol-3-Phosphate Dehydrogenase |
| *cqOC-C5-5* | BnaC05g43060D | AT3G09930 | GDSL |  |  |
| *cqOC-C6-2* | BnaC06g15900D | AT2G04350 | Plastidial Fatty Acid Synthesis | LACS8 | Long-Chain Acyl-CoA Synthetase (plastidial); Long-Chain Acyl-CoA Synthetase |
| *cqOC-C6-2* | BnaC06g16040D | AT3G56700 | Cuticular Wax Synthesis | AlcFAR6 | Alcohol-forming Fatty Acyl-CoA Reductase |
| *cqOC-C6-2* | BnaC06g16060D | AT3G56700 | Cuticular Wax Synthesis | AlcFAR6 | Alcohol-forming Fatty Acyl-CoA Reductase |
| *cqOC-C6-2* | BnaC06g16390D | AT3G58270 | Phospholipase |  |  |
| *cqOC-C6-2* | BnaC06g16550D | AT3G58320 | Phospholipase |  |  |
| *cqOC-C6-2* | BnaC06g16600D | AT3G58490 | Plastidial Glycerolipid, Galactolipid and Sulfolipid Synthesis | LPP &delta; | Long Chain Base 1-Phosphate Phosphatase; Phosphatidate Phosphatase |
| *cqOC-C6-2* | BnaC06g16630D | AT3G58550 | Cuticular Wax Synthesis | LTP type 7 | Lipid Transfer Protein type 7 |
| *cqOC-C6-2* | BnaC06g17540D | AT3G59770 | Lipid Signaling |  | Sac domain-containing Phosphoinositide Phosphatase |
| *cqOC-C6-2* | BnaC06g17800D | AT3G60500 | Cuticular Wax Synthesis | CER7 | CER7 Protein involved in wax synthesis |
| *cqOC-C6-2* | BnaC06g17810D | AT3G60510 | Mitochondrial Fatty Acid and Lipoic Acid Synthesis | | Mitochondrial Enoyl-CoA Hydratase |
| *cqOC-C6-2* | BnaC06g18430D | AT3G62280 | GDSL |  |  |
| *cqOC-C6-2* | BnaC06g18870D | AT3G45140 | Lipid Signaling |  | Lipoxygenase |
| *cqOC-C6-3/4/5/6/7/8/9/10* | BnaC06g20770D | AT1G77660 | Lipid Signaling |  | Phosphatidylinositol-Phosphate Kinase type IB |
| *cqOC-C6-3/4/5/6/7/8/9/10* | BnaC06g20910D | AT1G77590 | Plastidial Fatty Acid Synthesis | LACS9 | Long-Chain Acyl-CoA Synthetase (plastidial); Long-Chain Acyl-CoA Synthetase |
| *cqOC-C6-3/4/5/6/7/8/9/10* | BnaC06g21010D | AT1G77420 | TAG Degradation |  | Monoacylglycerol Lipase (MAGL) |
| *cqOC-C6-3/4/5/6/7/8/9/10* | BnaC06g22050D | AT1G75900 | GDSL |  |  |
| *cqOC-C6-3/4/5/6/7/8/9/10* | BnaC06g22060D | AT1G75880 | GDSL |  |  |
| *cqOC-C6-3/4/5/6/7/8/9/10* | BnaC06g22070D | AT1G75880 | GDSL |  |  |
| *cqOC-C6-3/4/5/6/7/8/9/10* | BnaC06g22630D | AT1G75020 | TAG Synthesis | LPAAT4 | 1-acylglycerol-3-phosphate acyltransferase; 1-Acylglycerol-3-Phosphate Acyltransferase |
| *cqOC-C6-3/4/5/6/7/8/9/10* | BnaC06g22680D | AT1G74960 | Plastidial Fatty Acid Synthesis | KASII; KASII (fab1) | Ketoacyl-ACP Synthase II |
| *cqOC-C6-3/4/5/6/7/8/9/10* | BnaC06g22960D | AT1G74460 | GDSL |  |  |
| *cqOC-C6-3/4/5/6/7/8/9/10* | BnaC06g23190D | AT1G74210 | Miscellaneous: lipid related |  | Glycerophosphoryl Diester Phosphodiesterase |
| *cqOC-C6-3/4/5/6/7/8/9/10* | BnaC06g23200D | AT1G74210 | Miscellaneous: lipid related |  | Glycerophosphoryl Diester Phosphodiesterase |
| *cqOC-C6-3/4/5/6/7/8/9/10* | BnaC06g23230D | AT1G74210 | Miscellaneous: lipid related |  | Glycerophosphoryl Diester Phosphodiesterase |
| *cqOC-C6-3/4/5/6/7/8/9/10* | BnaC06g23310D | AT1G73920 | Lipase |  |  |
| *cqOC-C6-3/4/5/6/7/8/9/10* | BnaC06g23490D | AT1G73680 | Lipid Signaling |  | &&alpha;;-Dioxygenase-Peroxidase (involved in fatty acid &&alpha;;-oxidation) |
| *cqOC-C6-3/4/5/6/7/8/9/10* | BnaC06g23520D | AT1G73600 | Eukaryotic Phospholipid Synthesis |  | Phosphoethanolamine N-Methyltransferase |
| *cqOC-C6-3/4/5/6/7/8/9/10* | BnaC06g23540D | AT1G73560 | Cuticular Wax Synthesis | LTP type 5 | Lipid Transfer Protein type 5 |
| *cqOC-C6-3/4/5/6/7/8/9/10* | BnaC06g23560D | AT1G73480 | TAG Degradation |  | Monoacylglycerol Lipase (MAGL) |
| *cqOC-C6-3/4/5/6/7/8/9/10* | BnaC06g23880D | AT1G72970 | Cutin Synthesis | HTD | Omega-Hydroxy Fatty Acyl Dehydrogenase; omega-Hydroxy Fatty Acyl Dehydrogenase |
| *cqOC-C6-3/4/5/6/7/8/9/10* | BnaC06g24570D | AT1G70670 | TAG Synthesis |  | Caleosin |
| *cqOC-C6-3/4/5/6/7/8/9/10* | BnaC06g25680D | AT1G68710 | Miscellaneous: lipid related |  | Translocase |
| *cqOC-C6-3/4/5/6/7/8/9/10* | BnaC06g25830D | AT1G68530 | Fatty Acid Elongation and Cuticular Wax Synthesis | KCS6 | Ketoacyl-CoA Synthase |
| *cqOC-C6-3/4/5/6/7/8/9/10* | BnaC06g25920D | AT2G30550 | TAG degradation | FAD4 | Acylhydrolase (DAD1-like) |
| *cqOC-C6-3/4/5/6/7/8/9/10* | BnaC06g25940D | AT5G46290 | Plastidial Fatty Acid Synthesis | KASI | Ketoacyl-ACP Synthase I |
| *cqOC-C6-3/4/5/6/7/8/9/10* | *BnaC06g26180D* | AT1G68000 | Eukaryotic Phospholipid Synthesis | PIS1 | Phosphatidylinositol Synthase |
| *cqOC-C6-3/4/5/6/7/8/9/10* | BnaC06g26190D | AT3G45140 | Lipid Signaling |  | Lipoxygenase |
| *cqOC-C6-3/4/5/6/7/8/9/10* | BnaC06g26670D | AT1G67560 | Lipase |  | Lipoxygenase |
| *cqOC-C6-3/4/5/6/7/8/9/10* | BnaC06g26900D | AT1G36180 | Plastidial Fatty Acid Synthesis | ACC2 (Homomeric) | Acetyl-CoA Carboxylase (Plastidial, Homomeric) |
| *cqOC-C6-3/4/5/6/7/8/9/10* | BnaC06g26980D | AT1G65060 | Aromatic Suberin Synthesis | At-4CL3 | 4-Coumarate-CoA Ligase |
| *cqOC-C6-3/4/5/6/7/8/9/10* | BnaC06g26990D | AT1G65290 | Mitochondrial Fatty Acid and Lipoic Acid Synthesis | | Acyl Carrier Protein |
| *cqOC-C6-3/4/5/6/7/8/9/10* | BnaC06g27030D | AT1G65410 | Lipid Trafficking | TGD3 | ATPase |
| *cqOC-C6-3/4/5/6/7/8/9/10* | BnaC06g27120D | AT1G65580 | Lipid Signaling |  | Phosphoinositide 5-Phosphatase Type II |
| *cqOC-C6-3/4/5/6/7/8/9/10* | BnaC06g27820D | AT1G66850 | Cuticular Wax Synthesis | LTP type 2 | Lipid Transfer Protein type 2 |
| *cqOC-C6-3/4/5/6/7/8/9/10* | BnaC06g28830D | AT1G67730 | Fatty Acid Elongation and Cuticular Wax Synthesis | KCR1 | Ketoacyl-CoA Reductase |
| *cqOC-C6-3/4/5/6/7/8/9/10* | BnaC06g30250D | AT4G39670 | Sphingolipid Synthesis |  | Sphingosine Transfer Protein |
| *cqOC-C6-3/4/5/6/7/8/9/10* | BnaC06g30340D | AT1G68710 | Miscellaneous: lipid related |  | Translocase |
| *cqOC-C6-3/4/5/6/7/8/9/10* | BnaC06g31770D | AT1G70670 | TAG Synthesis |  | Caleosin |
| *cqOC-C6-3/4/5/6/7/8/9/10* | BnaC06g32150D | AT1G71010 | Lipid Signaling |  | Phosphatidylinositol-Phosphate Kinase type III |
| *cqOC-C6-3/4/5/6/7/8/9/10* | BnaC06g32670D | AT1G71697 | TAG Synthesis | CK1 | Choline Kinase |
| *cqOC-C6-3/4/5/6/7/8/9/10* | BnaC06g32770D | AT1G14290 | Sphingolipid Synthesis | SBH2 | Sphingobase C4-Hydroxylase |
| *cqOC-C6-3/4/5/6/7/8/9/10* | BnaC06g32780D | AT1G69640 | Sphingolipid Synthesis | SBH1 | Sphingobase C4-Hydroxylase |
| *cqOC-C6-3/4/5/6/7/8/9/10* | BnaC06g32970D | AT1G71960 | Cuticular Wax Synthesis | WBC26 / ABCG25 | ABC Transporter |
| *cqOC-C6-3/4/5/6/7/8/9/10* | BnaC06g33850D | AT1G72700 | Miscellaneous: lipid related |  | Translocase |
| *cqOC-C6-3/4/5/6/7/8/9/10* | BnaC06g34390D | AT1G73480 | TAG Degradation |  | Monoacylglycerol Lipase (MAGL) |
| *cqOC-C6-3/4/5/6/7/8/9/10* | BnaC06g34400D | AT1G73600 | Eukaryotic Phospholipid Synthesis |  | Phosphoethanolamine N-Methyltransferase |
| *cqOC-C6-3/4/5/6/7/8/9/10* | BnaC06g34420D | AT1G73610 | GDSL |  |  |
| *cqOC-C6-3/4/5/6/7/8/9/10* | BnaC06g34480D | AT1G73680 | Lipid Signaling |  | &&alpha;;-Dioxygenase-Peroxidase (involved in fatty acid &&alpha;;-oxidation) |
| *cqOC-C6-3/4/5/6/7/8/9/10* | BnaC06g34730D | AT1G73890 | Cuticular Wax Synthesis | LTP type 5 | Lipid Transfer Protein type 5 |
| *cqOC-C6-3/4/5/6/7/8/9/10* | BnaC06g34740D | AT1G73920 | Lipase |  |  |
| *cqOC-C6-11* | BnaC06g35160D | AT1G74210 | Miscellaneous: lipid related |  | Glycerophosphoryl Diester Phosphodiesterase |
| *cqOC-C6-11* | BnaC06g35230D | AT1G74320 | TAG Synthesis |  | Choline Kinase |
| *cqOC-C6-11* | BnaC06g35760D | AT1G74960 | Plastidial Fatty Acid Synthesis | KASII; KASII (fab1) | Ketoacyl-ACP Synthase II |
| *cqOC-C6-11* | BnaC06g36580D | AT1G75890 | GDSL |  |  |
| *cqOC-C6-11* | BnaC06g36590D | AT1G75900 | GDSL |  |  |
| *cqOC-C6-11* | BnaC06g36850D | AT1G76150 | beta-Oxidation | ECH2 | Peroxisomal Enoyl-CoA Hydratase 2 |
| *cqOC-C8* | BnaC08g18910D | AT1G19640 | Lipid Signaling |  | Jasmonic Acid Carboxyl Methyltransferase |
| *cqOC-C8* | BnaC08g19000D | AT1G19640 | Lipid Signaling |  | Jasmonic Acid Carboxyl Methyltransferase |
| *cqOC-C8* | BnaC08g19190D | AT1G19800 | Lipid Trafficking | TGD1 | Permease-like Protein of Inner Chloroplast Envelope |
| *cqOC-C8* | BnaC08g19540D | AT4G30950 | Plastidial Glycerolipid, Galactolipid and Sulfolipid Synthesis | FAD6 | Oleate Desaturase |
| *cqOC-C8* | BnaC08g20040D | AT1G21920 | Lipid Signaling |  | Phosphatidylinositol-Phosphate Kinase type IB |
| *cqOC-C8* | BnaC08g20060D | AT1G21970 | TAG Synthesis | LEC1 | Transcriptional activator of genes required for both embryo maturation and cellular differentiation |
| *cqOC-C8* | BnaC08g20070D | AT1G21980 | Lipid Signaling |  | Phosphatidylinositol-Phosphate Kinase type IB |
| *cqOC-C8* | BnaC08g20350D | AT3G48610 | Plastidial Glycerolipid, Galactolipid and Sulfolipid Synthesis | NPC6 (Non specific) | Phospholipase C (Non specific) |
| *cqOC-C8* | BnaC08g20710D | AT3G49050 | Lipid acylhydrolase |  | Lipid Acylhydrolase-like |
| *cqOC-C8* | BnaC08g20870D | AT3G49210 | Cuticular Wax Synthesis |  | Bifunctional Wax Ester Synthase / Diacylglycerol Acyltransferase |
| *cqOC-C8* | BnaC08g21900D | AT3G50400 | GDSL |  |  |
| *cqOC-C8* | BnaC08g22120D | AT3G50790 | Lipase |  |  |
| *cqOC-C8* | BnaC08g22310D | AT3G50920 | Plastidial Glycerolipid, Galactolipid and Sulfolipid Synthesis | LPP &epsilon;1 | Phosphatidate Phosphatase |
| *cqOC-C8* | BnaC08g22910D | AT3G51590 | Cuticular Wax Synthesis | LTP type 1 | Lipid Transfer Protein type 1 |
| *cqOC-C8* | BnaC08g22920D | AT3G51600 | Cuticular Wax Synthesis | LTP type 1 | Lipid Transfer Protein type 1 |
| *cqOC-C8* | BnaC08g23150D | AT3G51840 | beta-Oxidation | ACX4 | Acyl-CoA Oxidase |
| *cqOC-C8* | BnaC08g23460D | AT3G52130 | Cuticular Wax Synthesis | LTP type 3 | Lipid Transfer Protein type 3 |
| *cqOC-C8* | BnaC08g23660D | AT3G52430 | Lipase |  |  |
| *cqOC-C8* | BnaC08g24540D | AT3G53510 | Cuticular Wax Synthesis | WBC2 / ABCG2 | ABC Transporter |
| *cqOC-C8* | BnaC08g24920D | AT1G77590 | Plastidial Fatty Acid Synthesis | LACS9 | Long-Chain Acyl-CoA Synthetase (plastidial); Long-Chain Acyl-CoA Synthetase |
| *cqOC-C8* | BnaC08g25150D | AT3G54320 | Plastidial Fatty Acid Synthesis | WRI1 | AP2/ERWEBP Transcription Factors |
| *cqOC-C8* | BnaC08g25800D | AT3G54950 | Phospholipase |  | Acyl-Hydrolase (Patatin-like) |
| *cqOC-C8* | BnaC08g25880D | AT3G55030 | Eukaryotic Phospholipid Synthesis | PGPS2 | Phosphatidylglycerol-Phosphate Synthase |
| *cqOC-C8* | BnaC08g25990D | AT3G55110 | Cuticular Wax Synthesis | WBC18 / ABCG18 | ABC Transporter |
| *cqOC-C8* | BnaC08g26060D | AT3G55180 | TAG Degradation |  | Monoacylglycerol Lipase (MAGL) |
| *cqOC-C8* | BnaC08g26120D | AT3G55290 | Plastidial Fatty Acid Synthesis |  | Ketoacyl-ACP Reductase |
| *cqOC-C8* | BnaC08g26140D | AT3G55360 | Fatty Acid Elongation and Cuticular Wax Synthesis | ECR; ECR/CER1 | Enoyl-CoA Reductase |
| *cqOC-C8* | BnaC08g26860D | AT3G55940 | Phospholipase |  | Phosphoinositide-specific Phospholipase C |
| *cqOC-C8* | BnaC08g26960D | AT3G56040 | Plastidial Glycerolipid, Galactolipid and Sulfolipid Synthesis | UGPase, UGP3; UGP3 | UDP-Glucose Pyrophosphorylase; UDP-glucose pyrophosphorylase |
| *cqOC-C8* | BnaC08g26980D | AT3G56060 | Cutin Synthesis |  | Omega-Hydroxy Fatty Acyl Dehydrogenase; omega-Hydroxy Fatty Acyl Dehydrogenase |
| *cqOC-C8* | BnaC08g27500D | AT3G56600 | Lipid Signaling |  | Phosphatidylinositol-4-Kinase &gamma; |
| *cqOC-C8* | BnaC08g27570D | AT3G56700 | Cuticular Wax Synthesis | AlcFAR6 | Alcohol-forming Fatty Acyl-CoA Reductase |
| *cqOC-C8* | BnaC08g27580D | AT3G56700 | Cuticular Wax Synthesis | AlcFAR6 | Alcohol-forming Fatty Acyl-CoA Reductase |
| *cqOC-C8* | BnaC08g27860D | AT3G56960 | Lipid Signaling |  | Phosphatidylinositol-Phosphate Kinase type IB |
| *cqOC-C8* | BnaC08g27870D | AT3G56960 | Lipid Signaling |  | Phosphatidylinositol-Phosphate Kinase type IB |
| *cqOC-C8* | BnaC08g28660D | AT3G57650 | TAG Synthesis | LPAAT2 | 1-acylglycerol-3-phosphate acyltransferase; 1-Acylglycerol-3-Phosphate Acyltransferase |
| *cqOC-C8* | BnaC08g29950D | AT3G59770 | Lipid Signaling |  | Sac domain-containing Phosphoinositide Phosphatase |
| *cqOC-C8* | BnaC08g30710D | AT3G60500 | Cuticular Wax Synthesis | CER7 | CER7 Protein involved in wax synthesis |
| *cqOC-C8* | BnaC08g30790D | AT3G60620 | Plastidial Glycerolipid, Galactolipid and Sulfolipid Synthesis | | CDP-DAG Synthase (plastidial) |
| *cqOC-C8* | BnaC08g31440D | AT3G61580 | Sphingolipid Synthesis | SLD1 | Sphingobase-D8 Desaturase |
| *cqOC-C8* | BnaC08g32310D | AT3G62590 | Lipid acylhydrolase |  | Lipid Acylhydrolase-like |
| *cqOC-C8* | BnaC08g32530D | AT3G62860 | TAG Degradation |  | Monoacylglycerol Lipase (MAGL) |
| *cqOC-C8* | BnaC08g32750D | AT3G63200 | Phospholipase |  | Acyl-Hydrolase (Patatin-like) |
| *cqOC-C8* | BnaC08g32850D | AT3G51970 | Cuticular Wax Synthesis |  | Wax Synthase |
| *cqOC-C8* | BnaC08g32950D | AT2G26420 | Lipid Signaling |  | Phosphatidylinositol-Phosphate Kinase type IB |
| *cqOC-C8* | BnaC08g33100D | AT2G26250 | Fatty Acid Elongation and Cuticular Wax Synthesis | KCS1/FDH | Ketoacyl-CoA Synthase |
| *cqOC-C8* | BnaC08g33530D | AT2G25170 | TAG Synthesis | PKL | a SWI/SWF nuclear-localized chromatin remodeling factor of the CHD3 group |
| *cqOC-C8* | BnaC08g34430D | AT2G23540 | GDSL |  |  |
| *cqOC-C8* | BnaC08g34530D | AT2G23180 | Cuticular Wax Synthesis | CYP96A1 | Midchain Alkane Hydroxylase / Cytochrome P45, 96A |
| *cqOC-C8* | BnaC08g34670D | AT2G22170 | Lipase |  |  |
| *cqOC-C8* | BnaC08g34680D | AT2G22170 | Lipase |  |  |
| *cqOC-C8* | BnaC08g35250D | AT2G22230 | Plastidial Fatty Acid Synthesis | HAD | Hydroxyacyl-ACP Dehydrase |
| *cqOC-C8* | BnaC08g35260D | AT2G22230 | Plastidial Fatty Acid Synthesis | HAD | Hydroxyacyl-ACP Dehydrase |
| *cqOC-C8* | BnaC08g35290D | AT2G22170 | Lipase |  |  |
| *cqOC-C8* | BnaC08g35480D | AT2G21910 | Cuticular Wax Synthesis | CYP96A5 | Midchain Alkane Hydroxylase / Cytochrome P45, 96A |
| *cqOC-C8* | BnaC08g36080D | AT2G20960 | Phospholipase |  |  |
| *cqOC-C8* | BnaC08g36090D | AT2G20950 | Phospholipase |  |  |
| *cqOC-C8* | BnaC08g36120D | AT2G20900 | Lipid Signaling |  | Diacylglycerol Kinase |
| *cqOC-C8* | BnaC08g36170D | AT2G20860 | Mitochondrial Fatty Acid and Lipoic Acid Synthesis | LS or LIP1 | Lipoate Synthase |
| *cqOC-C8* | BnaC08g36500D | AT2G19880 | Sphingolipid Synthesis |  | Glucosylceramide Synthase (UDP-glucose-dependent) |
| *cqOC-C8* | BnaC08g36720D | AT1G19440 | Fatty Acid Elongation and Cuticular Wax Synthesis | KCS4 | Ketoacyl-CoA Synthase |
| *cqOC-C8* | BnaC08g37280D | AT1G18460 | Lipase |  |  |
| *cqOC-C8* | BnaC08g37340D | AT1G18280 | Cuticular Wax Synthesis | LTP type 5 | Lipid Transfer Protein type 5 |
| *cqOC-C8* | BnaC08g37360D | AT1G18280 | Cuticular Wax Synthesis | LTP type 5 | Lipid Transfer Protein type 5 |
| *cqOC-C8* | BnaC08g37370D | AT1G18280 | Cuticular Wax Synthesis | LTP type 5 | Lipid Transfer Protein type 5 |
| *cqOC-C8* | BnaC08g37440D | AT1G76690 | Lipid Signaling |  | Oxo-Phytodienoic Acid Reductase |
| *cqOC-C9-2* | BnaC09g01080D | AT2G38110 | Cutin Synthesis | GPAT6 | Glycerol-3-Phosphate Acyltransferase (endomembrane predicted?) |
| *cqOC-C9-2* | BnaC09g01940D | AT3G28910 | Cuticular Wax Synthesis | MYB3 | Myb Transcription Factors |
| *cqOC-C9-2* | BnaC09g02340D | AT5G48370 | Miscellaneous: lipid related |  | Acyl-CoA Thioesterase |

| **Table S9. Information for acyl-related gene enrichment / prevalence in the CI of SOC-QTLs.** | | | |
| --- | --- | --- | --- |
|  | **Geno_chr^a^** | **QTL_Interval^b^** | **QTL/Chr^c^** |
| **Acyl-Lipid related Genes NO.** | 2476 | 448 | 0.181 |
| **Genome length** | 645.40 | 108.96 | 0.169 |
| **NO. / Mb** | 3.84 | 4.12 | 1.07 |
| **^a^on chromosome in genome** |  |  |  |
| **^b^within confidence interval of SOC-QTLs** | |  |  |
| **^C^ratio of acyl-lipid related genes within the CI of SOC-QTLs and on the chromosome of the genome** | | | |

| **Table S10. Potential candidates related to acyl-lipid metabolism in the SOC-QTL genomic region (major QTLs are bolded, and the potential candidates identified in the overlapping region of SOC-QTLs and AGR are labeled in red)** | | | | | |
| --- | --- | --- | --- | --- | --- |
| **SOC-QTL** | **Gene alias** | ***A. thaliana* Locus** | **ARALIP pathway** | **ARALIP Protein/gene abbrev** | **ARALIP Protein family name/isoform** |
| *cqOC-A1* | BnaA01g12060D | AT4G22240 | Miscellaneous: lipid related |  | Plastid Lipid-associated Protein |
| *cqOC-A1* | BnaA01g12140D | AT4G22330 | Sphingolipid Synthesis | CES1 | Ceramidase |
| *cqOC-A1* | BnaA01g12150D | AT4G22340 | Eukaryotic Phospholipid Synthesis |  | CDP-DAG Synthase |
| *cqOC-A1* | BnaA01g12260D | AT4G22520 | Cuticular Wax Synthesis | LTP type 6 | Lipid Transfer Protein type 6 |
| *cqOC-A1* | BnaA01g12290D | AT4G22550 | Plastidial Glycerolipid, Galactolipid and Sulfolipid Synthesis | LPP &beta; | Phosphatidate Phosphatase |
| *cqOC-A1* | BnaA01g12830D | AT1G73550 | Cuticular Wax Synthesis | LTP type 5 | Lipid Transfer Protein type 5 |
| *cqOC-A1* | BnaA01g13470D | AT4G23850 | Eukaryotic Phospholipid Synthesis | LACS4 | Long-Chain Acyl-CoA Synthetase |
| *cqOC-A1* | BnaA01g13630D | AT4G24160 | Lipase |  | Cardiolipin-Specific Deacylase |
| *cqOC-A1* | BnaA01g13710D | AT4G24230 | Eukaryotic Phospholipid Synthesis | ACBP3 | Acyl CoA Binding Protein; Acyl-CoA Binding Protein |
| *cqOC-A1* | BnaA01g14400D | AT4G25050 | Plastidial Fatty Acid Synthesis | ACP4 | Acyl Carrier Protein |
| *cqOC-A1* | BnaA01g14480D | AT4G25140 | TAG Synthesis |  | Oil-Body Oleosin |
| *cqOC-A1* | BnaA01g14970D | AT4G25750 | Cuticular Wax Synthesis | WBC4 / ABCG4 | ABC Transporter |
| *cqOC-A1* | BnaA01g15140D | AT4G25970 | Eukaryotic Phospholipid Synthesis | PSD3 | Phosphatidylserine Decarboxylase |
| *cqOC-A2-2* | BnaA02g12590D | AT1G66850 | Cuticular Wax Synthesis | LTP type 2 | Lipid Transfer Protein type 2 |
| *cqOC-A2-2* | BnaA02g13230D | AT1G67560 | Lipase |  | Lipoxygenase |
| *cqOC-A2-2* | BnaA02g13270D | AT1G77590 | Plastidial Fatty Acid Synthesis | LACS9 | Long-Chain Acyl-CoA Synthetase (plastidial); Long-Chain Acyl-CoA Synthetase |
| *cqOC-A2-2* | BnaA02g13310D | AT1G67730 | Fatty Acid Elongation and Cuticular Wax Synthesis | KCR1 | Ketoacyl-CoA Reductase |
| *cqOC-A2-2* | BnaA02g13510D | AT1G68000 | Eukaryotic Phospholipid Synthesis | PIS1 | Phosphatidylinositol Synthase |
| ***cqOC-A2-3*** | BnaA02g14460D | AT1G69640 | Sphingolipid Synthesis | SBH1 | Sphingobase C4-Hydroxylase |
| ***cqOC-A2-3*** | BnaA02g15090D | AT1G70670 | TAG Synthesis |  | Caleosin |
| ***cqOC-A2-3*** | BnaA02g15290D | AT1G71010 | Lipid Signaling |  | Phosphatidylinositol-Phosphate Kinase type III |
| ***cqOC-A2-3*** | BnaA02g15690D | AT1G71960 | Cuticular Wax Synthesis | WBC26 / ABCG25 | ABC Transporter |
| ***cqOC-A2-3*** | BnaA02g15770D | AT1G72110 | Cuticular Wax Synthesis |  | Bifunctional Wax Ester Synthase / Diacylglycerol Acyltransferase |
| ***cqOC-A2-3*** | BnaA02g15790D | AT1G72110 | Cuticular Wax Synthesis |  | Bifunctional Wax Ester Synthase / Diacylglycerol Acyltransferase |
| ***cqOC-A2-3*** | BnaA02g16020D | AT1G72520 | Lipase |  | Lipoxygenase |
| ***cqOC-A2-3*** | BnaA02g16070D | AT1G72700 | Miscellaneous: lipid related |  | Translocase |
| ***cqOC-A2-3*** | BnaA02g16200D | AT1G72970 | Cutin Synthesis | HTD | Omega-Hydroxy Fatty Acyl Dehydrogenase; omega-Hydroxy Fatty Acyl Dehydrogenase |
| ***cqOC-A2-3*** | BnaA02g16260D | AT1G73050 | Cutin Synthesis |  | Omega-Hydroxy Fatty Acyl Dehydrogenase; omega-Hydroxy Fatty Acyl Dehydrogenase |
| ***cqOC-A2-3*** | BnaA02g16270D | AT1G73050 | Cutin Synthesis |  | Omega-Hydroxy Fatty Acyl Dehydrogenase; omega-Hydroxy Fatty Acyl Dehydrogenase |
| ***cqOC-A2-3*** | BnaA02g16280D | AT1G73050 | Cutin Synthesis |  | Omega-Hydroxy Fatty Acyl Dehydrogenase; omega-Hydroxy Fatty Acyl Dehydrogenase |
| ***cqOC-A2-3*** | BnaA02g16520D | AT1G73680 | Lipid Signaling |  | &&alpha;;-Dioxygenase-Peroxidase (involved in fatty acid &&alpha;;-oxidation) |
| ***cqOC-A2-3*** | BnaA02g16570D | AT1G73780 | Cuticular Wax Synthesis | LTP type 2 | Lipid Transfer Protein type 2 |
| ***cqOC-A2-3*** | BnaA02g17050D | AT1G74960 | Plastidial Fatty Acid Synthesis | KASII; KASII (fab1) | Ketoacyl-ACP Synthase II |
| ***cqOC-A2-3*** | BnaA02g17090D | AT1G75020 | TAG Synthesis | LPAAT4 | 1-acylglycerol-3-phosphate acyltransferase; 1-Acylglycerol-3-Phosphate Acyltransferase |
| ***cqOC-A2-3*** | BnaA02g17170D | AT1G75020 | TAG Synthesis | LPAAT4 | 1-acylglycerol-3-phosphate acyltransferase; 1-Acylglycerol-3-Phosphate Acyltransferase |
| ***cqOC-A2-3*** | BnaA02g17240D | AT1G47620 | Cuticular Wax Synthesis | CYP96A8 | Midchain Alkane Hydroxylase / Cytochrome P45, 96A |
| ***cqOC-A2-3*** | BnaA02g17580D | AT1G75910 | Lipase |  |  |
| ***cqOC-A2-3*** | BnaA02g17670D | AT1G76680 | Lipid Signaling |  | Oxo-Phytodienoic Acid Reductase |
| ***cqOC-A2-3*** | BnaA02g18240D | AT1G77660 | Lipid Signaling |  | Phosphatidylinositol-Phosphate Kinase type IB |
| ***cqOC-A2-3*** | BnaA02g18410D | AT1G77420 | TAG Degradation |  | Monoacylglycerol Lipase (MAGL) |
| ***cqOC-A2-3*** | BnaA02g18990D | AT3G57310 | Cuticular Wax Synthesis | LTP type 2 | Lipid Transfer Protein type 2 |
| ***cqOC-A2-3*** | BnaA02g19010D | AT1G78690 | Mitochondrial Phospholipid Synthesis |  | Cardiolipin Transacylase |
| ***cqOC-A2-3*** | BnaA02g20580D | AT5G24220 | Lipase |  | Lipid Acylhydrolase-like |
| ***cqOC-A2-3*** | BnaA02g21490D | AT4G31810 | Mitochondrial Fatty Acid and Lipoic Acid Synthesis | | Mitochondrial Enoyl-CoA Hydratase |
| ***cqOC-A2-3*** | BnaA02g21860D | AT4G11030 | Eukaryotic Phospholipid Synthesis | LACS5 | Long-Chain Acyl-CoA Synthetase |
| ***cqOC-A2-3*** | BnaA02g22950D | AT5G42170 | GDSL |  |  |
| ***cqOC-A2-3*** | BnaA02g24400D | AT5G46290 | Plastidial Fatty Acid Synthesis | KASI | Ketoacyl-ACP Synthase I |
| ***cqOC-A2-3*** | BnaA02g24550D | AT2G34690 | Sphingolipid Synthesis | ACD11 | Sphingosine Transfer Protein |
| ***cqOC-A2-3*** | BnaA02g25590D | AT5G47630 | Mitochondrial Fatty Acid and Lipoic Acid Synthesis | | Acyl Carrier Protein |
| ***cqOC-A2-3*** | BnaA02g25680D | AT5G47730 | Miscellaneous: lipid related |  | Sec14-like Protein |
| *cqOC-A3-1* | BnaA03g08520D | AT3G44540 | Cuticular Wax Synthesis | AlcFAR4 | Alcohol-forming Fatty Acyl-CoA Reductase (ER); Alcohol-forming Fatty Acyl-CoA Reductase |
| *cqOC-A3-1* | BnaA03g08950D | AT5G60340 | Mitochondrial Fatty Acid and Lipoic Acid Synthesis | HAD | Hydroxyacyl-ACP Dehydrase |
| *cqOC-A3-1* | BnaA03g09530D | AT5G59320 | Cuticular Wax Synthesis | LTP type 1 | Lipid Transfer Protein type 1 |
| *cqOC-A3-1* | BnaA03g09540D | AT5G59320 | Cuticular Wax Synthesis | LTP type 1 | Lipid Transfer Protein type 1 |
| *cqOC-A3-2* | BnaA03g11410D | AT5G55450 | Cuticular Wax Synthesis | LTP type 3 | Lipid Transfer Protein type 3 |
| *cqOC-A3-2* | BnaA03g11420D | AT5G55410 | Cuticular Wax Synthesis | LTP type 3 | Lipid Transfer Protein type 3 |
| *cqOC-A3-2* | BnaA03g11440D | AT5G55370 | Cuticular Wax Synthesis |  | Wax Synthase |
| *cqOC-A3-2* | BnaA03g11450D | AT5G55340 | Cuticular Wax Synthesis |  | Wax Synthase |
| *cqOC-A3-2* | BnaA03g11460D | AT5G55350 | Cuticular Wax Synthesis |  | Wax Synthase |
| *cqOC-A3-2* | BnaA03g11470D | AT5G55360 | Cuticular Wax Synthesis |  | Wax Synthase |
| *cqOC-A3-2* | BnaA03g11610D | AT5G55050 | GDSL |  |  |
| *cqOC-A3-2* | BnaA03g11620D | AT5G55050 | GDSL |  |  |
| *cqOC-A3-3* | BnaA03g29710D | AT3G06860 | beta-Oxidation | MFP2 | Multifunctional Protein |
| *cqOC-A3-3* | BnaA03g30020D | AT3G07450 | Cuticular Wax Synthesis | LTP type 3 | Lipid Transfer Protein type 3 |
| *cqOC-A3-3* | BnaA03g30260D | AT3G07960 | Lipid Signaling |  | Phosphatidylinositol-Phosphate Kinase type IB |
| *cqOC-A3-3* | BnaA03g30270D | AT3G07960 | Lipid Signaling |  | Phosphatidylinositol-Phosphate Kinase type IB |
| *cqOC-A3-3* | BnaA03g31600D | AT3G11170 | Plastidial Glycerolipid, Galactolipid and Sulfolipid Synthesis | FAD7 | Linoleate Desaturase |
| *cqOC-A3-3* | BnaA03g32450D | AT3G12800 | beta-Oxidation |  | Dienoyl-CoA Reductase |
| *cqOC-A3-4* | BnaA03g42860D | AT4G16820 | TAG degradation |  | Acylhydrolase (DAD1-like) |
| *cqOC-A3-5* | BnaA03g44190D | AT4G19860 | Phospholipase |  | Phospholipid : Acyl acceptor Acyltransferase |
| *cqOC-A3-5* | BnaA03g44270D | AT4G19860 | Phospholipase |  | Phospholipid : Acyl acceptor Acyltransferase |
| *cqOC-A3-5* | BnaA03g44730D | AT4G21540 | Sphingolipid Synthesis |  | Long Chain Base Kinase |
| *cqOC-A3-5* | BnaA03g45270D | AT4G22300 | Lipid Signaling |  | Lysophospholipase |
| *cqOC-A3-5* | BnaA03g45300D | AT4G22330 | Sphingolipid Synthesis | CES1 | Ceramidase |
| *cqOC-A3-5* | BnaA03g45330D | AT4G22490 | Cuticular Wax Synthesis | LTP type 6 | Lipid Transfer Protein type 6 |
| *cqOC-A8-2* | BnaA08g10210D | AT4G22330 | Sphingolipid Synthesis | CES1 | Ceramidase |
| *cqOC-A8-2* | BnaA08g10330D | AT4G22520 | Cuticular Wax Synthesis | LTP type 6 | Lipid Transfer Protein type 6 |
| *cqOC-A8-2* | BnaA08g10850D | AT4G35110 | Phospholipase |  |  |
| *cqOC-A8-2* | BnaA08g11140D | AT4G34510 | Fatty Acid Elongation and Cuticular Wax Synthesis | KCS17 | Ketoacyl-CoA Synthase |
| *cqOC-A8-2* | BnaA08g11440D | AT4G33790 | Cuticular Wax Synthesis | AlcFAR3 (CER4); AlcFAR3/CER4 | Alcohol-forming Fatty Acyl-CoA Reductase (ER); Alcohol-forming Fatty Acyl-CoA Reductase |
| *cqOC-A8-2* | BnaA08g11640D | AT4G34050 | Aromatic Suberin Synthesis |  | Caffeoyl-CoA O-Methyltransferase |
| *cqOC-A8-2* | BnaA08g11810D | AT4G33355 | Cuticular Wax Synthesis | LTP type 1 | Lipid Transfer Protein type 1 |
| *cqOC-A8-3* | BnaA08g12350D | AT4G31810 | Mitochondrial Fatty Acid and Lipoic Acid Synthesis | | Mitochondrial Enoyl-CoA Hydratase |
| *cqOC-A8-3* | BnaA08g12720D | AT4G31050 | Plastidial Fatty Acid Synthesis |  | Lipoyltransferase |
| *cqOC-A8-3* | BnaA08g12730D | AT4G31020 | Phospholipase |  |  |
| *cqOC-A8-3* | BnaA08g12780D | AT4G30950 | Plastidial Glycerolipid, Galactolipid and Sulfolipid Synthesis | FAD6 | Oleate Desaturase |
| *cqOC-A8-3* | BnaA08g12800D | AT4G30950 | Plastidial Glycerolipid, Galactolipid and Sulfolipid Synthesis | FAD6 | Oleate Desaturase |
| *cqOC-A8-3* | BnaA08g12850D | AT4G30880 | Cuticular Wax Synthesis | LTP type 3 | Lipid Transfer Protein type 3 |
| *cqOC-A8-3* | BnaA08g13200D | AT4G30140 | GDSL |  |  |
| *cqOC-A8-3* | BnaA08g13220D | AT4G30140 | GDSL |  |  |
| *cqOC-A8-3* | BnaA08g13410D | AT4G29460 | Phospholipase |  | Phospholipase A2 |
| *cqOC-A8-3* | BnaA08g13520D | AT4G29070 | Phospholipase |  | Phospholipase A2 |
| *cqOC-A8-3* | BnaA08g13870D | AT2G20900 | Lipid Signaling |  | Diacylglycerol Kinase |
| *cqOC-A8-3* | BnaA08g14190D | AT4G27030 | Plastidial Glycerolipid, Galactolipid and Sulfolipid Synthesis | FAD4 | Phosphatidylglycerol Desaturase |
| *cqOC-A8-3* | BnaA08g14200D | AT4G27030 | Plastidial Glycerolipid, Galactolipid and Sulfolipid Synthesis | FAD4 | Phosphatidylglycerol Desaturase |
| *cqOC-A8-6* | BnaA08g14540D | AT4G25140 | TAG Synthesis |  | Oil-Body Oleosin |
| *cqOC-A8-6* | BnaA08g14550D | AT4G25050 | Plastidial Fatty Acid Synthesis | ACP4 | Acyl Carrier Protein |
| *cqOC-A9-2* | BnaA09g07080D | AT5G65110 | beta-Oxidation | ACX2 | Acyl-CoA Oxidase |
| *cqOC-A9-2* | BnaA09g07600D | AT5G67050 | TAG Degradation |  | Triacylglycerol Lipase (TAGL); Lipid Acylhydrolase-like |
| *cqOC-A9-2* | BnaA09g08140D | AT5G66020 | Lipid Signaling |  | Sac domain-containing Phosphoinositide Phosphatase |
| *cqOC-A9-2* | BnaA09g08200D | AT2G11810 | Plastidial Glycerolipid, Galactolipid and Sulfolipid Synthesis | MGD3 | Monogalactosyldiacylglycerol Synthase |
| *cqOC-A9-4* | BnaA09g12230D | AT2G46500 | Lipid Signaling |  | Phosphatidylinositol-4-Kinase &gamma; |
| *cqOC-A9-4* | BnaA09g12890D | AT1G62640 | Plastidial Fatty Acid Synthesis | KASIII | Ketoacyl-ACP Synthase III |
| *cqOC-A9-4* | BnaA09g12940D | AT1G63050 | TAG Synthesis |  | 1-acylglycerol-3-phosphocholine Acyltransferase |
| *cqOC-A9-4* | BnaA09g13270D | AT1G62430 | Eukaryotic Phospholipid Synthesis | CDS1 | CDP-DAG Synthase |
| *cqOC-A9-4* | BnaA09g13590D | AT1G61850 | Galactolipid degradation | AtPLA1 |  |
| *cqOC-A9-4* | BnaA09g14090D | AT1G60890 | Lipid Signaling |  | Phosphatidylinositol-Phosphate Kinase type IB |
| *cqOC-A9-4* | BnaA09g14140D | AT1G60490 | Lipid Signaling |  | Phosphatidylinositol-3-Kinase |
| *cqOC-A9-4* | BnaA09g14610D | AT1G58725 | GDSL |  |  |
| *cqOC-A9-4* | BnaA09g15080D | AT1G57750 | Cuticular Wax Synthesis | CYP96A15/MAH1 | Midchain Alkane Hydroxylase / Cytochrome P45, 96A; Secondary Alcohol Dehydrogenase |
| *cqOC-A9-4* | BnaA09g15090D | AT1G57750 | Cuticular Wax Synthesis | CYP96A15/MAH1 | Midchain Alkane Hydroxylase / Cytochrome P45, 96A; Secondary Alcohol Dehydrogenase |
| *cqOC-A9-4* | BnaA09g15100D | AT1G57750 | Cuticular Wax Synthesis | CYP96A15/MAH1 | Midchain Alkane Hydroxylase / Cytochrome P45, 96A; Secondary Alcohol Dehydrogenase |
| *cqOC-A9-4* | BnaA09g15130D | AT1G57750 | Cuticular Wax Synthesis | CYP96A15/MAH1 | Midchain Alkane Hydroxylase / Cytochrome P45, 96A; Secondary Alcohol Dehydrogenase |
| *cqOC-A9-4* | BnaA09g15160D | AT1G57750 | Cuticular Wax Synthesis | CYP96A15/MAH1 | Midchain Alkane Hydroxylase / Cytochrome P45, 96A; Secondary Alcohol Dehydrogenase |
| *cqOC-A9-4* | BnaA09g15250D | AT1G56670 | GDSL |  |  |
| *cqOC-A9-4* | BnaA09g15550D | AT1G58725 | GDSL |  |  |
| *cqOC-A9-4* | BnaA09g15560D | AT1G58725 | GDSL |  |  |
| *cqOC-A9-4* | BnaA09g15670D | AT5G03610 | GDSL |  |  |
| *cqOC-A9-4* | BnaA09g15750D | AT5G42870 | TAG Synthesis |  | Phosphatidate Phosphatase (ER); Phosphatidate Phosphatase |
| *cqOC-A9-5* | BnaA09g34250D | AT3G54320 | Plastidial Fatty Acid Synthesis | WRI1 | AP2/ERWEBP Transcription Factors |
| *cqOC-A9-5* | BnaA09g34910D | AT3G55290 | Plastidial Fatty Acid Synthesis |  | Ketoacyl-ACP Reductase |
| *cqOC-A9-5* | BnaA09g34930D | AT3G55360 | Fatty Acid Elongation and Cuticular Wax Synthesis | ECR; ECR/CER1 | Enoyl-CoA Reductase |
| *cqOC-A9-5* | BnaA09g35480D | AT3G55940 | Phospholipase |  | Phosphoinositide-specific Phospholipase C |
| *cqOC-A9-5* | BnaA09g35520D | AT3G56060 | Cutin Synthesis |  | Omega-Hydroxy Fatty Acyl Dehydrogenase; omega-Hydroxy Fatty Acyl Dehydrogenase |
| *cqOC-A9-5* | BnaA09g35530D | AT3G56060 | Cutin Synthesis |  | Omega-Hydroxy Fatty Acyl Dehydrogenase; omega-Hydroxy Fatty Acyl Dehydrogenase |
| *cqOC-A9-6* | BnaA09g40500D | AT2G26420 | Lipid Signaling |  | Phosphatidylinositol-Phosphate Kinase type IB |
| *cqOC-A9-7* | BnaA09g40640D | AT2G26250 | Fatty Acid Elongation and Cuticular Wax Synthesis | KCS1/FDH | Ketoacyl-CoA Synthase |
| *cqOC-A9-8* | BnaA09g42120D | AT2G23180 | Cuticular Wax Synthesis | CYP96A1 | Midchain Alkane Hydroxylase / Cytochrome P45, 96A |
| *cqOC-A9-8* | BnaA09g42230D | AT2G22170 | Lipase |  |  |
| ***cqOC-A9-9*** | BnaA09g44630D | AT1G18280 | Cuticular Wax Synthesis | LTP type 5 | Lipid Transfer Protein type 5 |
| ***cqOC-A9-10*** | BnaA09g45720D | AT1G14190 | Cutin Synthesis |  | Omega-Hydroxy Fatty Acyl Dehydrogenase; omega-Hydroxy Fatty Acyl Dehydrogenase |
| ***cqOC-A9-10*** | BnaA09g46200D | AT1G13580 | Sphingolipid Synthesis | LAG13 | Ceramide Synthase |
| ***cqOC-A9-10*** | BnaA09g46210D | AT1G13560 | Eukaryotic Phospholipid Synthesis |  | Diacylglycerol Cholinephosphotransferase |
| ***cqOC-A9-10*** | BnaA09g46440D | AT1G13210 | Miscellaneous: lipid related |  | Translocase |
| ***cqOC-A9-10*** | BnaA09g47830D | AT1G10900 | Lipid Signaling |  | Phosphatidylinositol-Phosphate Kinase type IB |
| ***cqOC-A9-10*** | BnaA09g48190D | AT1G59820 | Miscellaneous: lipid related |  | Translocase |
| ***cqOC-A9-10*** | BnaA09g48250D | AT5G35360 | Plastidial Fatty Acid Synthesis | BC (Subunit of Heteromeric ACCase) | Biotin Carboxylase of Heteromeric ACCase |
| ***cqOC-A9-10*** | BnaA09g48610D | AT1G08980 | Lipid Signaling |  | Fatty Acid Amide Hydrolase |
| ***cqOC-A9-10*** | BnaA09g50070D | AT1G06090 | Miscellaneous: lipid related |  | Acyl-CoA Desaturase-like |
| ***cqOC-A9-10*** | BnaA09g50080D | AT1G06090 | Miscellaneous: lipid related |  | Acyl-CoA Desaturase-like |
| ***cqOC-A9-10*** | BnaA09g50250D | AT1G05630 | Lipid Signaling |  | Phosphoinositide 5-Phosphatase Type II |
| ***cqOC-A9-10*** | BnaA09g50430D | AT5G65158 | Lipase |  |  |
| ***cqOC-A9-10*** | BnaA09g50730D | AT1G04010 | Miscellaneous: lipid related |  | Phospholipid : Acyl acceptor Acyltransferase |
| ***cqOC-A9-10*** | BnaA09g51230D | AT1G02660 | Lipid acylhydrolase |  | Lipid Acylhydrolase-like |
| *cqOC-A9-11* | BnaA09g51530D | AT1G01120 | Fatty Acid Elongation and Cuticular Wax Synthesis | KCS1 | Ketoacyl-CoA Synthase |
| *cqOC-A10-2* | BnaA10g02340D | AT1G04010 | Miscellaneous: lipid related |  | Phospholipid : Acyl acceptor Acyltransferase |
| *cqOC-A10-2* | BnaA10g02480D | AT1G04220 | Fatty Acid Elongation and Cuticular Wax Synthesis | KCS2 (DAISY); KCS2 | Ketoacyl-CoA Synthase |
| *cqOC-A10-2* | BnaA10g02780D | AT1G04640 | Mitochondrial Fatty Acid and Lipoic Acid Synthesis | LT | Lipoyltransferase |
| *cqOC-A10-2* | BnaA10g03520D | AT1G05790 | Lipase |  |  |
| *cqOC-A10-2* | BnaA10g03530D | AT1G05800 | Galactolipid degradation | DGL | Acylhydrolase (DAD1-like) |
| *cqOC-A10-2* | BnaA10g03830D | AT1G05630 | Lipid Signaling |  | Phosphoinositide 5-Phosphatase Type II |
| *cqOC-A10-2* | BnaA10g04030D | AT1G06080 | Miscellaneous: lipid related |  | Acyl-CoA Desaturase-like |
| *cqOC-A10-2* | BnaA10g04040D | AT1G06090 | Miscellaneous: lipid related |  | Acyl-CoA Desaturase-like |
| *cqOC-A10-2* | BnaA10g04260D | AT1G06520 | Cutin Synthesis | GPAT1 | Glycerol-3-Phosphate Acyltransferase (mitochondrial); Glycerol-3-Phosphate Acyltransferase |
| *cqOC-A10-2* | BnaA10g04450D | AT2G24560 | GDSL |  |  |
| *cqOC-A10-2* | BnaA10g04680D | AT1G07230 | Plastidial Glycerolipid, Galactolipid and Sulfolipid Synthesis | NPC1 (Non specific) | Phospholipase C (Non specific) |
| *cqOC-A10-2* | BnaA10g05760D | AT5G51210 | TAG Synthesis |  | Oil-Body Oleosin |
| *cqOC-A10-2* | BnaA10g06020D | AT5G50690 | TAG Synthesis |  | Steroleosin |
| *cqOC-A10-2* | BnaA10g06730D | AT5G12420 | Cuticular Wax Synthesis |  | Bifunctional Wax Ester Synthase / Diacylglycerol Acyltransferase |
| *cqOC-A10-2* | BnaA10g07590D | AT5G52160 | Cuticular Wax Synthesis | LTP type 3 | Lipid Transfer Protein type 3 |
| *cqOC-A10-2* | BnaA10g07670D | AT5G51950 | Cutin Synthesis |  | Omega-Hydroxy Fatty Acyl Dehydrogenase; omega-Hydroxy Fatty Acyl Dehydrogenase |
| *cqOC-A10-2* | BnaA10g09280D | AT3G53510 | Cuticular Wax Synthesis | WBC2 / ABCG2 | ABC Transporter |
| *cqOC-A10-2* | BnaA10g09310D | AT5G55050 | GDSL |  |  |
| *cqOC-A10-2* | BnaA10g09320D | AT5G55050 | GDSL |  |  |
| *cqOC-A10-2* | BnaA10g09480D | AT5G55240 | TAG Synthesis |  | Caleosin |
| *cqOC-A10-2* | BnaA10g09530D | AT5G55340 | Cuticular Wax Synthesis |  | Wax Synthase |
| *cqOC-A10-2* | BnaA10g09590D | AT5G55460 | Cuticular Wax Synthesis | LTP type 3 | Lipid Transfer Protein type 3 |
| *cqOC-A10-2* | BnaA10g09610D | AT5G55380 | Cuticular Wax Synthesis |  | Wax Synthase |
| *cqOC-A10-2* | BnaA10g09620D | AT5G55350 | Cuticular Wax Synthesis |  | Wax Synthase |
| *cqOC-A10-2* | BnaA10g09630D | AT5G55360 | Cuticular Wax Synthesis |  | Wax Synthase |
| *cqOC-A10-2* | BnaA10g09640D | AT5G55410 | Cuticular Wax Synthesis | LTP type 3 | Lipid Transfer Protein type 3 |
| *cqOC-A10-2* | BnaA10g11040D | AT5G57020 | Miscellaneous: lipid related |  | Protein N-Myristoyltransferase |
| *cqOC-A10-2* | BnaA10g11230D | AT5G57190 | Eukaryotic Phospholipid Synthesis | PSD2 | Phosphatidylserine Decarboxylase |
| *cqOC-A10-2* | BnaA10g11680D | AT5G57800 | Cuticular Wax Synthesis | CER3/WAX2/YRE/FLP1 | CER3 Protein |
| *cqOC-A10-2* | BnaA10g13450D | AT5G60620 | TAG Synthesis | GPAT9? (mammalian homologue) | Glycerol-3-Phosphate Acyltransferase (mammalian homologue) |
| *cqOC-A10-2* | BnaA10g13850D | AT5G22500 | Cuticular Wax Synthesis | AlcFAR1 | Alcohol-forming Fatty Acyl-CoA Reductase |
| *cqOC-A10-2* | BnaA10g13920D | AT3G44540 | Cuticular Wax Synthesis | AlcFAR4 | Alcohol-forming Fatty Acyl-CoA Reductase (ER); Alcohol-forming Fatty Acyl-CoA Reductase |
| *cqOC-A10-2* | BnaA10g14700D | AT5G20840 | Lipid Signaling |  | Sac domain-containing Phosphoinositide Phosphatase |
| *cqOC-A10-2* | BnaA10g15720D | AT5G19410 | Cuticular Wax Synthesis | WBC24 / ABCG23 | ABC Transporter |
| *cqOC-A10-2* | BnaA10g15820D | AT5G19290 | TAG Degradation |  | Monoacylglycerol Lipase (MAGL); Lysophospholipase |
| *cqOC-A10-2* | BnaA10g15860D | AT5G19200 | Sphingolipid Synthesis |  | Ketosphinganine Reductase |
| *cqOC-A10-2* | BnaA10g16330D | AT5G18630 | TAG Degradation |  | Triacylglycerol Lipase (TAGL) |
| *cqOC-A10-2* | BnaA10g17000D | AT5G17780 | Lipid Signaling |  | Lysophospholipase |
| *cqOC-A10-2* | BnaA10g17650D | AT1G76680 | Lipid Signaling |  | Oxo-Phytodienoic Acid Reductase |
| *cqOC-A10-2* | BnaA10g17730D | AT2G03140 | Lipase |  |  |
| *cqOC-A10-2* | BnaA10g18080D | AT3G02630 | Plastidial Fatty Acid Synthesis | DES5 | Stearoyl-ACP Desaturase |
| *cqOC-A10-2* | BnaA10g18090D | AT5G16230 | Plastidial Fatty Acid Synthesis | DES3 | Stearoyl-ACP Desaturase |
| *cqOC-A10-2* | BnaA10g18180D | AT5G16120 | TAG Degradation |  | Monoacylglycerol Lipase (MAGL) |
| *cqOC-A10-2* | BnaA10g18250D | AT5G16080 | Lipase |  |  |
| *cqOC-A10-2* | BnaA10g18680D | AT5G15530 | Plastidial Fatty Acid Synthesis | BCCP2 (Subunit of Heteromeric ACCase) | Biotin Carboxyl Carrier Protein of Heteromeric ACCase |
| *cqOC-A10-2* | BnaA10g18720D | AT3G58210 | Phospholipase |  |  |
| *cqOC-A10-2* | BnaA10g19400D | AT5G14310 | Lipase |  |  |
| *cqOC-A10-2* | BnaA10g19480D | AT5G14180 | TAG Degradation |  | Triacylglycerol Lipase (TAGL) |
| *cqOC-A10-1* | BnaA10g19780D | AT5G13580 | Cuticular Wax Synthesis | WBC6; WBC6 / ABCG6 | ABC transporter; ABC Transporter |
| *cqOC-A10-1* | BnaA10g20920D | AT5G11650 | TAG Degradation |  | Monoacylglycerol Lipase (MAGL) |
| *cqOC-A10-1* | BnaA10g20970D | AT1G77590 | Plastidial Fatty Acid Synthesis | LACS9 | Long-Chain Acyl-CoA Synthetase (plastidial); Long-Chain Acyl-CoA Synthetase |
| *cqOC-A10-1* | BnaA10g21780D | AT5G10480 | Fatty Acid Elongation and Cuticular Wax Synthesis | HACD; HCD/PAS2 | Hydroxyacyl-CoA Dehydratase |
| *cqOC-A10-1* | BnaA10g23000D | AT5G08460 | GDSL |  |  |
| *cqOC-A10-1* | BnaA10g23290D | AT5G08030 | Miscellaneous: lipid related |  | Glycerophosphoryl Diester Phosphodiesterase |
| *cqOC-C2-1* | BnaC02g35410D | AT1G13580 | Sphingolipid Synthesis | LAG13 | Ceramide Synthase |
| *cqOC-C2-1* | BnaC02g35520D | AT3G25585 | TAG Synthesis | DAG-CPT | Diacylglycerol Cholinephosphotransferase |
| *cqOC-C2-1* | BnaC02g35530D | AT3G25610 | Miscellaneous: lipid related |  | Translocase |
| *cqOC-C2-2* | BnaC02g36400D | AT3G26840 | Lipase |  |  |
| *cqOC-C2-2* | BnaC02g36410D | AT3G26840 | Lipase |  |  |
| *cqOC-C2-2* | BnaC02g36440D | AT3G26840 | Lipase |  |  |
| *cqOC-C2-2* | BnaC02g37030D | AT3G27660 | TAG Synthesis |  | Oil-Body Oleosin |
| *cqOC-C2-2* | BnaC02g37590D | AT3G28910 | Cuticular Wax Synthesis | MYB3 | Myb Transcription Factors |
| *cqOC-C2-2* | BnaC02g37710D | AT5G38170 | Cuticular Wax Synthesis | LTP type 2 | Lipid Transfer Protein type 2 |
| *cqOC-C2-3* | BnaC02g38800D | AT5G48880 | beta-Oxidation | PKT1 | Ketoacyl-CoA Thiolase |
| *cqOC-C2-3* | BnaC02g39080D | AT5G49460 | Cuticular Wax Synthesis | ACLB-2 | ATP Citrate Lyase B subunit |
| *cqOC-C2-3* | BnaC02g39270D | AT5G29560 | TAG Synthesis |  | Caleosin |
| *cqOC-C2-3* | BnaC02g39790D | AT5G27630 | Eukaryotic Phospholipid Synthesis | ACBP5 | Acyl CoA Binding Protein |
| *cqOC-C3-1* | BnaC03g00360D | AT5G01220 | Plastidial Glycerolipid, Galactolipid and Sulfolipid Synthesis | SQD2 | UDP-sulfoquinovose:DAG sulfoquinovosyltransferase |
| *cqOC-C3-1* | BnaC03g01340D | AT5G03610 | GDSL |  |  |
| *cqOC-C3-1* | BnaC03g01430D | AT5G03810 | GDSL |  |  |
| *cqOC-C3-1* | BnaC03g01520D | AT5G04040 | TAG Degradation | SDP1 | Triacylglycerol Lipase (TAGL); Acyl-Hydrolase (Patatin-like) |
| *cqOC-C3-1* | BnaC03g01530D | AT5G04040 | TAG Degradation | SDP1 | Triacylglycerol Lipase (TAGL); Acyl-Hydrolase (Patatin-like) |
| *cqOC-C3-1* | BnaC03g01830D | AT5G04930 | Lipid Trafficking | ALA1 | Aminophospholipid ATPase; Translocase |
| *cqOC-C3-1* | BnaC03g02910D | AT5G62200 | Lipase |  |  |
| *cqOC-C3-1* | BnaC03g03140D | AT5G07530 | TAG Synthesis |  | Pollen-surface Oleosin |
| *cqOC-C3-1* | BnaC03g03150D | AT5G07550 | TAG Synthesis |  | Pollen-surface Oleosin |
| *cqOC-C3-2* | BnaC03g05590D | AT5G12420 | Cuticular Wax Synthesis |  | Bifunctional Wax Ester Synthase / Diacylglycerol Acyltransferase |
| *cqOC-C3-2* | BnaC03g06380D | AT5G14310 | Lipase |  |  |
| *cqOC-C3-2* | BnaC03g06650D | AT5G14930 | TAG Degradation | SAG11 | Triacylglycerol Lipase (TAGL); Lipid Acylhydrolase-like |
| *cqOC-C3-2* | BnaC03g06660D | AT5G14930 | TAG Degradation | SAG11 | Triacylglycerol Lipase (TAGL); Lipid Acylhydrolase-like |
| *cqOC-C3-2* | BnaC03g06680D | AT5G14930 | TAG Degradation | SAG11 | Triacylglycerol Lipase (TAGL); Lipid Acylhydrolase-like |
| *cqOC-C3-2* | BnaC03g07000D | AT5G15530 | Plastidial Fatty Acid Synthesis | BCCP2 (Subunit of Heteromeric ACCase) | Biotin Carboxyl Carrier Protein of Heteromeric ACCase |
| *cqOC-C3-3* | BnaC03g10300D | AT5G20840 | Lipid Signaling |  | Sac domain-containing Phosphoinositide Phosphatase |
| *cqOC-C3-5* | BnaC03g65000D | AT4G22550 | Plastidial Glycerolipid, Galactolipid and Sulfolipid Synthesis | LPP &beta; | Phosphatidate Phosphatase |
| *cqOC-C3-5* | BnaC03g65690D | AT4G34930 | Phospholipase |  | Glycosylphosphatidylinositol-specific Phospholipase C |
| *cqOC-C3-5* | BnaC03g65700D | AT4G34920 | Phospholipase |  | Glycosylphosphatidylinositol-specific Phospholipase C |
| *cqOC-C3-6* | BnaC03g65980D | AT4G34520 | Fatty Acid Elongation and Cuticular Wax Synthesis | FAE1 | Ketoacyl-CoA Synthase |
| *cqOC-C3-6* | BnaC03g66150D | AT4G11850 | Phospholipase |  | Phospholipase D &gamma; |
| *cqOC-C3-6* | BnaC03g66380D | AT4G33790 | Cuticular Wax Synthesis | AlcFAR3 (CER4); AlcFAR3/CER4 | Alcohol-forming Fatty Acyl-CoA Reductase (ER); Alcohol-forming Fatty Acyl-CoA Reductase |
| *cqOC-C3-7* | BnaC03g67370D | AT4G31810 | Mitochondrial Fatty Acid and Lipoic Acid Synthesis | | Mitochondrial Enoyl-CoA Hydratase |
| *cqOC-C3-7* | BnaC03g67820D | AT4G30950 | Plastidial Glycerolipid, Galactolipid and Sulfolipid Synthesis | FAD6 | Oleate Desaturase |
| *cqOC-C3-7* | BnaC03g68070D | AT4G30140 | GDSL |  |  |
| *cqOC-C3-7* | BnaC03g68610D | AT1G08980 | Lipid Signaling |  | Fatty Acid Amide Hydrolase |
| ***cqOC-C5-3*** | **BnaC05g15620D** | **AT1G20130** | **GDSL** |  |  |
| ***cqOC-C5-3*** | **BnaC05g15630D** | **AT1G20132** | **GDSL** |  |  |
| ***cqOC-C5-3*** | **BnaC05g17280D** | **AT1G21920** | **Lipid Signaling** |  | **Phosphatidylinositol-Phosphate Kinase type IB** |
| ***cqOC-C5-4*** | **BnaC05g21070D** | **AT1G24470** | **Fatty Acid Elongation and Cuticular Wax Synthesis** | **KCR2** | **Ketoacyl-CoA Reductase** |
| ***cqOC-C5-4*** | **BnaC05g21140D** | **AT1G24360** | **Plastidial Fatty Acid Synthesis** |  | **Ketoacyl-ACP Reductase** |
| ***cqOC-C5-4*** | **BnaC05g21950D** | **AT1G28580** | **GDSL** |  |  |
| ***cqOC-C5-4*** | **BnaC05g21980D** | **AT1G28580** | **GDSL** |  |  |
| ***cqOC-C5-4*** | **BnaC05g23260D** | **AT1G30370** | **Lipase** |  | **Acylhydrolase (DAD1-like)** |
| ***cqOC-C5-4*** | **BnaC05g24040D** | **AT1G15110** | **Eukaryotic Phospholipid Synthesis** |  | **Base-Exchange-type Phosphatidylserine Synthase** |
| ***cqOC-C5-4*** | **BnaC05g24510D** | **AT5G16390** | **Plastidial Fatty Acid Synthesis** | **BCCP1 (Subunit of Heteromeric ACCase)** | **Biotin Carboxyl Carrier Protein of Heteromeric ACCase** |
| *cqOC-C5-5* | BnaC05g13800D | AT1G17840 | Cuticular Wax Synthesis | ABCG11/WBC11/DES/COF1; ABCG11/WBC11; WBC11 / ABCG11 / DSO/COF1 | ABC Transporter; ABC transporter |
| *cqOC-C5-5* | BnaC05g14030D | AT1G18280 | Cuticular Wax Synthesis | LTP type 5 | Lipid Transfer Protein type 5 |
| *cqOC-C5-5* | BnaC05g14110D | AT1G73890 | Cuticular Wax Synthesis | LTP type 5 | Lipid Transfer Protein type 5 |
| *cqOC-C5-5* | BnaC05g41970D | AT3G11210 | GDSL |  |  |
| *cqOC-C5-5* | BnaC05g42010D | AT3G11170 | Plastidial Glycerolipid, Galactolipid and Sulfolipid Synthesis | FAD7 | Linoleate Desaturase |
| *cqOC-C5-5* | BnaC05g42450D | AT3G10570 | Cutin Synthesis | CYP77A6 | Fatty Acyl in-chain Hydroxylase (CYP77A) |
| *cqOC-C5-5* | BnaC05g42630D | AT3G14270 | Lipid Signaling |  | Phosphatidylinositol-Phosphate Kinase type III |
| *cqOC-C5-5* | BnaC05g42730D | AT3G10370 | Mitochondrial Phospholipid Synthesis | SDP6 | FAD-dependent Glycerol-3-Phosphate Dehydrogenase |
| *cqOC-C5-7* | BnaC05g43390D | AT3G09560 | TAG Synthesis |  | Phosphatidate Phosphatase (ER); Phosphatidate Phosphatase |
| *cqOC-C5-7* | BnaC05g43760D | AT3G08770 | Cuticular Wax Synthesis | LTP type 1 | Lipid Transfer Protein type 1 |
| *cqOC-C5-7* | BnaC05g44270D | AT3G07690 | TAG Synthesis |  | NAD-dependent Glycerol-3-Phosphate Dehydrogenase |
| *cqOC-C5-7* | BnaC05g44510D | AT3G07450 | Cuticular Wax Synthesis | LTP type 3 | Lipid Transfer Protein type 3 |
| *cqOC-C5-7* | BnaC05g44540D | AT3G07400 | Lipid acylhydrolase |  | Lipid Acylhydrolase-like |
| *cqOC-C5-7* | BnaC05g45070D | AT3G06860 | beta-Oxidation | MFP2 | Multifunctional Protein |
| *cqOC-C5-7* | BnaC05g45760D | AT3G06060 | Sphingolipid Synthesis |  | Ketosphinganine Reductase |
| *cqOC-C5-7* | BnaC05g45860D | AT3G05970 | beta-Oxidation | LACS6 | Long-Chain Acyl-CoA Synthetase (peroxisomal) |
| *cqOC-C5-7* | BnaC05g46370D | AT3G05420 | Eukaryotic Phospholipid Synthesis | ACBP4 | Acyl CoA Binding Protein; Acyl-CoA Binding Protein |
| *cqOC-C5-7* | BnaC05g47350D | AT3G04290 | GDSL |  |  |
| *cqOC-C6-2* | BnaC06g16040D | AT3G56700 | Cuticular Wax Synthesis | AlcFAR6 | Alcohol-forming Fatty Acyl-CoA Reductase |
| *cqOC-C6-2* | BnaC06g16060D | AT3G56700 | Cuticular Wax Synthesis | AlcFAR6 | Alcohol-forming Fatty Acyl-CoA Reductase |
| *cqOC-C6-2* | BnaC06g16390D | AT3G58270 | Phospholipase |  |  |
| *cqOC-C6-2* | BnaC06g16550D | AT3G58320 | Phospholipase |  |  |
| *cqOC-C6-2* | BnaC06g16600D | AT3G58490 | Plastidial Glycerolipid, Galactolipid and Sulfolipid Synthesis | LPP &delta; | Long Chain Base 1-Phosphate Phosphatase; Phosphatidate Phosphatase |
| *cqOC-C6-2* | BnaC06g17540D | AT3G59770 | Lipid Signaling |  | Sac domain-containing Phosphoinositide Phosphatase |
| *cqOC-C6-2* | BnaC06g17800D | AT3G60500 | Cuticular Wax Synthesis | CER7 | CER7 Protein involved in wax synthesis |
| *cqOC-C6-2* | BnaC06g17810D | AT3G60510 | Mitochondrial Fatty Acid and Lipoic Acid Synthesis | | Mitochondrial Enoyl-CoA Hydratase |
| *cqOC-C6-3/4/5/6/7/8/9/10* | BnaC06g20770D | AT1G77660 | Lipid Signaling |  | Phosphatidylinositol-Phosphate Kinase type IB |
| *cqOC-C6-3/4/5/6/7/8/9/10* | BnaC06g20910D | AT1G77590 | Plastidial Fatty Acid Synthesis | LACS9 | Long-Chain Acyl-CoA Synthetase (plastidial); Long-Chain Acyl-CoA Synthetase |
| *cqOC-C6-3/4/5/6/7/8/9/10* | BnaC06g21010D | AT1G77420 | TAG Degradation |  | Monoacylglycerol Lipase (MAGL) |
| *cqOC-C6-3/4/5/6/7/8/9/10* | BnaC06g22050D | AT1G75900 | GDSL |  |  |
| *cqOC-C6-3/4/5/6/7/8/9/10* | BnaC06g22680D | AT1G74960 | Plastidial Fatty Acid Synthesis | KASII; KASII (fab1) | Ketoacyl-ACP Synthase II |
| *cqOC-C6-3/4/5/6/7/8/9/10* | BnaC06g23310D | AT1G73920 | Lipase |  |  |
| *cqOC-C6-3/4/5/6/7/8/9/10* | BnaC06g23520D | AT1G73600 | Eukaryotic Phospholipid Synthesis |  | Phosphoethanolamine N-Methyltransferase |
| *cqOC-C6-3/4/5/6/7/8/9/10* | BnaC06g23560D | AT1G73480 | TAG Degradation |  | Monoacylglycerol Lipase (MAGL) |
| *cqOC-C6-3/4/5/6/7/8/9/10* | BnaC06g23880D | AT1G72970 | Cutin Synthesis | HTD | Omega-Hydroxy Fatty Acyl Dehydrogenase; omega-Hydroxy Fatty Acyl Dehydrogenase |
| *cqOC-C6-3/4/5/6/7/8/9/10* | BnaC06g24570D | AT1G70670 | TAG Synthesis |  | Caleosin |
| *cqOC-C6-3/4/5/6/7/8/9/10* | BnaC06g25920D | AT2G30550 | TAG degradation | FAD4 | Acylhydrolase (DAD1-like) |
| *cqOC-C6-3/4/5/6/7/8/9/10* | BnaC06g26190D | AT3G45140 | Lipid Signaling |  | Lipoxygenase |
| *cqOC-C6-3/4/5/6/7/8/9/10* | BnaC06g26670D | AT1G67560 | Lipase |  | Lipoxygenase |
| *cqOC-C6-3/4/5/6/7/8/9/10* | BnaC06g26980D | AT1G65060 | Aromatic Suberin Synthesis | At-4CL3 | 4-Coumarate-CoA Ligase |
| *cqOC-C6-3/4/5/6/7/8/9/10* | BnaC06g27120D | AT1G65580 | Lipid Signaling |  | Phosphoinositide 5-Phosphatase Type II |
| *cqOC-C6-3/4/5/6/7/8/9/10* | BnaC06g28830D | AT1G67730 | Fatty Acid Elongation and Cuticular Wax Synthesis | KCR1 | Ketoacyl-CoA Reductase |
| *cqOC-C6-3/4/5/6/7/8/9/10* | BnaC06g30250D | AT4G39670 | Sphingolipid Synthesis |  | Sphingosine Transfer Protein |
| *cqOC-C6-3/4/5/6/7/8/9/10* | BnaC06g30340D | AT1G68710 | Miscellaneous: lipid related |  | Translocase |
| *cqOC-C6-3/4/5/6/7/8/9/10* | BnaC06g31770D | AT1G70670 | TAG Synthesis |  | Caleosin |
| *cqOC-C6-3/4/5/6/7/8/9/10* | BnaC06g32150D | AT1G71010 | Lipid Signaling |  | Phosphatidylinositol-Phosphate Kinase type III |
| *cqOC-C6-3/4/5/6/7/8/9/10* | BnaC06g32670D | AT1G71697 | TAG Synthesis | CK1 | Choline Kinase |
| *cqOC-C6-3/4/5/6/7/8/9/10* | BnaC06g32970D | AT1G71960 | Cuticular Wax Synthesis | WBC26 / ABCG25 | ABC Transporter |
| *cqOC-C6-3/4/5/6/7/8/9/10* | BnaC06g33850D | AT1G72700 | Miscellaneous: lipid related |  | Translocase |
| *cqOC-C6-3/4/5/6/7/8/9/10* | BnaC06g34390D | AT1G73480 | TAG Degradation |  | Monoacylglycerol Lipase (MAGL) |
| *cqOC-C6-11* | BnaC06g35160D | AT1G74210 | Miscellaneous: lipid related |  | Glycerophosphoryl Diester Phosphodiesterase |
| *cqOC-C6-11* | BnaC06g35230D | AT1G74320 | TAG Synthesis |  | Choline Kinase |
| *cqOC-C6-11* | BnaC06g35760D | AT1G74960 | Plastidial Fatty Acid Synthesis | KASII; KASII (fab1) | Ketoacyl-ACP Synthase II |
| *cqOC-C8* | BnaC08g19000D | AT1G19640 | Lipid Signaling |  | Jasmonic Acid Carboxyl Methyltransferase |
| *cqOC-C8* | BnaC08g19190D | AT1G19800 | Lipid Trafficking | TGD1 | Permease-like Protein of Inner Chloroplast Envelope |
| *cqOC-C8* | BnaC08g19540D | AT4G30950 | Plastidial Glycerolipid, Galactolipid and Sulfolipid Synthesis | FAD6 | Oleate Desaturase |
| *cqOC-C8* | BnaC08g20070D | AT1G21980 | Lipid Signaling |  | Phosphatidylinositol-Phosphate Kinase type IB |
| *cqOC-C8* | BnaC08g20710D | AT3G49050 | Lipid acylhydrolase |  | Lipid Acylhydrolase-like |
| *cqOC-C8* | BnaC08g21900D | AT3G50400 | GDSL |  |  |
| *cqOC-C8* | BnaC08g22120D | AT3G50790 | Lipase |  |  |
| *cqOC-C8* | BnaC08g22310D | AT3G50920 | Plastidial Glycerolipid, Galactolipid and Sulfolipid Synthesis | LPP &epsilon;1 | Phosphatidate Phosphatase |
| *cqOC-C8* | BnaC08g22910D | AT3G51590 | Cuticular Wax Synthesis | LTP type 1 | Lipid Transfer Protein type 1 |
| *cqOC-C8* | BnaC08g22920D | AT3G51600 | Cuticular Wax Synthesis | LTP type 1 | Lipid Transfer Protein type 1 |
| *cqOC-C8* | BnaC08g23150D | AT3G51840 | beta-Oxidation | ACX4 | Acyl-CoA Oxidase |
| *cqOC-C8* | BnaC08g23460D | AT3G52130 | Cuticular Wax Synthesis | LTP type 3 | Lipid Transfer Protein type 3 |
| *cqOC-C8* | BnaC08g23660D | AT3G52430 | Lipase |  |  |
| *cqOC-C8* | BnaC08g24540D | AT3G53510 | Cuticular Wax Synthesis | WBC2 / ABCG2 | ABC Transporter |
| *cqOC-C8* | BnaC08g24920D | AT1G77590 | Plastidial Fatty Acid Synthesis | LACS9 | Long-Chain Acyl-CoA Synthetase (plastidial); Long-Chain Acyl-CoA Synthetase |
| *cqOC-C8* | BnaC08g25150D | AT3G54320 | Plastidial Fatty Acid Synthesis | WRI1 | AP2/ERWEBP Transcription Factors |
| *cqOC-C8* | BnaC08g25800D | AT3G54950 | Phospholipase |  | Acyl-Hydrolase (Patatin-like) |
| *cqOC-C8* | BnaC08g25880D | AT3G55030 | Eukaryotic Phospholipid Synthesis | PGPS2 | Phosphatidylglycerol-Phosphate Synthase |
| *cqOC-C8* | BnaC08g25990D | AT3G55110 | Cuticular Wax Synthesis | WBC18 / ABCG18 | ABC Transporter |
| *cqOC-C8* | BnaC08g26060D | AT3G55180 | TAG Degradation |  | Monoacylglycerol Lipase (MAGL) |
| *cqOC-C8* | BnaC08g26120D | AT3G55290 | Plastidial Fatty Acid Synthesis |  | Ketoacyl-ACP Reductase |
| *cqOC-C8* | BnaC08g26140D | AT3G55360 | Fatty Acid Elongation and Cuticular Wax Synthesis | ECR; ECR/CER1 | Enoyl-CoA Reductase |
| *cqOC-C8* | BnaC08g26860D | AT3G55940 | Phospholipase |  | Phosphoinositide-specific Phospholipase C |
| *cqOC-C8* | BnaC08g26960D | AT3G56040 | Plastidial Glycerolipid, Galactolipid and Sulfolipid Synthesis | UGPase, UGP3; UGP3 | UDP-Glucose Pyrophosphorylase; UDP-glucose pyrophosphorylase |
| *cqOC-C8* | BnaC08g26980D | AT3G56060 | Cutin Synthesis |  | Omega-Hydroxy Fatty Acyl Dehydrogenase; omega-Hydroxy Fatty Acyl Dehydrogenase |
| *cqOC-C8* | BnaC08g27500D | AT3G56600 | Lipid Signaling |  | Phosphatidylinositol-4-Kinase &gamma; |
| *cqOC-C8* | BnaC08g27570D | AT3G56700 | Cuticular Wax Synthesis | AlcFAR6 | Alcohol-forming Fatty Acyl-CoA Reductase |
| *cqOC-C8* | BnaC08g27580D | AT3G56700 | Cuticular Wax Synthesis | AlcFAR6 | Alcohol-forming Fatty Acyl-CoA Reductase |
| *cqOC-C8* | BnaC08g27860D | AT3G56960 | Lipid Signaling |  | Phosphatidylinositol-Phosphate Kinase type IB |
| *cqOC-C8* | BnaC08g27870D | AT3G56960 | Lipid Signaling |  | Phosphatidylinositol-Phosphate Kinase type IB |
| *cqOC-C8* | BnaC08g28660D | AT3G57650 | TAG Synthesis | LPAAT2 | 1-acylglycerol-3-phosphate acyltransferase; 1-Acylglycerol-3-Phosphate Acyltransferase |
| *cqOC-C8* | BnaC08g29950D | AT3G59770 | Lipid Signaling |  | Sac domain-containing Phosphoinositide Phosphatase |
| *cqOC-C8* | BnaC08g31440D | AT3G61580 | Sphingolipid Synthesis | SLD1 | Sphingobase-D8 Desaturase |
| *cqOC-C8* | BnaC08g32310D | AT3G62590 | Lipid acylhydrolase |  | Lipid Acylhydrolase-like |
| *cqOC-C8* | BnaC08g32530D | AT3G62860 | TAG Degradation |  | Monoacylglycerol Lipase (MAGL) |
| *cqOC-C8* | BnaC08g32750D | AT3G63200 | Phospholipase |  | Acyl-Hydrolase (Patatin-like) |
| *cqOC-C8* | BnaC08g32850D | AT3G51970 | Cuticular Wax Synthesis |  | Wax Synthase |
| *cqOC-C8* | BnaC08g32950D | AT2G26420 | Lipid Signaling |  | Phosphatidylinositol-Phosphate Kinase type IB |
| *cqOC-C8* | BnaC08g33100D | AT2G26250 | Fatty Acid Elongation and Cuticular Wax Synthesis | KCS1/FDH | Ketoacyl-CoA Synthase |
| *cqOC-C8* | BnaC08g33530D | AT2G25170 | TAG Synthesis | PKL | a SWI/SWF nuclear-localized chromatin remodeling factor of the CHD3 group |
| *cqOC-C8* | BnaC08g34430D | AT2G23540 | GDSL |  |  |
| *cqOC-C8* | BnaC08g34530D | AT2G23180 | Cuticular Wax Synthesis | CYP96A1 | Midchain Alkane Hydroxylase / Cytochrome P45, 96A |
| *cqOC-C8* | BnaC08g35260D | AT2G22230 | Plastidial Fatty Acid Synthesis | HAD | Hydroxyacyl-ACP Dehydrase |
| *cqOC-C8* | BnaC08g35290D | AT2G22170 | Lipase |  |  |
| *cqOC-C8* | BnaC08g35480D | AT2G21910 | Cuticular Wax Synthesis | CYP96A5 | Midchain Alkane Hydroxylase / Cytochrome P45, 96A |
| *cqOC-C8* | BnaC08g36080D | AT2G20960 | Phospholipase |  |  |
| *cqOC-C8* | BnaC08g36090D | AT2G20950 | Phospholipase |  |  |
| *cqOC-C8* | BnaC08g36120D | AT2G20900 | Lipid Signaling |  | Diacylglycerol Kinase |
| *cqOC-C8* | BnaC08g36170D | AT2G20860 | Mitochondrial Fatty Acid and Lipoic Acid Synthesis | LS or LIP1 | Lipoate Synthase |
| *cqOC-C8* | BnaC08g36500D | AT2G19880 | Sphingolipid Synthesis |  | Glucosylceramide Synthase (UDP-glucose-dependent) |
| *cqOC-C8* | BnaC08g36720D | AT1G19440 | Fatty Acid Elongation and Cuticular Wax Synthesis | KCS4 | Ketoacyl-CoA Synthase |
| *cqOC-C8* | BnaC08g37440D | AT1G76690 | Lipid Signaling |  | Oxo-Phytodienoic Acid Reductase |
| *cqOC-C9-2* | BnaC09g01080D | AT2G38110 | Cutin Synthesis | GPAT6 | Glycerol-3-Phosphate Acyltransferase (endomembrane predicted?) |
| *cqOC-C9-2* | BnaC09g01940D | AT3G28910 | Cuticular Wax Synthesis | MYB3 | Myb Transcription Factors |

| **Table S11. Potential candidates related to oilbody structural protein and seed storage protein identified in SPC-QTL genomic regions (SPC-QTLs co-localizing with SOC-QTLs are indicated in bold font).** | | | | | |
| --- | --- | --- | --- | --- | --- |
| **Gene alias** | **PC-QTL** | ***A. thaliana* Locus** | **Gene symbol** | **Type** | **Description** |
| BnaA03g38500D | *cqPC-A3-3* | AT2G13820 | XYP2 | seed storage 2S | Bifunctional inhibitor/lipid-transfer protein/seed storage 2S albumin superfamily protein |
| BnaA04g01950D | *cqPC-A4-1* | AT3G58550 |  | seed storage 2S | Bifunctional inhibitor/lipid-transfer protein/seed storage 2S albumin superfamily protein |
| BnaA04g19410D | *cqPC-A4-2* | AT2G33380 | CLO3 | oilbody structural protein | Caleosin CLO3-2 |
| BnaA09g02110D | ***cqPC-A9-2*** | AT3G27660 | OLEO4 | oilbody structural protein | Oleosin 4 |
| BnaA09g08190D | *cqPC-A9-3* | AT2G10940 |  | seed storage 2S | Bifunctional inhibitor/lipid-transfer protein/seed storage 2S albumin superfamily protein |
| BnaA09g13220D | ***cqPC-A9-4*** | AT1G62510 |  | seed storage 2S | Bifunctional inhibitor/lipid-transfer protein/seed storage 2S albumin superfamily protein |
| BnaA09g11520D | ***cqPC-A9-5*** | AT1G64235 |  | seed storage 2S | Bifunctional inhibitor/lipid-transfer protein/seed storage 2S albumin superfamily protein |
| BnaC03g65080D | *cqPC-C3-3* | AT4G22666 |  | seed storage 2S | Bifunctional inhibitor/lipid-transfer protein/seed storage 2S albumin superfamily protein |
| BnaC05g02160D | ***cqPC-C5-1*** | AT1G03880 | CRU2 | cruciferin | cruciferin 2 (CRU2); 12S storage protein |
| BnaC05g44560D | ***cqPC-C5-5*** | AT2G28680 |  | cupin | RmlC-like cupins superfamily protein |
| BnaC05g44510D | ***cqPC-C5-5*** | AT3G07450 |  | seed storage 2S | Bifunctional inhibitor/lipid-transfer protein/seed storage 2S albumin superfamily protein |
| BnaC06g18820D | ***cqPC-C6-2*** | AT3G63095 |  | seed storage | Protease inhibitor/seed storage/LTP family protein |
| BnaC08g09910D | *cqPC-C8* | AT4G14805 |  | seed storage 2S | Bifunctional inhibitor/lipid-transfer protein/seed storage 2S albumin superfamily protein |

| **Table S12. Co-localization with QTLs for oil content in other segregating populations with different genetic backgrounds (SOC-QTL marked in red are identified in the present linkage map).** | | | | |
| --- | --- | --- | --- | --- |
| **Co-localized QTLs** | **Chromosome** | **QTL region** | **Flanking markers** | **Genomic region (Mb)** |
| ***qOC-A1-SO*** | A01 | 68–79 | 3150404|F|0/3093342|F|0 | 0.59 ~ 20.04 |
| ***qOC-A1-SG*** | A01 | 49.9-66.9 | ZAASA1-73/Sca14-8 | 5.74 ~ 7.49 |
| ***qOC-A1-KN*** | A01 | 104.00-112.10 | Bn_A01_p0091/Bn_A01_p0189_A1 | 6.016 ~ 8.053 |
| ***qOC-A1-3-TN*** | A01 | 70.80-76.06 | IGF9014a/CNU132 | 6.24 ~ 10.35 |
| ***qOC-A3-2-Z5*** | A03 | 25.2-30.1 | BrGMS4497/SF49160 | 8.99 ~ 14.92 |
| ***qOC-A3-3-KN*** | A03 | 120.45 - 124.29 | Bns_p2873 / Bn_A03_p0721_A3 | 14.32 ~ 16.07 |
| ***qOC-A3-DY*** | A03 | 0-31.3 | Bras002b/CB10415 | 13.45 ~ 18.52 |
| ***qOC-A3-3-TN*** | A03 | 59.38-70.40 | CNU098/CNU210 | 13.54 ~ 15.23 |
| ***qOC-A3-RNSL*** | A03 | 13.2-62.2 | AgCan9/SN11722a | 15.09 ~ 17.65 |
| ***qOC-A3-4-TN*** | A03 | 72.44-93.81 | S13M08-1-157/CNU371 | 15.86 ~ 20.77 |
| ***qOC-A3-5-TN*** | A03 | 97.24-108.84 | CNU223/HAU61-1 | 20.87 ~ 21.95 |
| ***qOC-A3-4-KN*** | A03 | 150.70-153.90 | Bn_A03_p0586 / Bns_p3760_A3 | 21.36 ~ 21.86 |
| ***qOC-A8-1-TN*** | A08 | 59.44-79.12 | IGF1108c/CNU090 | 8.03 ~ 14.11 |
| ***qOC-A8-RNSL*** | A08 | 33.6-69.8 | AgCan48/Na12B05 | 8.03 ~ 15.4 |
| ***qOC-A8-2-KN*** | A08 | 7.34 - 9.88 | Bns_p2739_A8 / Bns_p2735_A8 | 9.198 ~ 10.83 |
| ***qOC-A8-3-KN*** | A08 | 10.85 - 13.78 | Bns_p2735_A8 / Bn_A08_p1589_A8 | 10.37 ~ 12.21 |
| ***qOC-A8-5-KN*** | A08 | 19.53 - 20.49 | Bn_A08_p1619 / Bn_A08_p1587_A8 | 11.06 ~ 11.9 |
| ***qOC-A8-4-KN*** | A08 | 17.61 - 18.41 | Bn_A08_p1586_A8 / Bn_A08_p1618_A8 | 11.85 ~ 12.21 |
| ***qOC-A8-6-KN*** | A08 | 20.4-24.2 | Bn_A08_p1585_A8 / Bn_A08_p1590 | 11.96 ~ 12.81 |
| ***qOC-A9-1-KN*** | A09 | 21.81 - 24.73 | Bns_p2779_A9 / Bn_A09_p1846_A9 | 3.264 ~ 4.344 |
| ***qOC-A9-4-M201xM202*** | A09 | 39.5–43.5 | BrGMS25/BnEMS799c | 3.452186 ~ 28.26 |
| ***qOC-A9-SG*** | A09 | 0.0-17.3 | E6M2a/sN0240A | 4.13 ~ 12.28 |
| ***qOC-A9-4-M201xM202*** | A09 | 39.5–43.5 | BrGMS25/BnEMS799c | 3.452186 ~ 28.26 |
| ***qOC-A9-SG*** | A09 | 0.0-17.3 | E6M2a/sN0240A | 4.13 ~ 12.28 |
| ***qOC-A9-2-KN*** | A09 | 30.4-32.2 | Bn_A09_p1750 / Bn_A09_p1805 | 5.878 ~ 6.354 |
| ***qOC-A9-4-M201xM202*** | A09 | 39.5–43.5 | BrGMS25/BnEMS799c | 3.452186 ~ 28.26 |
| ***qOC-A9-SG*** | A09 | 0.0-17.3 | E6M2a/sN0240A | 4.13 ~ 12.28 |
| ***qOC-A9-3-KN*** | A09 | 32.36 - 36.17 | Bn_A09_p1805 / Bn_A09_p1823_A9 | 6.354 ~ 9.293 |
| ***qOC-A9-3-TN*** | A09 | 92.79-99.30 | pX150/CNU296 | 24.48 ~ 27.13 |
| ***qOC-A9-4-KN*** | A09 | 67.1-73.1 | Bn_A05_p0981_A9 / Bn_A09_p1694_A9 | 24.78 ~ 26 |
| ***qOC-A9-7-KN*** | A09 | 99.9-101.2 | Bn_A09_p1700 / Bn_A09_p1713_A9 | 28.68 ~ 29.66 |
| ***qOC-A9-4-TN*** | A09 | 115.49-121.21 | CNU263/pW123bE | 29.49 ~ 31.23 |
| ***qOC-A9-4-TN*** | A09 | 115.49-121.21 | CNU263/pW123bE | 29.49 ~ 31.23 |
| ***qOC-A9-8-KN*** | A09 | 105.79 - 106.77 | Bns_p2574_A9 / Bn_A09_p1830_A9 | 30.61 ~ 30.68 |
| ***qOC-A9-4-TN*** | A09 | 115.49-121.21 | CNU263/pW123bE | 29.49 ~ 31.23 |
| ***qOC-A9-9-KN*** | A09 | 111.62 - 113.79 | Bns_p2570 / Bn_A09_p1707_A9 | 31.09 ~ 33.75 |
| ***qOC-A10-2-KN*** | A10 | 5.8-16.7 | Bn_A10_p2015 / niab144-A10 | 0.821 ~ 13.91 |
| ***qOC-A10-TN*** | A10 | 25.54-38.27 | niab34/RA2E03 | 6.25 ~ 11 |
| ***qOC-A10-DY*** | A10 | 79.2-111.3 | MR156/Bras001 | 7.13 ~ 7.13 |
| ***qOC-A10-2-KN*** | A10 | 5.8-16.7 | Bn_A10_p2015 / niab144-A10 | 0.821 ~ 13.91 |
| ***qOC-A10-TN*** | A10 | 25.54-38.27 | niab34/RA2E03 | 6.25 ~ 11 |
| ***qOC-C2-2-KN*** | C02 | 89.17 - 93.04 | Bns_p2329_C2 / Bns_p3492_C2 | 39 ~ 41.76 |
| ***qOC-C2-4-TN*** | C02 | 75.88-81.99 | IGF3246z/sORA43 | 39.91 ~ 41.95 |
| ***qOC-C2-3-KN*** | C02 | 97.6-103.1 | Bns_p2762 / Bns_p2933 | 40.35 ~ 43.54 |
| ***qOC-C2-3-KN*** | C02 | 97.6-103.1 | Bns_p2762 / Bns_p2933 | 40.35 ~ 43.54 |
| ***qOC-C2-1-DY*** | C02 | 0-43.5 | CB10543a/CZ0b716882 | 43.34 ~ 43.34 |
| ***qOC-C3-SO*** | C03 | 34–66 | brPb-659400/3132883|F|0 | 1.78 ~ 5.03 |
| ***qOC-C3-2-KN*** | C03 | 163.56 - 168.65 | Bns_p3685 / Bns_p3238 | 2.63 ~ 3.386 |
| ***qOC-C3-SO*** | C03 | 34–66 | brPb-659400/3132883|F|0 | 1.78 ~ 5.03 |
| ***qOC-C3-3-KN*** | C03 | 171.6-173.9 | Bn_A03_p0598_C3 / Bns_p3459_C3 | 4.06 ~ 5.014 |
| ***qOC-C3-1-TN*** | C03 | 125.78-130.22 | BRAS068/IGF0235b | 50.72 ~ 53.75 |
| ***qOC-C3-4-KN*** | C03 | 299.84 - 301.12 | Bns_p2064_C3 / Bns_p3674 | 52.35 ~ 52.62 |
| ***qOC-C3-7-KN*** | C03 | 314.17 - 322.64 | Bns_p2183_C3 / Bns_p3245_C3 | 56.37 ~ 58.52 |
| ***qOC-C3-3-TN*** | C03 | 135.51-149.99 | Ol13H09/IGF1152z | 58.29 ~ 58.29 |
| ***qOC-C6-DY*** | C06 | 18-46.1 | K11.750/CB10526 | 13.14 ~ 20.25 |
| ***qOC-C6-SG*** | C06 | 14.8-33.7 | ZAAS815b/ZAAS763 | 18.41 ~ 22.96 |
| ***qOC-C6-1-KN*** | C06 | 147.65 - 151.02 | Bns_p3085_C6 / Bns_p2208 | 18.72 ~ 21.8 |
| ***qOC-C8-KN*** | C08 | 62.66 - 69.65 | Bns_p2503_C8 / Bns_p2572_C8 | 29.29 ~ 34.28 |
| ***qOC-C8-RNSL*** | C08 | 90.5-91 | CB10179/SN11670 | 31.4 ~ 31.81 |

| **Table S13. The primers for the six target genes and reference gene Actin.** | | |
| --- | --- | --- |
| **gene** | **Forward** | **Reverse** |
| BnaA08g12780D | GATATGGTCCAATCCGGCCTT | ACCCTATTCACTTCGCTTGGTC |
| BnaA09g48250D | CCATTGTCTCCCTTCCCTCT | GCACCACCACCACTTTTTCT |
| BnaC03g65980D | TTCGTTACCTTCATGGGCAAG | AGCACATCAATCACGGCTT |
| BnaC03g67820D | TATCTGCTTCCGTTGGCTTG | ATGTTGGTTTTGGCGTGATG |
| BnaC05g43390D | ATTTGGTAACAGAGACACCGAT | TCCGATGTCCTGTTGCTACCTC |
| BnaC05g44510D | GTCGGCTTTGATTCAACTCGT | AGCACATAGACAAGGTTGACCA |
| Actin | CCCTGGAATTGCTGACCGTA | TGGAAAGTGCTGAGGGATGC |

**Additional Information**

**Competing financial interests**: The authors declare no competing financial interests.
